# Spatial Pattern Separation Testing Differentiates Alzheimer’s Disease Biomarker-Positive and Biomarker-Negative Older Adults With Amnestic Mild Cognitive Impairment

**DOI:** 10.3389/fnagi.2021.774600

**Published:** 2021-11-26

**Authors:** Martina Laczó, Ondrej Lerch, Lukas Martinkovic, Jana Kalinova, Hana Markova, Martin Vyhnalek, Jakub Hort, Jan Laczó

**Affiliations:** ^1^Memory Clinic, Department of Neurology, Charles University, Second Faculty of Medicine and Motol University Hospital, Prague, Czechia; ^2^International Clinical Research Center, St. Anne’s University Hospital Brno, Brno, Czechia

**Keywords:** amyloid-β, basal forebrain, cerebrospinal fluid, entorhinal cortex, hippocampus, memory, magnetic resonance imaging, positron emission tomography

## Abstract

**Background:** The hippocampus, entorhinal cortex (EC), and basal forebrain (BF) are among the earliest regions affected by Alzheimer’s disease (AD) pathology. They play an essential role in spatial pattern separation, a process critical for accurate discrimination between similar locations.

**Objective:** We examined differences in spatial pattern separation performance between older adults with amnestic mild cognitive impairment (aMCI) with AD versus those with non-Alzheimer’s pathologic change (non-AD) and interrelations between volumes of the hippocampal, EC subregions and BF nuclei projecting to these subregions (medial septal nuclei and vertical limb of the diagonal band of Broca – Ch1-2 nuclei) with respect to performance.

**Methods:** Hundred and eighteen older adults were recruited from the Czech Brain Aging Study. Participants with AD aMCI (*n* = 37), non-AD aMCI (*n* = 26), mild AD dementia (*n* = 26), and cognitively normal older adults (CN; *n* = 29) underwent spatial pattern separation testing, cognitive assessment and brain magnetic resonance imaging.

**Results:** The AD aMCI group had less accurate spatial pattern separation performance than the non-AD aMCI (*p* = 0.039) and CN (*p* < 0.001) groups. The AD aMCI and non-AD groups did not differ in other cognitive tests. Decreased BF Ch1-2 volume was indirectly associated with worse performance through reduced hippocampal tail volume and reduced posteromedial EC and hippocampal tail or body volumes operating in serial.

**Conclusion:** The study demonstrates that spatial pattern separation testing differentiates AD biomarker positive and negative older adults with aMCI and provides evidence that BF Ch1-2 nuclei influence spatial pattern separation through the posteromedial EC and the posterior hippocampus.

## Introduction

Alzheimer’s disease (AD) is the most common age-related neurodegenerative disease characterized by gradual episodic and spatial memory decline. Memory decline in AD is caused by impaired encoding and retention of information (Larrabee et al., 1993; Ally et al., 2013). A neural process of encoding similar information as non-overlapping memories to-be-recalled separately from each other is referred to as pattern separation (Yassa and Stark, 2011; Ally et al., 2013; Reagh et al., 2014). The behavioral outcome of this process is referred to as mnemonic discrimination that includes discrimination between visually similar objects (i.e., object mnemonic discrimination/object behavioral pattern separation) and similar locations (i.e., spatial mnemonic discrimination/spatial behavioral pattern separation) (Leal and Yassa, 2018).

The hippocampus, especially the dentate gyrus (DG), plays a key role in pattern separation (Hunsaker and Kesner, 2008, 2013) and receives information about objects and locations from the entorhinal cortex (EC) through the perforant path (Yassa et al., 2011b; Hunsaker and Kesner, 2013). Within the EC, two subregions that are part of distinct information-processing pathways have been identified in humans (Maass et al., 2015). The anterolateral EC (alEC) is involved in object information processing and receives projections from the perirhinal cortex (PrC) and anterior cortical regions. In contrast, the posteromedial EC (pmEC) is involved in spatial information processing and receives projections from the parahippocampal cortex (PhC) and posterior-medial cortical regions (Navarro Schröder et al., 2015; Berron et al., 2018). These two pathways convey information to the hippocampus and contribute to object and spatial pattern separation processes (Reagh and Yassa, 2014). Recent studies found that the alEC also receives projections from the PhC (Doan et al., 2019; Nilssen et al., 2019) indicating that connectivity within the medial temporal lobe (MTL) regions may be more complex. Within the hippocampus, different connectivity and functional specialization has been described for the anterior and posterior subregions. The anterior hippocampus (i.e., the head) is preferentially connected to the PrC (Libby et al., 2012) and supports object information processing (Pihlajamäki et al., 2004; Lee et al., 2008), while the posterior hippocampus (i.e., the body and tail) is preferentially connected to the PhC (Libby et al., 2012) and supports spatial information processing (Pihlajamäki et al., 2004; Lee et al., 2008), especially processing of detailed spatial information (Nadel et al., 2013) and fine spatial discrimination (McTighe et al., 2009).

Pattern separation declines with aging, with object discrimination being particularly vulnerable (Yassa et al., 2011a; Stark et al., 2013). Object pattern separation deficits observed with aging may be related to changes of integrity in the hippocampal DG and CA3 subfields (Yassa et al., 2011a, b), perforant path (Yassa et al., 2011b), PrC (Ryan et al., 2012; Berron et al., 2018), alEC (Reagh et al., 2018), and alEC-hippocampus network (Berron et al., 2019). The PrC, alEC and the anterior hippocampus are among the earliest regions where tau neurofibrillary tangles emerge (Braak and Braak, 1991) and therefore tau pathology has been suggested as a likely culprit of age-related decline in object pattern separation (Berron et al., 2018). Accordingly, recent *in vivo* studies indicated that tau accumulation affecting predominantly the anterior-temporal system (Maass et al., 2019) and high levels of phosphorylated tau in cerebrospinal fluid (CSF) (Berron et al., 2019) are associated with object discrimination deficits in older adults. Accumulation of hyperphosphorylated tau in the PrC, alEC, and the anterior hippocampus is commonly found in older adults (Braak and Braak, 1997) and is considered a major pathological marker of AD together with neocortical amyloid-β plaques deposition (Hyman et al., 2012). Similar pattern of regional tau deposition in these MTL regions without amyloid-β pathology has been observed in other neurodegenerative diseases including argyrophilic grain disease (Ferrer et al., 2008) and primary age-related tauopathy (Crary et al., 2014), where tau pathology is related to structural changes in the anterior hippocampus (Josephs et al., 2017). In summary, age-related object pattern separation deficits associated with changes in the PrC-alEC-anterior hippocampus network may be caused by early tau accumulation in the MTL that is found in healthy older adults, as well as in other neurodegenerative diseases including AD. Thus, object discrimination deficits seem to be an early marker of tau pathology and may be the earliest but a non-specific cognitive marker of AD.

It has been suggested that spatial discrimination is less impaired than object discrimination in healthy aging (Reagh et al., 2016; Güsten et al., 2021). Recent studies also indicated that, unlike object discrimination, spatial discrimination deficits may not be associated with tau pathology measured by phosphorylated tau in CSF (Berron et al., 2019) and tau tracer uptake in anterior-temporal regions (Maass et al., 2019) in cognitively normal older adults. However, spatial discrimination deficits were associated with higher cortical amyloid-β accumulation (Webb et al., 2020), especially in the posterior-medial regions (Maass et al., 2019). The posterior-medial regions, including the retrosplenial cortex, posterior cingulate cortex and precuneus, are strongly involved in spatial information processing (Kravitz et al., 2011). The precuneus and the posterior cingulate cortex including the retrosplenial cortex (Pengas et al., 2010) are also the earliest and predominant sites of amyloid-β accumulation in AD (Palmqvist et al., 2017). These regions are closely interconnected with the MTL, especially PhC (Burwell, 2000), pmEC (Navarro Schröder et al., 2015), and the posterior hippocampus (Aggleton et al., 2012), which plays a key role in spatial information processing and discrimination (Pihlajamäki et al., 2004; Lee et al., 2008; McTighe et al., 2009) and seems to be more vulnerable to AD-related structural changes than the anterior hippocampus (Lindberg et al., 2017; Lladó et al., 2018). These findings may indicate that spatial pattern separation deficits are associated with amyloid-β pathology and could be a relatively specific cognitive marker of AD. It remains unknown how early spatial discrimination deficits emerge in the course of AD, especially in preclinical AD. However, the recent studies showed that the apolipoprotein E ε4 allele, the strongest known genetic risk factor for sporadic AD (Saunders et al., 1996) associated with increased cerebral amyloid-β deposition (Fleisher et al., 2013), is related to more pronounced spatial discrimination deficits in older adults (Sheppard et al., 2016) and reduced hippocampal recruitment during a spatial discrimination task in young pre-symptomatic individuals (Lee et al., 2020).

The results of recent studies indicate that spatial memory testing can differentiate older adults with mild cognitive impairment (MCI) with and without positivity of amyloid-β (Schöberl et al., 2020) and CSF AD biomarkers (Howett et al., 2019). These studies also showed that spatial memory deficits are associated with reduced activation of the hippocampus and posterior-medial regions (Schöberl et al., 2020) and decreased volume of the pmEC (Howett et al., 2019). Our previous study showed that spatial pattern separation is impaired in biomarker-defined amnestic MCI (aMCI) participants with AD (AD aMCI) above and beyond general memory and other cognitive deficits and gradually declines with increasing disease severity and decreasing hippocampal and EC volumes (Parizkova et al., 2020). Our results also indicated that spatial pattern separation testing may be used to detect early cognitive decline in AD and reflect hippocampal and EC atrophy. However, it has not been established whether spatial pattern separation testing might be a promising way to differentiate between older adults with AD aMCI and those with aMCI and non-Alzheimer’s pathologic change (non-AD aMCI) and might reflect structural changes in the specific hippocampal and EC subregions.

Hippocampal and EC function, including pattern separation, is modulated by acetylcholine (Hunsaker and Kesner, 2013), with high levels of acetylcholine increase discrimination ability (Giocomo and Hasselmo, 2007). The majority of cholinergic projections targeting the hippocampus and EC originate in the basal forebrain (BF), specifically in the medial septal nuclei and the vertical limb of the diagonal band of Broca (i.e., Ch1-2 nuclei) (Kondo and Zaborszky, 2016), and lesions of the BF cholinergic fornical projections result in less effective spatial pattern separation in rodents (Ikonen et al., 2002). The BF nuclei are among the earliest regions where AD pathology emerges (Sassin et al., 2000; Geula et al., 2008). BF nuclei degeneration was shown to be associated with cortical amyloid-β accumulation in preclinical AD and MCI with AD (Grothe et al., 2014), precede and predict EC pathology in preclinical AD (Schmitz et al., 2016) and predict longitudinal EC degeneration in older adults with positive CSF AD biomarkers (Fernández-Cabello et al., 2020). Our previous study (Parizkova et al., 2020) showed that less accurate spatial pattern separation is associated with atrophy of the BF nuclei, specifically the Ch1-2 nuclei, in participants with biomarker-defined early clinical AD. However, the specific pathways and hippocampal and EC subregions through which BF Ch1-2 nuclei may affect spatial pattern separation have not been determined.

We built on our previous findings of spatial pattern separation deficits in biomarker-defined early clinical AD and their associations with hippocampal, EC and BF nuclei volumes. We aimed to further extend these findings by assessing: (1) the differences in spatial pattern separation performance between participants with AD aMCI and non-AD aMCI, (2) the associations of spatial pattern separation performance with volumes of specific hippocampal and EC subregions and BF Ch1-2 nuclei, and (3) the interrelations between these regions with respect to spatial pattern separation performance.

We hypothesized that: (1) the participants with AD aMCI would have less accurate spatial pattern separation performance than the participants with non-AD aMCI; (2) worse spatial pattern separation performance would be associated with decreased volumes of the posterior hippocampus (i.e., tail and body), the pmEC and the BF Ch1-2 nuclei; and (3) the association between BFCh1-2 nuclei volume and spatial pattern separation performance would be mediated by volumes of the posterior hippocampus and the pmEC.

## Materials and Methods

### Participants

#### Recruitment and Inclusion Criteria

A total of 118 participants were recruited from the Czech Brain Aging Study cohort (Sheardova et al., 2019) at the Memory Clinic of the Charles University, Second Faculty of Medicine and Motol University Hospital in Prague, Czechia and signed an informed consent approved by the local ethics committee (Parizkova et al., 2018). The participants with cognitive deficit were referred to the Memory Clinic by general practitioners and neurologists for memory complaints reported by themselves and their informants. Cognitively normal (CN) older adults were recruited from the University of the Third Age, senior centers (e.g., the Elpida center) and relatives of the participants and hospital staff.

All participants underwent clinical and laboratory evaluations, comprehensive cognitive assessment, brain magnetic resonance imaging (MRI) and spatial pattern separation task. The participants with cognitive deficit underwent biomarker assessment including analysis of amyloid-β_1__–__42_, total tau and phosphorylated tau_181_ (p-tau_181_) in CSF and/or amyloid PET imaging.

(i) Participants with AD aMCI (*n* = 37) met the clinical criteria for aMCI (Albert et al., 2011) including memory complaints, evidence of memory impairment (i.e., score lower than 1.5 standard deviations [SDs] below the age- and education-adjusted norms in any memory test), generally intact activities of daily living and absence of dementia. The participants had positive CSF AD biomarkers (reduced amyloid-β_1__–__42_ and elevated *p*-tau_181_ [<665 pg/ml and >48 pg/ml, respectively, the internally validated cut-offs], Parizkova et al., 2018) (*n* = 25) and/or positive amyloid PET imaging (positive visual read of 18F-flutemetamol PET scan) (*n* = 20).
(ii) Participants with non-AD aMCI (*n* = 26) met the clinical criteria for aMCI (Albert et al., 2011) and have negative amyloid-β biomarkers defined as normal CSF amyloid-β_1__–__42_ (≥665 pg/ml) (*n* = 13) and/or negative amyloid PET imaging (*n* = 22) according to the NIA-AA research framework recommendations (Jack et al., 2018).
(iii) Participants with mild AD dementia (*n* = 26) met the clinical criteria for dementia (Mckhann et al., 2011) with evidence of progressive cognitive impairment in at least two cognitive domains including memory (i.e., score lower than 1.5 SDs below the age- and education-adjusted norms in any memory test and in at least one other non-memory cognitive test) and significant impairment in activities of daily living. The participants had positive CSF AD biomarkers (reduced amyloid-β_1__–__42_ and elevated *p*-tau_181_ [<665 pg/ml and >48 pg/ml, respectively], Parizkova et al., 2018) (*n* = 22) and/or positive amyloid PET imaging (*n* = 12).
(iv) CN participants (*n* = 29) did not report any cognitive complaints, had cognitive performance within the normal range (i.e., score higher than 1.5 SDs below the age- and education-adjusted norms in any cognitive test). In addition, they had no evidence of MTL atrophy on MRI and did not have family history of AD or other type of dementia in the first-degree relatives. These stringent criteria were applied to minimize the possibility of including participants with preclinical and early clinical AD.

#### Exclusion Criteria

Participants with depressive symptoms (≥6 points on the 15-item Geriatric Depression Scale [GDS-15]), anxiety (≥10 points on the Beck Anxiety Inventory [BAI]), low visual acuity (<20/40 [corrected] on visual acuity tests), moderate to severe white matter vascular lesions on MRI (Fazekas score > 2 points) and other primary neurological or psychiatric disorders and those who did not complete the Spatial pattern separation task were not included in the study. Participants with cognitive deficit who did not have biomarker assessment were also not included.

### Spatial Pattern Separation Task

**FIGURE 1:**
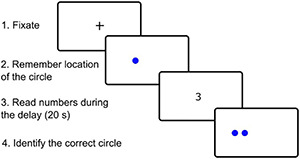
Example of spatial pattern separation task in one of 32 trials as seen by participants on the computer screen.

We used the recently published spatial pattern separation task (Parizkova et al., 2020) that was adapted from the previous study (Holden et al., 2012) and whose scheme is presented in Figure 1. The task was run on a computer with a 24″ monitor and consisted of 32 trials. Each trial started with a sample phase followed by a choice phase. In the sample phase, the participants were instructed to remember the location of a blue circle on the screen. The circle measuring 2 cm in diameter appeared for 5 s in one of 18 possible locations within an invisible horizontal line across the middle of the screen. During the choice phase, two identical blue circles were displayed. One of the circles, the target circle, was in the same location as the original circle in the sample phase (correct choice). The foil circle was located either to the left or right from the original circle (incorrect choice). Four possible spatial separations were used to separate the target and foil circles during the choice phase: 0 (edges of the circles were touching), 0.5, 1.0, and 1.5 cm. During the choice phase, the participants had to identify the target circle by pressing a green button in the right hand if the target circle was the right one of the two circles or pressing a red button in the left hand if the target circle was the left one. During a time delay of 20 s between the sample and choice phases, the participants were instructed to look in the middle of the screen and to read aloud a randomly appearing string of numbers to prevent the participants from fixating the eyes on the location of the original circle. After the choice phase, a little cross separating the trials appeared in the middle of the screen for 3 s and the participants were instructed to look at it. There were eight trials for each separation distance (0, 0.5, 1.0, and 1.5 cm). The whole task consisting of 32 trials lasted 17 min and was split into two sets of 16 trials. There was a five-minute break between these two sets to minimize fatigue of the participants. Spatial separation distance and location of the target circle on a particular side (left or right) were pseudo-randomized.

All participants completed familiarization training consisting of four trials prior to the testing. The training was repeated in case of any error or missed response. Participants who did not understand the task during the familiarization training (*n* = 5) or missed more than four responses in the testing phase (*n* = 4) were not included in the study. These participants were from the mild AD dementia group. All CN and aMCI participants managed to complete the task.

### Cognitive Assessment

The cognitive assessment included the following tests: (1) verbal memory measured with the Rey Auditory Verbal Learning Test (RAVLT) – trials 1–5 and 30-min Delayed Recall trial and Enhanced Cued Recall test – Free Recall and Total Recall trials; (2) non-verbal memory measured with the Rey-Osterrieth Complex Figure Test (ROCFT) – the Recall condition after 3 min; (3) visuospatial function measured with the ROCFT – the Copy condition and the Clock Drawing Test; (4) executive function measured with the Trail Making Test (TMT) B and Controlled Oral Word Association Test (Czech version with letters N, K, and P); (5) attention and working memory measured with the Forward and Backward Digit Spans and TMT A; and (6) language measured with the Boston Naming Test (30-item version) and Semantic Verbal Fluency test (Animals). The Mini-Mental State Examination (MMSE) was administered to measure global cognitive function. The GDS-15 and BAI were used to assess depressive symptoms and anxiety among participants. Group-wise neuropsychological characteristics are listed in Table 1.

**TABLE 1:** Characteristics of study participants.

**Variables**	**CN (*n* = 29)**	**Non-AD aMCI (*n* = 26)**	**AD aMCI (*n* = 37)**	**Mild AD dementia (*n* = 26)**	***P*-values**	**Effect sizes**
*Demographic characteristics*						
Age (years)	70.17 (6.33)	70.46 (8.43)	71.70 (6.78)	71.58 (4.52)	0.748	0.01
Women, *n* (%)	23 (79)	12 (46)	19 (51)	19 (73)	0.022	0.08
Education (years)	16.34 (2.02)*^c^	14.38 (2.68)	15.05 (3.07)	13.81 (3.26)	0.008	0.01
*Spatial pattern separation*						
Pattern Separation (% correct)	85.45 (7.67)*^a,^***^b,c^	74.76 (12.65)*^b,^***^c^	65.20 (15.06)	57.81 (16.66)	<0.001	0.37
*Cognitive assessment*						
MMSE (score)	29.69 (0.60)***^a–c^	27.54 (2.45)***^c^	26.81(1.91)***^c^	22.46 (2.37)	<0.001	0.63
GDS-15 (score)	0.67 (0.96)***^a,c^	3.09 (2.52)	2.06 (1.58)	3.35 (2.67)	<0.001	0.21
BAI (score)	5.17 (4.86)	7.65 (6.42)	7.19 (8.05)	9.04 (6.95)	0.274	0.04
RAVLT 1-5 (score)	54.33 (8.97)***^a–c^	36.36 (7.77)**^c^	34.06 (9.11)*^c^	26.25 (6.89)	<0.001	0.58
RAVLT 30 (score)	11.37 (3.08)***^a–c^	4.59 (2.94)*^c^	3.29 (3.20)	1.33 (2.57)	<0.001	0.61
ECR-FR (score)	11.14 (1.35)**^a,^***^b,c^	6.00 (3.94)*^c^	4.33 (2.65)*^c^	2.08 (2.13)	<0.001	0.59
ECR-TR (score)	14.29 (4.54)*^c^	12.40 (2.79)	10.81 (4.06)	9.19 (4.07)	0.026	0.15
TMT A (seconds)	32.00 (9.51)*^c^	58.40 (20.03)	56.00 (26.68)	76.38 (47.47)	0.027	0.27
TMT B (seconds)	80.36 (16.30)***^b,c^	164.14 (40.37)	224.94 (84.84)	244.93 (122.06)	<0.001	0.38
COWAT (score)	47.71 (8.86)*^a,^**^c^	38.09 (10.21)	44.49 (12.93)*^c^	36.69 (10.79)	0.001	0.14
ROCFT-C (score)	32.39 (2.48)***^b,^**^c^	28.86 (3.32)	27.12 (5.04)	27.39 (5.78)	<0.001	0.20
ROCFT-R (score)	21.46 (5.27)***^a–c^	9.15 (6.20)	6.14 (5.16)	4.39 (5.25)	<0.001	0.59
DSF (score)	9.21 (1.77)	8.52 (1.86)	8.95 (2.09)	8.15 (1.59)	0.191	0.04
DSB (score)	6.46 (1.72)*^c^	5.35 (2.01)	5.65 (1.74)	5.08 (1.57)	0.042	0.07
CDT (score)	15.42 (0.93)**^b,^***^c^	14.39 (1.64)*^c^	13.49 (2.43)	12.77 (2.60)	<0.001	0.18
SVF Animals (score)	26.79 (5.45)***^a–c^	20.22 (5.82)	19.30 (5.14)	16.42 (4.20)	<0.001	0.34
BNT (no. of errors)	1.25 (1.15)**^a,b,^***^c^	4.35 (3.71)	4.30 (2.48)	4.02 (3.47)	<0.001	0.25
*CSF analysis* ^d^						
Amyloid-β_1__–__42_ (pg/ml)	–	982.54 (276.22)***^b,c^	457.55 (101.97)	430.39 (110.97)	<0.001	0.62
Amyloid-β_1__–__42_ (pg/ml) range	*–*	666.90 – 1734.00	252.90 – 637.00	225.90 – 643.00	–	–
*p*-tau_181_ (pg/ml)	–	49.47 (14.31)**^b^	110.10 (65.56)	82.39 (27.14)	0.020	0.24
*p*-tau_181_ (pg/ml) range	–	25.00–72.00	49.70 – 358.80	48.2 – 139.20	–	–
*MRI measures* ^e^						
Hippocampal head^f^ (cm^3^) Right/Left	3.19 (0.45)*^b,^**^c^ 1.64 (0.23)/1.53 (0.23)	3.01 (0.55) 1.58 (0.28)/1.43 (0.28)	2.87 (0.40) 1.49 (0.23)/1.37 (0.22)	2.76 (0.33) 1.43 (0.19)/1.32 (0.15)	0.005	0.12
Hippocampal body^f^ (cm^3^) Right/Left	1.96 (0.20)***^b,c^ 0.95 (0.10)/1.02 (0.11)	1.82 (0.35)*^c^ 0.89 (0.18)/0.93 (0.18)	1.66 (0.26) 0.82 (0.14)/0.84 (0.15)	1.57 (0.22) 0.78 (0.11)/0.80 (0.12)	< 0.001	0.25
Hippocampal tail^f^ (cm^3^) Right/Left	0.59 (0.09)***^b,c^ 0.29 (0.05)/0.31 (0.04)	0.56 (0.09)**^b,^***^c^ 0.26 (0.04)/0.29 (0.05)	0.48 (0.08) 0.23 (0.05)/0.24 (0.05)	0.43 (0.09) 0.21 (0.04)/0.22 (0.06)	<0.001	0.32
alEC^f^ (cm^3^) Right/Left	1.34 (0.15)***^b,c^ 0.60 (0.08)/0.73 (0.08)	1.26 (0.20) 0.59 (0.10)/0.67 (0.11)	1.16 (0.14) 0.53 (0.07)/0.62 (0.08)	1.13 (0.13) 0.51 (0.06)/0.62 (0.08)	<0.001	0.23
pmEC^f^ (cm^3^) Right/Left	0.74 (0.09)***^b,c^ 0.35 (0.04)/0.38 (0.05)	0.69 (0.09)*^b^ 0.33 (0.04)/0.35 (0.06)	0.62 (0.07) 0.30 (0.04)/0.32 (0.04)	0.63 (0.07) 0.30 (0.04)/0.33 (0.05)	<0.001	0.27
BF Ch1-2 nuclei^f^ (cm^3^)	0.11 (0.02)	0.10 (0.03)	0.10 (0.02)	0.10(0.03)	0.138	0.05

Demographic, cognitive, CSF and MRI characteristics. Values are mean (SD) except for gender. *P*-values refer to the main effect across all groups; *p*-values indicate the level of significance *p < 0.05; **p < 0.01; ***p < 0.001; effect sizes were calculated as Cramér’s *V* for the χ^2^ test (gender) and partial eta-squared for one-way and mixed analyses of variance (all other variables).

*^a^Differences compared to the non-AD aMCI group.*

*^b^Differences compared to the AD aMCI group.*

*^c^Differences compared to the mild AD dementia group.*

*^d^Based on a sample with CSF data (*n* = 60).*

*^e^Based on a sample with complete brain imaging data (*n* = 97).*

*^f^Volume normalized to estimated total intracranial volume. CN, cognitively normal; non-AD aMCI, amnestic mild cognitive impairment with non-Alzheimer’s pathologic change; AD aMCI, amnestic mild cognitive impairment with Alzheimer’s disease; mild AD dementia, mild dementia with Alzheimer’s disease; MMSE, Mini-Mental State Examination; GDS-15, Geriatric Depression Scale 15-item version; BAI, Beck Anxiety Inventory; RAVLT, Rey Auditory Verbal Learning Test; RAVLT 1-5, trials 1–5 total; RAVLT 30, delayed word recall after 30 min; ECR-FR, Enhanced Cued Recall – Free Recall trial; ECR-TR, Enhanced Cued Recall – Total Recall trial; TMT A and B, Trail Making Tests A and B; COWAT, Controlled Oral Word Association Test (Czech version with letters N, K, and P); ROCFT-C, Rey-Osterrieth Complex Figure Test – the Copy condition; ROCFT-R, Rey-Osterrieth Complex Figure Test – the Recall condition after 3 min; DSF, Digit Span Forward total score; DSB, Digit Span Backward total score; CDT, Clock Drawing Test – Cohen’s scoring; SVF, Semantic Verbal Fluency; BNT, Boston Naming Test; CSF, cerebrospinal fluid; *p*-tau_181_, phosphorylated tau_181_; MRI, magnetic resonance imaging; alEC, anterolateral entorhinal cortex; pmEC, posteromedial entorhinal cortex; BF Ch1-2 nuclei, the basal forebrain’s medial septal nuclei and vertical limb of the diagonal band of Broca.*

### Cerebrospinal Fluid Analysis

The CSF samples were obtained by lumbar puncture with an atraumatic needle in the lying position. The first 3 ml of CSF were used for routine analysis and the remaining 10 ml of CSF was centrifuged and stored at –80°C 30 min after the puncture. CSF collection, processing and archiving was performed in accordance with European recommendations (Vanderstichele et al., 2012). CSF amyloid-β_1__–__42_ and p-tau_181_ were analyzed using ELISA (Innogenetics, Ghent, Belgium) in the Cerebrospinal Fluid Laboratory, Institute of Immunology and Department of Neurology, Second Faculty of Medicine, Charles University and Motol University Hospital. Unbiased cut-offs of less than 665 pg/ml and more than 48 pg/ml were used to define amyloid-β_1__–__42_ and *p*-tau_181_ positivity, respectively. These predefined cutoffs (Parizkova et al., 2018) were based on internal receiver operating characteristic (ROC) analyses and were validated against amyloid PET status in the Czech Brain Aging Study with 79% agreement and areas under the ROC curves (AUCs) of 85% (Cerman et al., 2020). The diagnosis of AD was made when both amyloid-β_1__–__42_ and *p*-tau_181_ were positive.

### Magnetic Resonance Imaging Acquisition

We used the established MRI protocol, where the brain scans were performed on a Siemens Avanto 1.5T scanner (Siemens AG, Erlangen, Germany) employing a 12-channel head coil. T1-weighted 3-dimensional high-resolution magnetization-prepared rapid gradient echo (MPRAGE) sequence with the following parameters were used: TR/TE/TI = 2000/3.08/1100 ms, flip angle = 15°, 192 continuous partitions, slice thickness = 1.0 mm and in-plane resolution = 1 mm. Scans were visually inspected to ensure appropriate data quality and to exclude participants with a major brain pathology that could interfere with cognitive functioning. Complete brain imaging data were available for 97 participants.

### Magnetic Resonance Imaging Processing

We used a processing pipeline based on a population-based template and manual segmentation to measure volumes of the hippocampal head, body and tail, and volumes of the alEC and pmEC. The primary T1-weighted sequence was scanned in AC-PC line. Using ITK-SNAP^1^ (Yushkevich et al., 2006) we reconstructed the scans to plane perpendicular to the longitudinal axis of the hippocampus.

#### Template Creation

We used MRI brain scans of 26 CN older adults recruited from the Czech Brain Aging Study (Sheardova et al., 2019) to create a population based template. Following steps were implemented within the freely available Advanced Normalization Tools package (ANTs)^2^. Brain volumes were initially skull-stripped and B1 field intensity inhomogeneity correction was performed using the N4 algorithm (Tustison et al., 2010). All images were registered into MNI space. Then we created an initial registration template using the following parameters: three parallel computations, 1 × 0 × 0 iterations, gradient step of 0.25, and cross-correlation similarity metric. After the initial template was established we proceeded to create definitive template registering images iteratively into the initial template, using 3 parallel computations, gradient step of 0.25, with 30 × 50 × 20 iterations, template construction limit set to 4, with cross-correlation similarity metric.

#### Manual Segmentation of the Hippocampus and the Entorhinal Cortex for Template Creation

Manual segmentation was performed individually for each of 26 CN participants used for template creation. The hippocampus was delineated manually using anatomical landmarks according to the previously published manual segmentation protocol (Berron et al., 2017). Specifically, we delineated three separate parts of the hippocampus - the head, the body and the tail (Supplementary Figure 1). The anterior boundary of the hippocampal head was defined by the posterior boundary of the amygdala, surrounding white matter and the lateral ventricle. The posterior boundary was defined by the last slice before the uncus is separated from the hippocampus. The superior, inferior, medial and lateral boundaries were defined by the temporal horn of the lateral ventricle, the amygdala, the EC and surrounding white matter. The anterior boundary of the hippocampal body was defined by the first slice on the anterior-posterior axis of the hippocampal formation where the uncus has disappeared. The posterior boundary was defined by the last slice where both superior and inferior colliculi were clearly visible. White matter and CSF surrounded the hippocampal body superiorly, inferiorly, medially and laterally. The anterior boundary of the hippocampal tail began one slice posteriorly to the last slice where the colliculi were clearly visible. The posterior boundary was defined by the last slice where the hippocampal tail was clearly visible. White matter and CSF surrounded the hippocampal tail superiorly, inferiorly, medially and laterally.

The EC was delineated manually using anatomical landmarks according to the previously published manual segmentation protocol (Berron et al., 2017; Supplementary Figure 2). Specifically, the segmentation of the EC began four slices anterior to the first slice of the hippocampal head. The EC disappeared after the second slice of the hippocampal body. Because of variability of the collateral sulcus, we used a fixed virtual sagittal plane perpendicular to the coronal plane and passing through the most inferior point of the boundary between gray matter of the EC and adjacent white matter as the lateral boundary of the EC, otherwise we respected anatomical landmarks defined previously (Berron et al., 2017). The EC was divided into the anterolateral and the posteromedial subregions according to the previously published segmentation protocol (Olsen et al., 2017; Supplementary Figure 2). On the first six slices of the EC only the alEC was present. The pmEC began on the seventh slice of the EC. The alEC and the pmEC had the same extent on the slice that was approximately in two thirds of the length of the hippocampal head. Posteriorly from this point the pmEC gradually enlarged. The last slice where the uncus was present (i.e., the last slice of the hippocampal head) was the last slice where the aIERC was visible. Finally, on the last two slices of the EC (i.e., the first two slices of the hippocampal body) only the pmEC was present.

All manually delineated ROIs were then normalized to MNI space using deformation fields obtained during the template creation. We created individual templates of each structure (i.e., the hippocampal head, body and tail, alEC and pmEC) using the same procedure and parameters as described in template creation section. Resulting masks were then rescaled into values 0–100 to represent probabilistic distribution.

#### Segmentation of the Hippocampus and the Entorhinal Cortex

The following steps were implemented to measure individual volumes of hippocampal and EC subregions. We skull-stripped the individual MRI scans, performed B1 field intensity inhomogeneity correction using N4 algorithm and performed three-tissue segmentation using statistical parametric mapping (SPM8, Wellcome Trust Center for Neuroimaging) and the VBM8-toolbox^3^ implemented in MatLab R2015b (MathWorks, Natick, MA, United States). Then we registered the previously created Czech Brain Aging Study template and diffeomorphically warped it into individual participants’ space using ANTs, with cross-correlation method, 100 × 100 × 50 iterations and symmetric normalization applied on 0.25 threshold. The resulting warp field was used to transform ROI masks of individual structures into the participants’ space. ROIs masks were subsequently masked with gray matter ROI and their volumes were extracted. Warps were visually inspected for accuracy, no volumes were removed. Volumes were normalized to eTIV using the previously published regression formula (Jack et al., 1992). Left and right volumes of the hippocampal and EC subregions were summed into a single total volume for each subregion.

#### Basal Forebrain Segmentation

We used the same preprocessing procedures as described above (i.e., skull stripping and B1 field intensity inhomogeneity correction) and followed the previously published protocol to measure volumes of the BF nuclei (Teipel et al., 2005, 2014; Wolf et al., 2014). MRI data were processed using SPM8 and VBM8-toolbox implemented in MatLab R2015b. As in the previous studies (Parizkova et al., 2018, 2020), we used the BF mask based on a cytoarchitectonic map of the BF cholinergic nuclei aligned in MNI space, derived from combined histology and MRI of a postmortem brain. Location of the BF nuclei was identified using histological staining, manually transferred into postmortem MRI space and subsequently transformed into MNI standard space (Teipel et al., 2005; Kilimann et al., 2014). The mask included BF subregions corresponding to the Ch1-2, Ch3, Ch4p (posterior), Ch4ai (anterior and intermediate) nuclei and nucleus subputaminalis. We non-linearly registered all the images into the MNI152 template and used the resulting DARTEL parameters (Ashburner, 2007) to warp the cytoarchitectonic map into individual brain scans. Volumes of the BF Ch1-2 nuclei (Mesulam et al., 1983b) were extracted. The warps were visually assessed for accuracy, no volumes were removed. BF Ch1-2 nuclei volumes were normalized to eTIV using the previously published regression formula (Jack et al., 1992). Left and right Ch1-2 nuclei volumes were summed into a single measure of total Ch1-2 nuclei volume. Group-wise MRI characteristics are listed in Table 1.

### Data Analysis

A one-way analysis of variance (ANOVA) with *post hoc* Sidak’s test was used for continuous variables. A χ^2^ test was used for changes in proportion (gender). The 4 × 4 mixed factorial ANOVA with diagnostic group (CN vs. non-AD aMCI vs. AD aMCI vs. mild AD dementia) as the between-subjects factor and spatial separation (0 vs. 0.5 vs. 1.0 vs. 1.5 cm) as the within-subjects factor was used to analyze accuracy of spatial pattern separation performance measured as the percentage of correct responses, which was the dependent variable. The Greenhouse–Geisser correction was used to correct for violation of sphericity. The *post hoc* planned polynomial contrasts were used to assess the effect of spatial separation in the whole sample. The *post hoc* Sidak’s test was used to compare average differences in spatial pattern separation performance between individual groups. The *post hoc* pairwise comparisons with Holm–Bonferroni correction for multiple comparisons were used to compare differences in spatial pattern separation performance between individual groups for each spatial separation and to interpret the significant interactions between variables. A one-sample *t*-test was used to assess differences from chance performance (i.e., 50%) for each diagnostic group in the task overall and at each spatial separation. Next, the 4 × 4 mixed factorial ANCOVA with age, gender, and years of education sequentially entered as covariates was conducted to address the possibility that differences between the diagnostic groups in the spatial pattern separation task may be influenced by demographic factors. The ROC analysis was used to assess the ability of the spatial pattern separation task to differentiate the CN, non-AD aMCI, AD aMCI, and mild AD dementia groups. Sizes of the AUCs, sensitivity, specificity and optimal cut-off values based on the Youden’s index were calculated.

**FIGURE 2:**
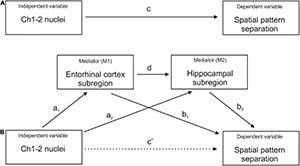
Mediation analysis. **(A)** The total effect represents the association between Ch1-2 nuclei and spatial pattern separation performance without the mediators (c path). **(B)** The direct effect represents the association between Ch1-2 nuclei and spatial pattern separation performance accounting for the mediators (c’ path). The indirect effect represents the association between Ch1-2 nuclei and spatial pattern separation performance through the mediators (a1*b1, a2*b2, and a1*d*b2 paths). The indirect effect includes the associations between Ch1-2 nuclei and specific entorhinal cortex and hippocampal subregions (a1 and a2 paths), the associations between specific entorhinal cortex and hippocampal subregions and spatial pattern separation performance (b1 and b2 paths) and the association between the entorhinal cortex subregion and the hippocampal subregion (d path). Each entorhinal cortex and hippocampal subregion that was significant in the regression analysis was included separately as the mediator.

The Pearson’s correlation coefficients were calculated to explore the bivariate relationships between volumes of the BF Ch1-2 nuclei, hippocampal and EC subregions and spatial pattern separation performance. Holm–Bonferroni correction for multiple comparisons was used in the correlation analysis. Next, the linear regression models adjusted for age, gender and years of education were used to control for the effect of demographic characteristics on the significant associations. Mediation (path) analyses were conducted to assess the association between BF Ch1-2 nuclei volume (the independent variable) and spatial pattern separation performance (the dependent variable) with EC and hippocampal subregions, which were significant in the previous regression analyses, serving as the mediators operating in serial (M1 and M2, respectively). These analyses were adjusted for age, gender and education. The bootstrapping method (Hayes, 2013) was used to test for significance of the indirect effect with a 95% confidence interval (CI). In the mediation analyses (Figure 2), the “a_1_ and a_2_ paths” represent the relationships between the independent variable and the first (M1) and the second (M2) mediator, respectively, the “b_1_ and b_2_ paths” represent the relationships between the M1 and the M2, respectively, and the dependent variable, the “d path” represents the relationship between the M1 and the M2, the “c path” (total effect) represents the effect of the independent variable on the dependent variable, and the “c’ path” (direct effect) represents the effect of the independent variable on the dependent variable while accounting for the mediators.

Statistical significance was set at two-tailed (alpha) of 0.05. Effect sizes are reported using partial eta-squared (η_p_^2^) for mixed factorial ANOVA and ANCOVA. Partial eta-squared of 0.2 corresponds to Cohen’s *d* of 1.0. All analyses were conducted using IBM SPSS for Windows version 25.0.

## Results

### Demographics and Cognitive Performance

The demographic characteristics are presented in detail in Table 1. The groups did not differ in age. The CN group was more educated than the mild AD dementia group (*p* = 0.007). There were more women in the CN and mild AD dementia groups than in the non-AD aMCI and AD aMCI groups (79 and 73% vs. 46 and 51%). As expected, the non-AD aMCI, AD aMCI and mild AD dementia groups had lower MMSE scores (*p* < 0.001) and lower cognitive performance especially in memory and language tests (*p* ≤ 0.026) compared to the CN group. The non-AD aMCI and AD aMCI groups did not differ in cognitive performance. The non-AD aMCI and mild AD dementia group reported higher level of depressive symptoms than the CN group (*p* < 0.001). There were no differences in the level of anxiety symptoms between the groups.

### Spatial Pattern Separation Performance

**FIGURE 3:**
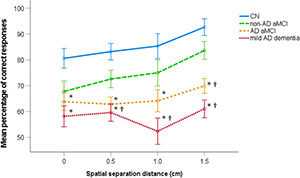
Spatial pattern separation performance. Mean percentage of correct performance for each spatial separation (±1 SE). ^∗^*p* < 0.05 compared to the CN group; †*p* < 0.05 compared to the non-AD aMCI group. CN, cognitively normal; non-AD aMCI, amnestic mild cognitive impairment with non-Alzheimer’s pathologic change; AD aMCI, amnestic mild cognitive impairment with Alzheimer’s disease; mild AD dementia, mild dementia with Alzheimer’s disease.

The mean percentage of correct performance for each spatial separation in the CN, non-AD aMCI, AD aMCI, and mild AD dementia groups are presented in Figure 3. In the 4 (diagnostic group) × 4 (spatial separation) mixed factorial ANOVA, there was a significant main effect of diagnostic group (*F*[3,114] = 22.29, *p* < 0.001, η_p_^2^ = 0.37). On average, the AD aMCI group had less accurate spatial pattern separation performance than the non-AD aMCI group (*p* = 0.039, 95% CI [–18.80, –0.31]) and the CN group (*p* < 0.001, 95% CI [–29.21, –11.29]), and did not differ from the mild AD dementia group (*p* = 0.190, 95% CI [–1.86, 16.64]). The non-AD aMCI group had less accurate performance than the CN group (*p* = 0.024, 95% CI [–20.45, –0.94]) and more accurate performance than the mild AD dementia group (*p* < 0.001, 95% CI [6.93, 26.97]). Specifically, the AD aMCI group had less accurate performance than the non-AD aMCI group at the 1.5 cm spatial separation [*t*(62) = 3.06, *p* = 0.008] and the CN group at each spatial separation [*t*(65) ≥ 3.28, *p* ≤ 0.007]. The AD aMCI group did not differ from the mild AD dementia group at any spatial separation [*t*(62) ≤ 1.98, *p* ≥ 0.101]. The non-AD aMCI group had less accurate performance than the mild AD dementia group at the 0.5, 1.0, and 1.5 cm spatial separations [*t*(51) ≥ 2.75, *p* ≤ 0.028] and did not differ from the CN group at any spatial separation [*t*(65) ≤ 2.30, *p* ≥ 0.070]. Further, there was a significant main effect of spatial separation (*F*[1.91,218.01] = 6.05, *p* = 0.003, η_p_^2^ = 0.05). Specifically, there was a significant linear effect of spatial separation (*F*[1,114] = 11.88, *p* = 0.001, η_p_^2^ = 0.094) where, on average, as the distance in spatial separation increased, the performance improved. The spatial separation-by-diagnostic group interaction was not significant (*F*[5.74,218.01] = 0.79, *p* = 0.571, η_p_^2^ = 0.02). The CN, non-AD aMCI, and AD aMCI groups performed above the chance level in the task overall and at each spatial separation (CN group: [*t*(28) ≥ 9.75, *p* < 0.001]; non-AD aMCI group: [*t*(25) ≥ 4.39, *p* < 0.001]; AD aMCI group: [*t*(36) ≥ 2.99, *p* ≤ 0.005]). The mild AD dementia group performed above the chance level in the task overall and at 0.5 and 1.5 cm spatial separations [*t*(25) ≥ 2.39, *p* ≤ 0.025], while performance in this group at 0.0 and 1.0 spatial separations did not differ from the chance level [*t*(25) ≤ 1.72, *p* ≥ 0.098].

To address the possibility that differences between the diagnostic groups in the spatial pattern separation task may be influenced by demographic factors, age, gender and years of education were sequentially entered into the model as covariates. Again, a significant main effect of diagnostic group retained (*F*[3,113] = 21.57, *p* < 0.001, η_p_^2^ = 0.36). The AD aMCI group still had less accurate spatial pattern separation performance than the non-AD aMCI group (*p* < 0.050, 95% CI [–18.48,–0.01]) and the CN group (*p* < 0.001, 95% CI [–28.38, –10.30]) and did not differ from the mild AD dementia group (*p* = 0.183, 95% CI [–1.79, 16.64]). The non-AD aMCI group still had more accurate performance than the mild AD dementia group (*p* < 0.001, 95% CI [5.25, 25.23]) but the difference between the non-AD aMCI and CN groups became non-significant (*p* = 0.080, 95% CI [–19.29, 0.66]). The main effect of spatial separation was not significant (*F*[1.90, 214.84] = 2.01, *p* = 0.139, η_p_^2^ = 0.02) and the effect of the spatial separation-by-diagnostic group interaction remained non-significant (*F*[5.70, 214.84] = 0.77, *p* = 0.589, η_p_^2^ = 0.02).

In the ROC analyses, spatial pattern separation performance differentiated the CN group from the non-AD aMCI, AD aMCI and mild AD dementia groups with AUC values of 0.76 (95% CI [0.63, 0.89], *p* = 0.001), 0.88 (95% CI [0.81, 0.96], *p* < 0.001), and 0.94 (95% CI [0.87, 1.00], *p* < 0.001), respectively, the non-AD aMCI group from the AD aMCI and mild AD dementia groups with AUC values of 0.67 (95% CI [0.53, 0.80], *p* = 0.024) and 0.79 (95% CI [0.67, 0.92], *p* < 0.001), respectively, and did not differentiate the AD aMCI group from the mild AD dementia group, where the AUC value was 0.63 (95% CI [0.49, 0.77], *p* = 0.083). The sensitivity and specificity for the relevant cut-off values is listed in Table 2.

**TABLE 2:** Sensitivity and specificity for the relevant cut-off values in the spatial pattern separation task.

	**Cut-off value^a^ (%)**	**Sensitivity/Specificity^a^ (%)**
CN vs. non-AD aMCI	79	69/69
CN vs. AD aMCI	76	86/76
CN vs. mild AD dementia	70	100/77
Non-AD aMCI vs. AD aMCI	64	82/44
Non-AD aMCI vs. mild AD dementia	67	73/73
AD aMCI vs. mild AD dementia	NA	NA

*^a^Based on the Youden’s index.*

*CN, cognitively normal; non-AD aMCI, amnestic mild cognitive impairment with non-Alzheimer’s pathologic change; AD aMCI, amnestic mild cognitive impairment with Alzheimer’s disease; mild AD dementia, mild dementia with Alzheimer’s disease.*

### Volumes of the Hippocampal and Entorhinal Cortex Subregions and the Ch1-2 Nuclei and Spatial Pattern Separation Performance

The MRI characteristics are presented in detail in Table 1. The between-group differences were significant for volumes of the hippocampal head, body and tail, alEC and pmEC (*F*[3,100] ≥ 4.59, *p* ≤ 0.005, η_p_^2^ ≥ 0.12), where the CN group had higher volumes than the AD aMCI and mild AD dementia groups (*p* ≤ 0.032) and was similar to the non-AD aMCI group (*p* ≥ 0.200). The AD aMCI had smaller volumes of the hippocampal tail and the pmEC than the non-AD aMCI group (*p* = 0.009 and *p* = 0.024, respectively). In the correlational analyses (Table 3), volumes of the hippocampal tail and body, pmEC and BFCh1-2 nuclei correlated with spatial pattern separation performance (*r* ≥ 0.28, *p* ≤ 0.006). These associations remained significant in the regression analyses adjusted for age, gender and education, where decreased volumes were related to less accurate performance (β ≥ 0.26, *p* ≤ 0.017) (Table 4).

**TABLE 3:** Correlation matrix of spatial pattern separation performance and volumes of specific hippocampal and entorhinal cortex subregions and basal forebrain Ch1-2 nuclei.

	**1**	**2**	**3**	**4**	**5**	**6**	**7**
(1) Spatial pattern separation	–						
(2) Hippocampal head	0.195	–					
(3) Hippocampal body	0.277**	0.436***	–				
(4) Hippocampal tail	0.312**	0.312**	0.743***	–			
(5) alEC	0.200*	0.515***	0.494***	0.390***	–		
(6) pmEC	0.299**	0.421***	0.364***	0.285**	0.784***	–	
(7) BF Ch1-2 nuclei	0.279**	0.131	0.302**	0.311**	0.207*	0.260*	–

*Correlation is significant at the 0.05 level (two-tailed), **Correlation is significant at the 0.01 level (two-tailed), ***Correlation is significant at the 0.001 level (two-tailed). Values in bold are significant after Holm–Bonferroni correction for multiple comparisons.

*alEC, anterolateral entorhinal cortex; pmEC, posteromedial entorhinal cortex; BF Ch1-2 nuclei, the basal forebrain’s medial septal nuclei and vertical limb of the diagonal band of Broca.*

**TABLE 4:** Regression analyses of spatial pattern separation performance and volumes of specific hippocampal and entorhinal cortex subregions and basal forebrain Ch1-2 nuclei controlled for demographic characteristics.

	**Pattern separation (% correct)**
	β
*Model 1*	
Hippocampal body^a^ (cm^3^)	0.265**
Age (y)	–0.126
Gender (male = 0)	0.120
Education (y)	0.254**
*Model 2*	
Hippocampal tail^a^ (cm^3^)	0.305**
Age (y)	–0.147
Gender (male = 0)	0.121
Education (y)	0.251**
*Model 3*	
pmEC^a^ (mm^3^)	0.260*
Age (y)	–0.124
Gender (male = 0)	0.104
Education (y)	0.233*
*Model 4*	
BF Ch1-2 nuclei^a^ (mm^3^)	0.258*
Age (y)	0.260
Gender (male = 0)	0.136
Education (y)	0.231*

*p < 0.05; **p < 0.01; and ***p < 0.001.

*^*a*^Volume normalized to estimated total intracranial volume.*

*β, standardized regression coefficient; pmEC, posteromedial entorhinal cortex; BF Ch1-2 nuclei, the basal forebrain’s medial septal nuclei and vertical limb of the diagonal band of Broca.*

Based on the results of the regression analyses, three mediators (hippocampal tail and body and pmEC volumes) were used in the mediation analyses resulting in two mediation models. The first mediation model included pmEC and hippocampal tail volumes as mediators operating in serial and the second mediation model included pmEC and hippocampal body volumes as mediators operating in serial. The total effect for the association between BF Ch1-2 nuclei volume and spatial pattern separation performance was significant in both models (total effect: 95% CI [0.032, 0.321], *p* = 0.017). In the first mediation model, the association between BF Ch1-2 nuclei volume and spatial pattern separation performance was mediated by pmEC and hippocampal tail volumes (total indirect effect: 95% CI [0.024, 0.152]). Specifically, two indirect paths were significant in the model; the path with hippocampal tail volume serving as a single mediator (indirect effect: 95% CI [0.001, 0.108]) and the path with pmEC and hippocampal tail volumes serving as mediators operating in serial (indirect effect: 95% CI [0.000, 0.028]). The indirect path with pmEC volume serving as a single mediator was not significant (indirect effect: 95% CI [–0.001, 0.067]). The direct effect of BF Ch1-2 nuclei volume on spatial pattern separation performance was not significant (direct effect: 95% CI [–0.037, 0.252], *p* = 0.144) indicating that pmEC and hippocampal tail volumes fully mediated the association between BFCh1-2 nuclei volume and spatial pattern separation performance.

In the second mediation model, the association between BF Ch1-2 nuclei volume and spatial pattern separation performance was mediated by pmEC and hippocampal body volumes (total indirect effect: 95% CI [0.012, 0.115]). Specifically, the indirect path with pmEC and hippocampal body volumes serving as mediators operating in serial was significant (indirect effect: 95% CI [0.000, 0.028]). The indirect paths with pmEC and hippocampal body volumes serving as single mediators were not significant (indirect effects: 95% CI [–0.001, 0.071] and 95% CI [–0.003, 0.079], respectively). The direct effect of BF Ch1-2 nuclei volume on spatial pattern separation performance was not significant (direct effect: 95% CI [–0.020, 0.269], *p* = 0.090), indicating that pmEC and hippocampal body volumes fully mediated the association between BF Ch1-2 nuclei volume and spatial pattern separation performance.

### Segmentation Metrics

In order to test the reliability of our automatic segmentation protocol, we computed the Sørensen-Dice coefficient (SDC), comparing manual and automatic segmentation of the participants that were used for the template creation. The coefficient is defined as follows: SDC = (2^∗^volume_*overlap*_)/(volume_*automatic*_ + volume_*manual*_). We computed the individual SDC value for each participant and the mean SCD value for each measured region (i.e., hippocampal head, hippocampal body, hippocampal tail, alEC, and pmEC).

The overall average SDC value in the current study was 0.71. For individual structures, the SCD values ranged from 0.62 to 0.85. The most reliable was the segmentation of the hippocampal head (SDC = 0.86), while the least reliable was the segmentation of the pmEC (SDC = 0.62).

## Discussion

We examined the differences in spatial pattern separation performance between AD biomarker positive and negative aMCI participants and explored the associations between the performance and structural measures of specific hippocampal, EC subregions and BF Ch1-2 nuclei, and the interrelations between these regions with respect to the performance. We found that the AD aMCI participants had less accurate spatial pattern separation than the non-AD aMCI participants while performing similarly in other cognitive tests. Less accurate spatial pattern separation performance was associated with decreased volumes of the posterior hippocampus, including body and tail, the pmEC and the BF Ch1-2 nuclei. The association between BF Ch1-2 nuclei volume and spatial pattern separation performance was mediated by pmEC and posterior hippocampal volumes. The reliability of the automatic segmentation protocol was comparable to that of commonly used automatic segmentation packages (e.g., Freesurfer) (Schmidt et al., 2018).

This study demonstrated that the aMCI participants with positive AD biomarkers had less accurate spatial pattern separation performance than the aMCI participants with negative AD biomarkers and the CN participants. It is worth noting that AD biomarker positive and negative aMCI participants did not significantly differ in any cognitive test and, after controlling for demographic characteristics, these results remained essentially unchanged, while the differences between AD biomarker negative aMCI and CN participants in spatial pattern separation performance were no longer significant. Our results support the previous findings that indicated the potential of the spatial pattern separation task to differentiate older adults with biomarker-defined early AD and CN older adults (Parizkova et al., 2020) and further extend them by showing that the task could differentiate AD biomarker positive and negative aMCI participants with high diagnostic sensitivity (>80%). These results are consistent with recent studies showing that spatial memory testing in real-space and virtual environments can differentiate amyloid-β positive and negative patients with aMCI (Schöberl et al., 2020) and AD biomarker positive and negative older adults with MCI (Howett et al., 2019), respectively. They are also consistent with previous work indicating that higher cortical amyloid-β accumulation in older adults may be associated with worse performance in a scene discrimination task with two levels of interference (Maass et al., 2019) and a task combining spatial and object mnemonic discrimination (Webb et al., 2020). Our task unlike other mnemonic discrimination tasks evaluates selectively spatial pattern separation in four levels of increasing spatial interference. This is an important core feature of the task because previous research suggested that object pattern separation is affected in normal aging and thus may not be specific for early AD (Reagh et al., 2016). Therefore, this pure spatial pattern separation task has the potential to improve the early detection of cognitive deficits associated with AD. This task may complement standardized cognitive assessment with traditional neuropsychological tests that are used to identify patients with MCI and dementia but do not achieve sufficient diagnostic sensitivity to differentiate cognitive deficit in early AD and other neurodegenerative diseases (Flanagan et al., 2016; Coughlan et al., 2018). This could be of great importance as PET imaging, CSF and blood-based biomarkers used to detect the early stages of AD are currently limited to research settings and expert clinics. Further research is needed to clarify how early spatial pattern separation deficits emerge in the course of AD, especially in preclinical AD, and whether they are caused directly by accumulation of amyloid-β, spread of the tau pathology from the PrC and alEC to the pmEC and posterior-medial cortical regions, which is facilitated by amyloid-β deposition (Jacobs et al., 2018), or by combination of both pathologies. We replicated the findings from our previous study (Parizkova et al., 2020) showing that task performance declines with increasing spatial interference (i.e., smaller distance between the target and foil circles) across the groups. Our current results thus confirm that performance in the task involving varying degrees of spatial interference reflects spatial pattern separation, which is the memory process important for encoding and subsequent recall of locations that share similar contextual features (Gilbert and Kesner, 2006).

The current version of the spatial pattern separation task was designed to directly assess hippocampal function by including a time delay of 20 s with a distraction task between the sample and the choice phases. Previous research (Kesner and Hopkins, 2006) using a similar spatial pattern separation task with delays of 5, 10, 20, and 30 s between the encoding and the recall phase showed that participants with hippocampal atrophy due to hypoxia were not able to discriminate spatial separation distances when the delay was 10 s or higher, while they managed to perform discrimination comparably to the control group when the delay was 5 s. These results suggest that longer time delays (i.e., 10 s or longer) depend on the function of the hippocampus while shorter time delays (i.e., 5 s) are not hippocampus dependent. This was also supported by our previous study (Parizkova et al., 2020), where spatial pattern separation performance after 10 and 20 s delay was associated with hippocampal volume in individuals with AD. Next, in our current study we used a distraction task, where the participants read aloud a randomly appearing string of numbers between the sample and the choice phases. It should be noted that a distraction task during the time delay may induce forgetting on delayed-match-to sample tasks by interfering with online maintenance of the target location, which was reported in individuals with hippocampal damage (Race et al., 2013).

In this study, we measured volumes of the hippocampal and EC subregions, chosen for their role in pattern separation, to explore the structural brain alterations that may underlie differences in performance in the spatial pattern separation task. The AD aMCI participants had smaller volumes in all hippocampal and EC subregions (i.e., hippocampal head, body and tail, alEC and pmEC) compared to the CN participants and in the hippocampal tail and the pmEC compared to the non-AD aMCI participants. These findings are in line with previous research demonstrating MTL atrophy in early AD (Velayudhan et al., 2013; Spampinato et al., 2016; Parizkova et al., 2020), especially previous work showing more pronounced AD-related atrophy in the posterior subregions (Lindberg et al., 2017; Lladó et al., 2018). The findings of the non-significant differences between the AD aMCI and non-AD MCI participants in volumes of the alEC and the anterior hippocampus, the earliest sites where tau neurofibrillary tangles emerge (Braak and Braak, 1991), may indicate similar structural changes in these MTL subregions in early AD and other tau-related neurodegenerative diseases (Josephs et al., 2017). Consistent with our hypothesis, less accurate spatial pattern separation performance was associated with reduced volumes of the posterior hippocampus, including body and tail, and the pmEC but not with anterior hippocampal (i.e., head) and alEC volumes. To the best of our knowledge, this is the first demonstration that spatial pattern separation deficits are selectively associated with decreased posterior hippocampal and pmEC volumes within the hippocampal and EC subregions. These findings complement our previous work that showed a relationship between hippocampal and EC atrophy and spatial pattern separation impairment (Parizkova et al., 2020). They also extend previous research that reported the associations between functional alterations in the posterior hippocampus (Lee et al., 2008) and the pmEC (Berron et al., 2018) and performance in spatial discrimination tasks. Collectively, these findings shed further light on the functional differentiation of the EC into the alEC and pmEC subregions (Maass et al., 2015; Navarro Schröder et al., 2015) and on the functional differentiation along the anterior-posterior longitudinal axis of the hippocampus (Pihlajamäki et al., 2004; Nadel et al., 2013) in humans.

We measured volume of the BF Ch1-2 nuclei, chosen as the main origin of cholinergic projections to the hippocampus and the EC, to explore their structural alterations and specific pathways that may be associated with performance in the spatial pattern separation task. Decreased BF Ch1-2 nuclei volume was associated with less accurate spatial pattern separation performance and reduced volumes of the hippocampal and EC subregions, especially the posterior hippocampus, including body and tail, and the pmEC. This is consistent with our previous research showing that decreased BF Ch1-2 nuclei volume is associated with less effective performance in spatial pattern separation (Parizkova et al., 2020) and spatial memory (Parizkova et al., 2018) tasks and reduced volumes of the hippocampus (Parizkova et al., 2018, 2020) and the EC (Parizkova et al., 2018, 2020). Additional analyses demonstrated that the association between decreased BF Ch1-2 nuclei volume and less accurate spatial pattern separation performance is fully mediated by reduced volumes of the posterior hippocampus, including body and tail, and the pmEC after controlling for demographic characteristics. Specifically, the analyses revealed three pathways through which the BF Ch1-2 nuclei may be associated with performance in the spatial pattern separation task. In the first pathway, reduced BF Ch1-2 nuclei volume was associated with decreased hippocampal tail volume, which was in turn associated with worse task performance. In the second and the third pathway, reduced BF Ch1-2 nuclei volume was associated with decreased pmEC volume that was further associated with decreased volumes of the hippocampal tail and body, respectively, which were in turn associated with worse task performance. It is worth noting that the pathways linking BF Ch1-2 nuclei volume to task performance directly and indirectly through pmEC volume were not significant.

In contrast to our previous research (Parizkova et al., 2020) that suggested the direct association between BF Ch1-2 nuclei volume and spatial pattern separation performance, the current findings, consistent with our hypothesis, showed that BF Ch1-2 nuclei volume is indirectly associated with task performance through hippocampal tail volume and through volumes of the pmEC and the posterior hippocampus, respectively. It should be noted that in the previous study (Parizkova et al., 2020), we did not analyze the indirect pathway linking BF Ch1-2 nuclei volume to spatial pattern separation performance through EC volume and we measured total hippocampal volume that combined anterior (i.e., head) and posterior (i.e., body and tail) volumes. In the current study, anterior hippocampal volume was not associated with spatial pattern separation performance. This may explain our previous findings of the non-significant indirect pathway linking BF Ch1-2 nuclei volume to task performance through total hippocampal volume and emergence of the significant direct pathway. Importantly, our current findings are consistent with previous animal research that reported specific cholinergic projections from the BF Ch1-2 nuclei to the hippocampus and the EC (Mesulam et al., 1983a; Kondo and Zaborszky, 2016) with more projections to the medial than to the lateral EC (Desikan et al., 2018), representing the rodent homologs of the human pmEC and alEC, respectively, and rodent research that showed less effective spatial pattern separation due to lesions of the BF cholinergic projections to the hippocampus (Ikonen et al., 2002). To the best of our knowledge, this is the first study to demonstrate specific interrelations between the BF Ch1-2 nuclei, the posterior hippocampal and pmEC subregions and spatial pattern separation performance. The current findings reinforce and refine our previous work (Parizkova et al., 2020) that suggested the direct association between decreased hippocampal volume and worse spatial pattern separation performance and the indirect association between reduced EC volume and worse performance that was mediated by decreased hippocampal volume. Collectively, these findings complement and further extend previous animal and human work that demonstrated the essential role of the hippocampus in pattern separation (Yassa and Stark, 2011), especially the role of the dorsal hippocampus in fine spatial discrimination (McTighe et al., 2009), that is strongly modulated by the afferent projections from the EC through the perforant path (Yassa et al., 2011b; Hunsaker and Kesner, 2013) and the BF through the fimbria-fornix pathways (Ikonen et al., 2002). In addition, our findings may help clarify the role of the BF Ch1-2 nuclei and their influence on spatial pattern separation processes through the pmEC and the posterior hippocampus.

One of the strengths of the current study is the fact that this is the first study to date to examine the differences in spatial pattern separation in older adults with aMCI with positive and negative AD biomarkers. In addition, we investigated the complex interrelations between structural measures of the BF Ch1-2 nuclei, the major origin of cholinergic projections to the EC and the hippocampus, and the specific EC and hippocampal subregions and spatial pattern separation that have not been studied in humans. Finally, we used well-defined homogeneous cohorts of CN participants and cognitively impaired older adults, where the diagnosis of AD and non-AD was supported by biomarker assessment including amyloid PET imaging and CSF biomarkers. However, there are several limitations to this study. First, assessment of CSF biomarkers and amyloid PET imaging were not performed in the CN participants to rule out preclinical AD. However, we applied stringent inclusion criteria to minimize this possibility. Second, we did not directly compare spatial and object discrimination performance to assess the extent of impairment in early AD attributed to spatial pattern separation and pattern separation in general. This should be the focus of future studies. Third, the cross-sectional design did not allow evaluating spatial pattern separation performance changes over time but longitudinal follow-up is ongoing.

## Conclusion

In conclusion, the current study demonstrated that AD biomarker positive older adults with aMCI have less accurate spatial pattern separation than those with AD biomarker negative aMCI who scored similarly in other cognitive tests. These findings suggest that spatial pattern separation testing may complement cognitive assessment with traditional neuropsychological tests to help differentiate cognitive deficit in early AD and other neurodegenerative diseases. This is of great importance for early therapeutic interventions in the future. Further, we showed that spatial pattern separation deficits are selectively associated with decreased volumes of the BF Ch1-2 nuclei, the pmEC and the posterior hippocampus. In addition, our results revealed specific interrelations between structural measures of these subregions and spatial pattern separation, where decreased BF Ch1-2 nuclei volume is indirectly associated with worse task performance through reduced hippocampal tail volume and reduced volumes of the pmEC and the posterior hippocampus, respectively. These findings indicate that the BF Ch1-2 nuclei influence spatial pattern separation through the pmEC and the posterior hippocampus, which may provide further insight into the role of the specific BF, EC, and hippocampal subregions in spatial pattern separation processes. The focus of future studies should be to explore the associations between integrity of BF projections and spatial pattern separation performance in early AD and assess the potential of the spatial pattern separation task to detect subtle cognitive changes in preclinical AD.

## Data Availability Statement

The datasets presented in this article are not readily available because of the policy of the Czech Brain Aging Study (CBAS), which allows sharing of the data only after previous approval. Requests to access the datasets should be directed to JL, jan.laczo@lfmotol.cuni.cz.

## Ethics Statement

The studies involving human participants were reviewed and approved by Ethics Committee of Second Faculty of Medicine, Charles University and Motol University Hospital. The participants provided their written informed consent to participate in this study.

## Author Contributions

ML and JL participated in the design of the study, data interpretation, and wrote the draft of the manuscript. OL and LM were involved in MRI data acquisition and processing. JK, HM, MV, and JH were involved in data acquisition and interpretation. All the authors read and approved the final manuscript.

## Conflict of Interest

The authors declare that the research was conducted in the absence of any commercial or financial relationships that could be construed as a potential conflict of interest.

## Publisher’s Note

All claims expressed in this article are solely those of the authors and do not necessarily represent those of their affiliated organizations, or those of the publisher, the editors and the reviewers. Any product that may be evaluated in this article, or claim that may be made by its manufacturer, is not guaranteed or endorsed by the publisher.

## Footnotes

1
www.itksnap.org

2
http://stnava.github.io/ANTs/

3
http://dbm.neuro.uni-jena.de/vbm/
