# Supplementary material for: Spatial Pattern Separation Testing Differentiates Alzheimer’s Disease Biomarker-Positive and Biomarker-Negative Older Adults With Amnestic Mild Cognitive Impairment
Source: Front Aging Neurosci. 2021 Nov 26;13:774600. doi: 10.3389/fnagi.2021.774600 (PMC8662816; doi:10.3389/fnagi.2021.774600)
Supplement: Supplementary file 1 [file Image_1.pdf]

Supplementary Figure 1: Slice-by-slice segmentation of the hippocampus

Hippocampal head

Hippocampal body

Hippocampal tail

HH – Hippocampal head

HB – Hippocampal body

HT – Hippocampal tail

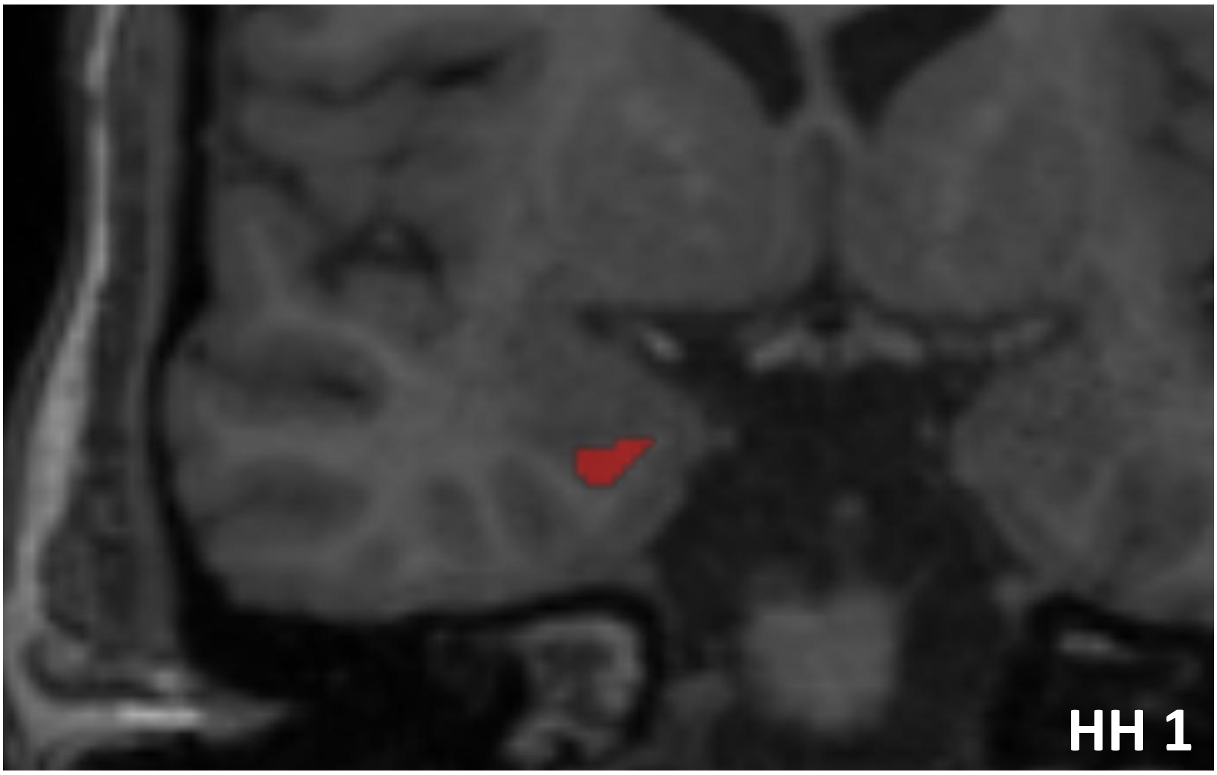

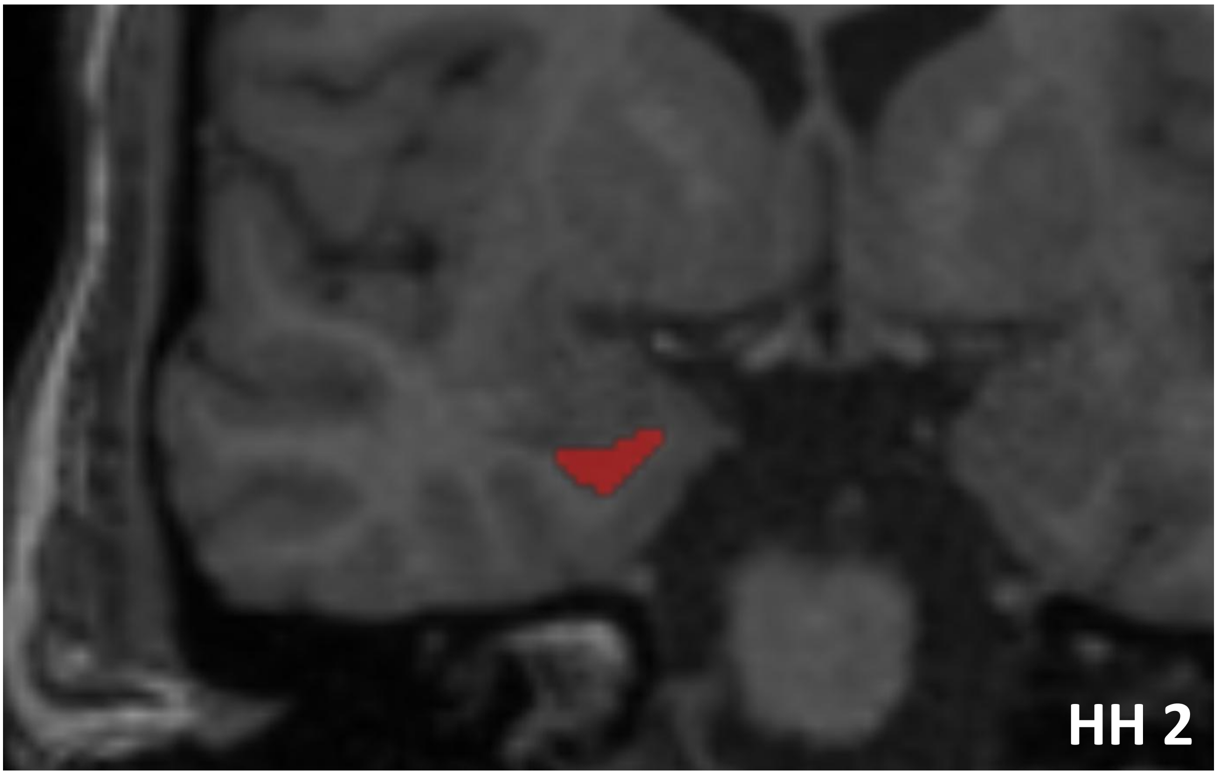
HH 2

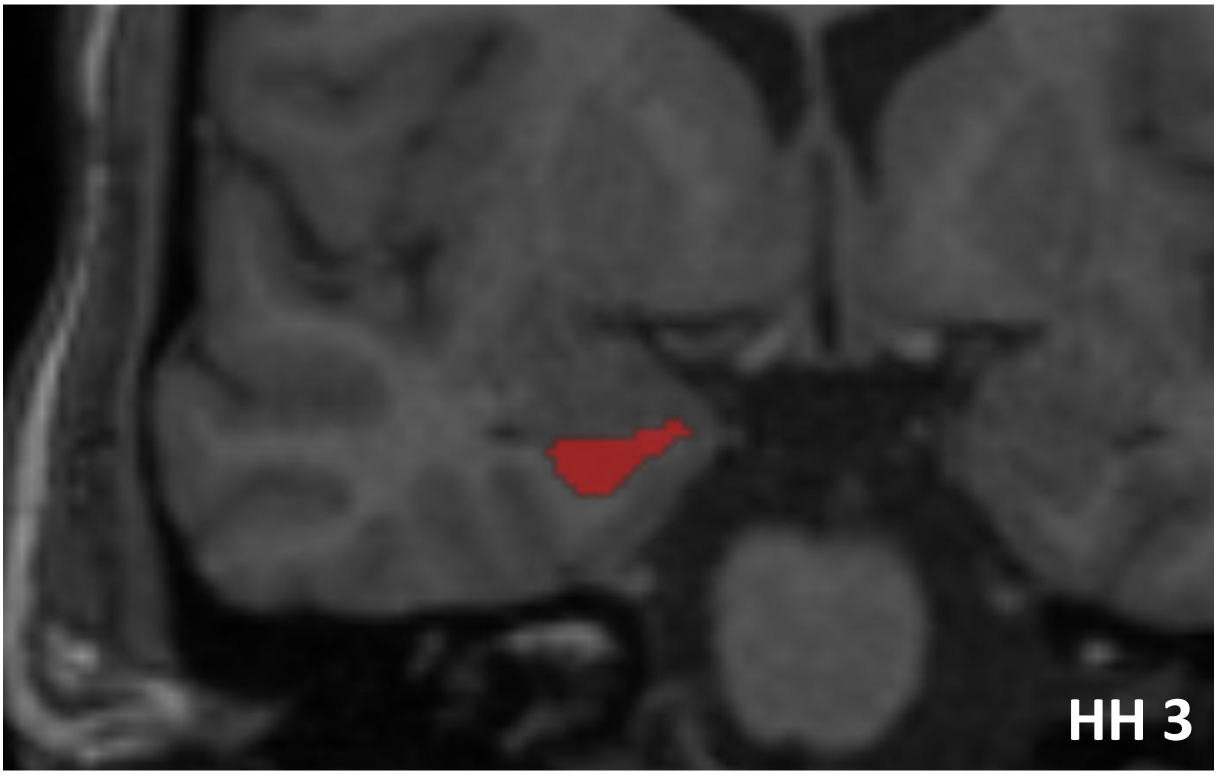
HH 3

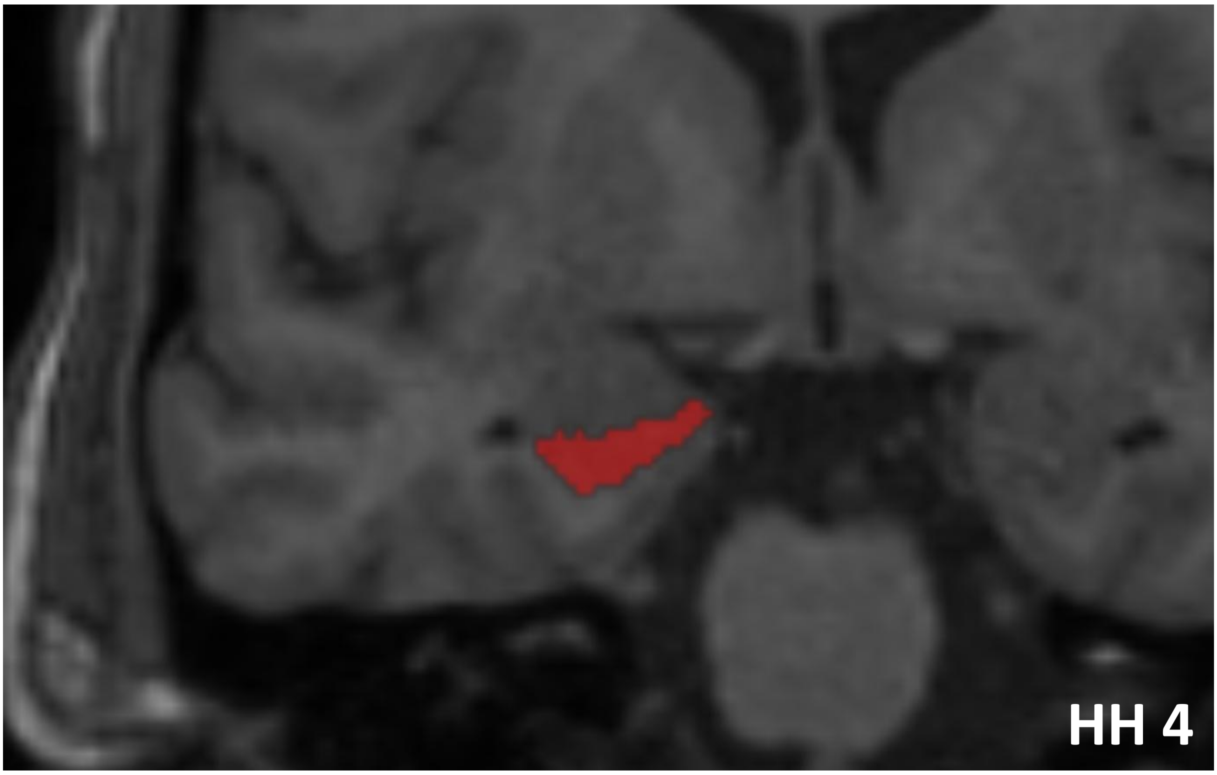
HH 4

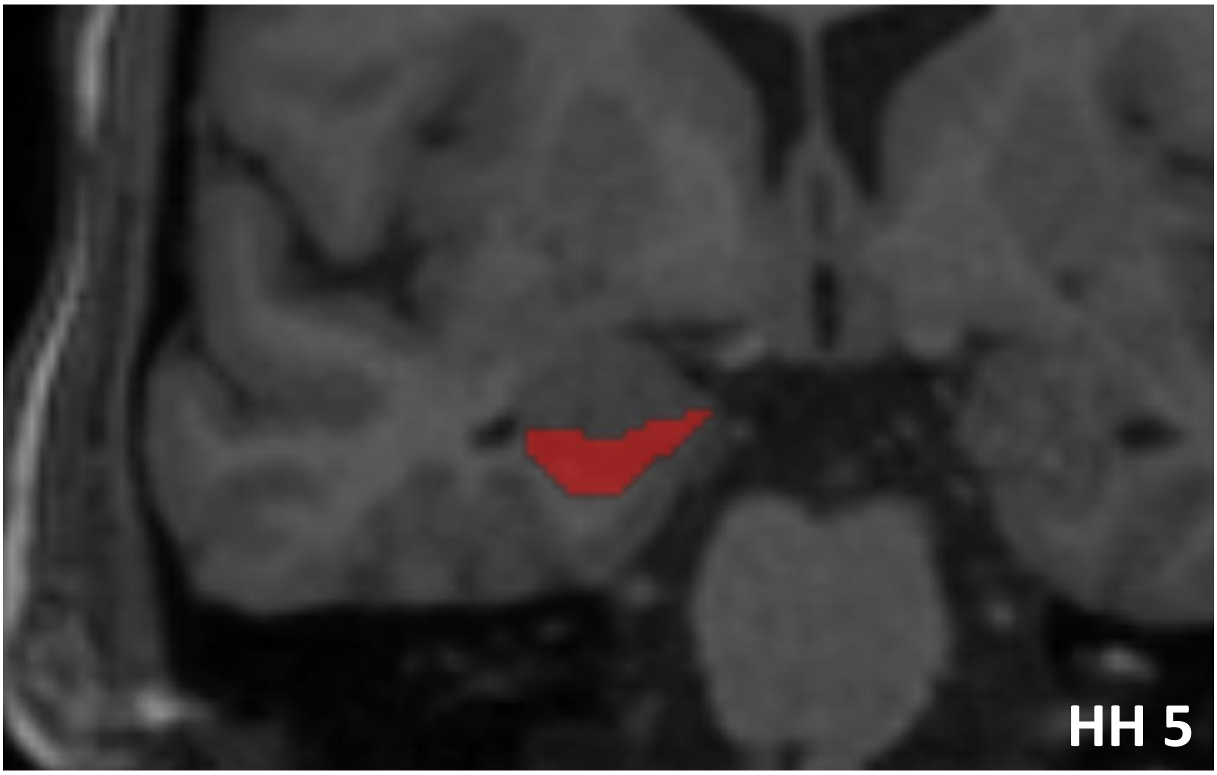
HH 5

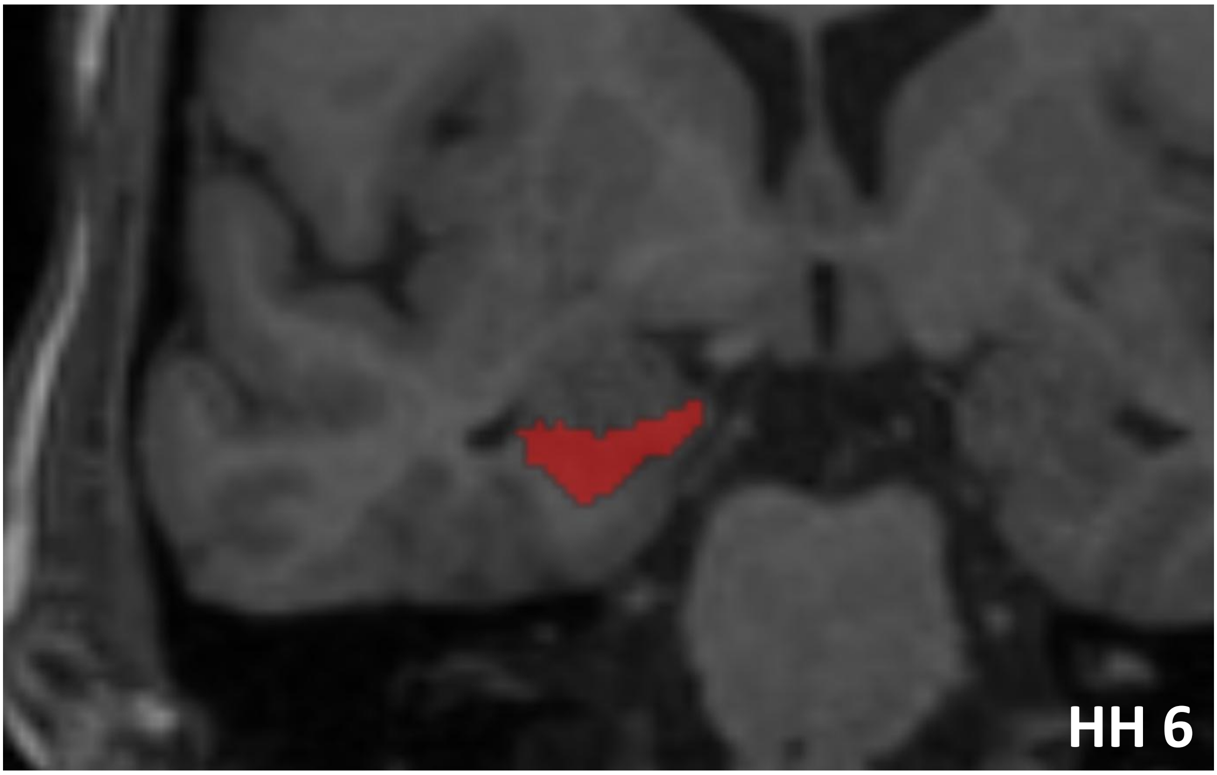
HH 6

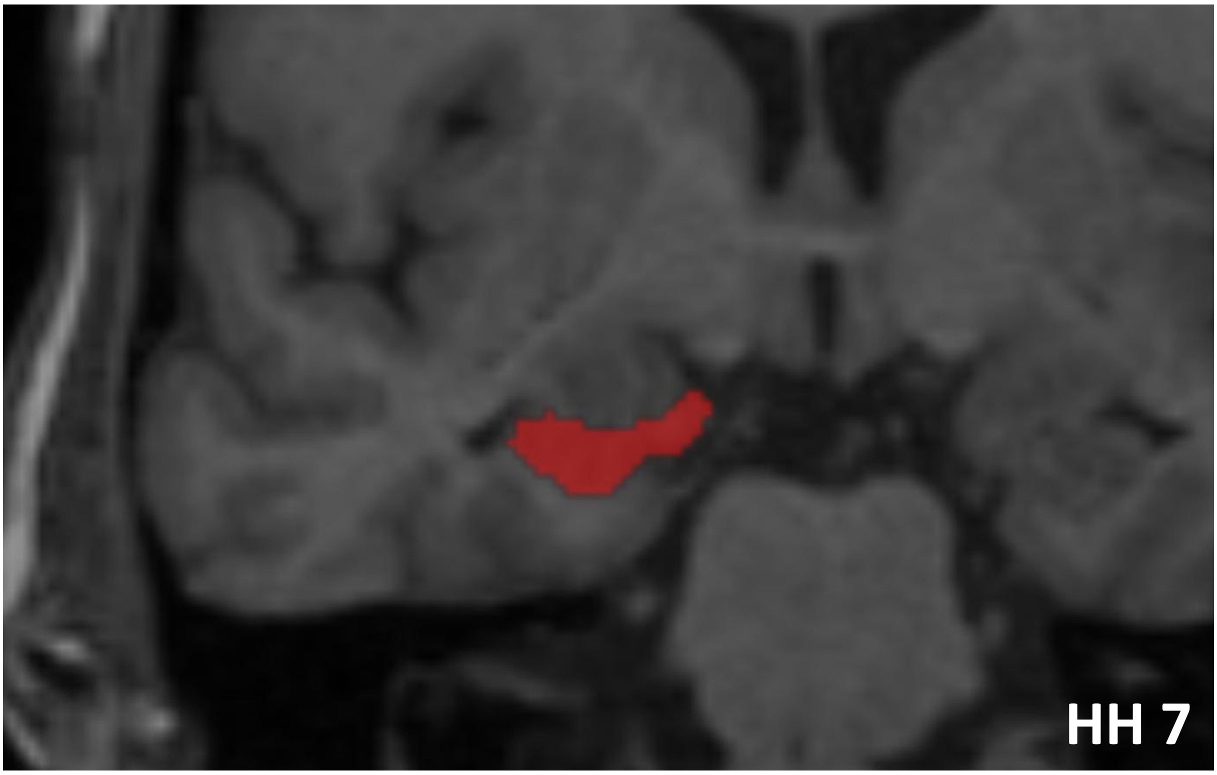
HH 7

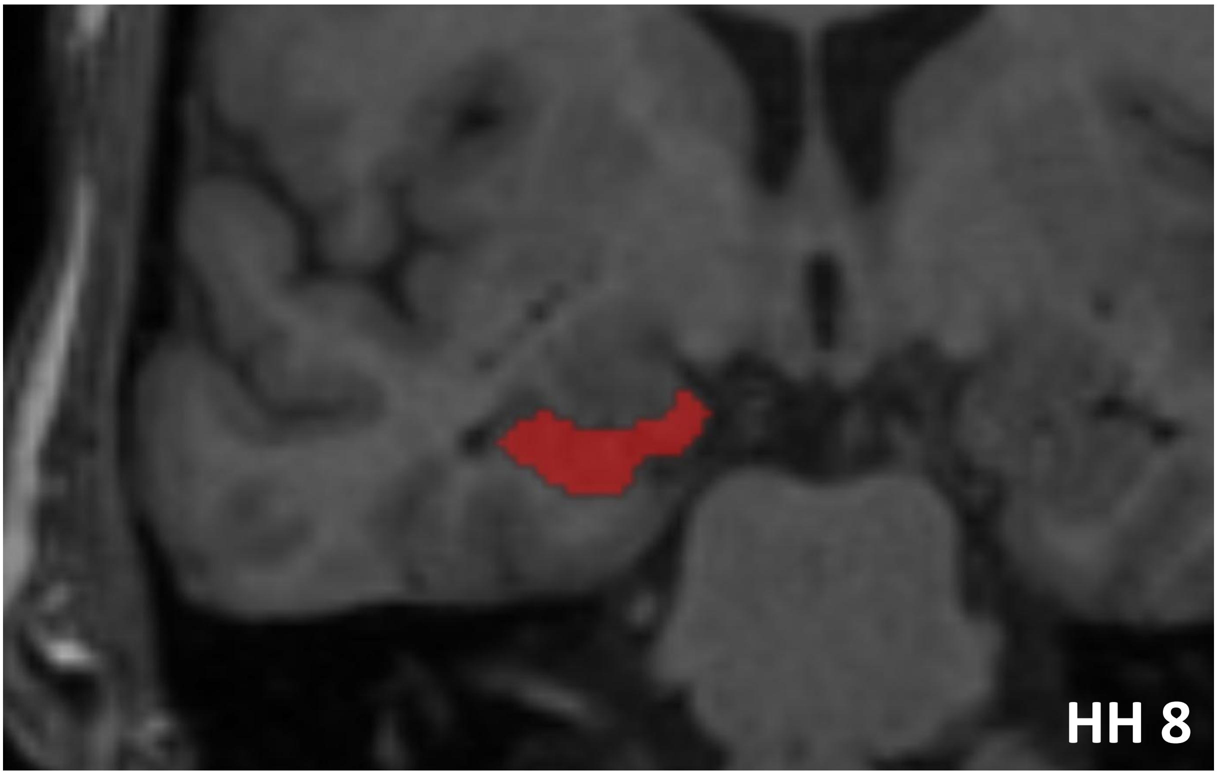
HH 8

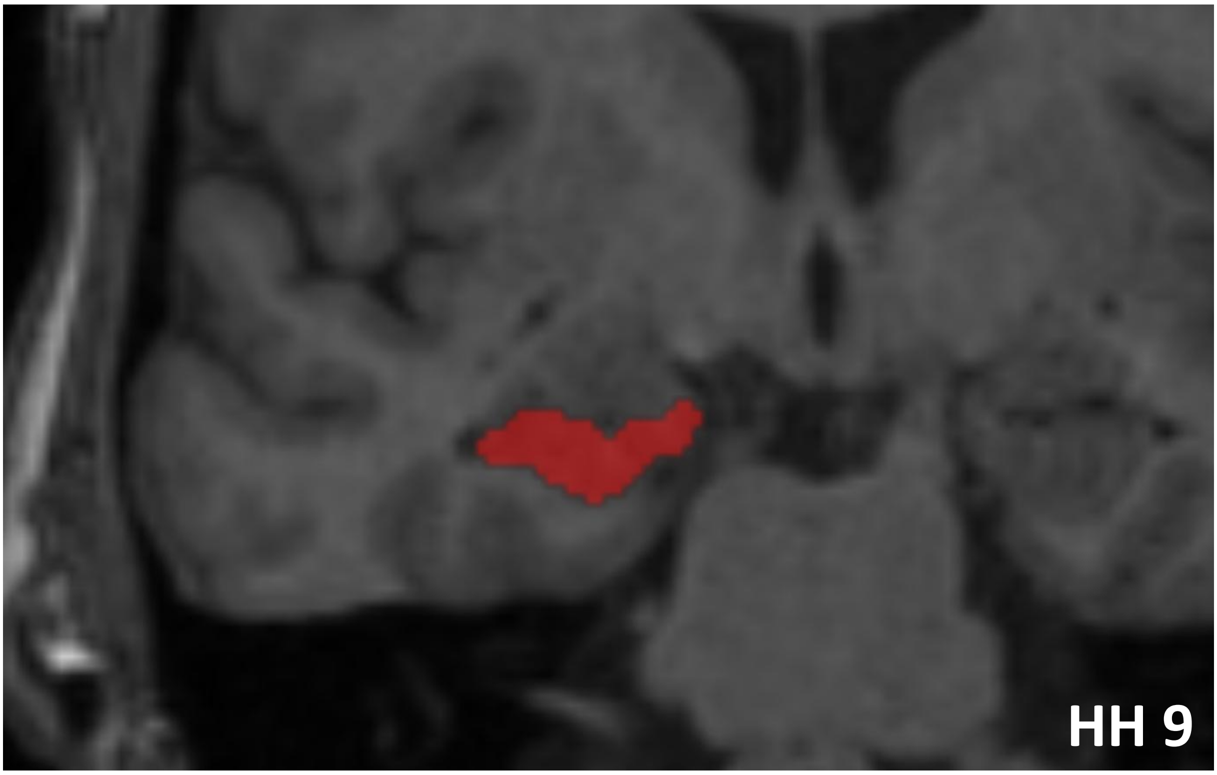
HH 9

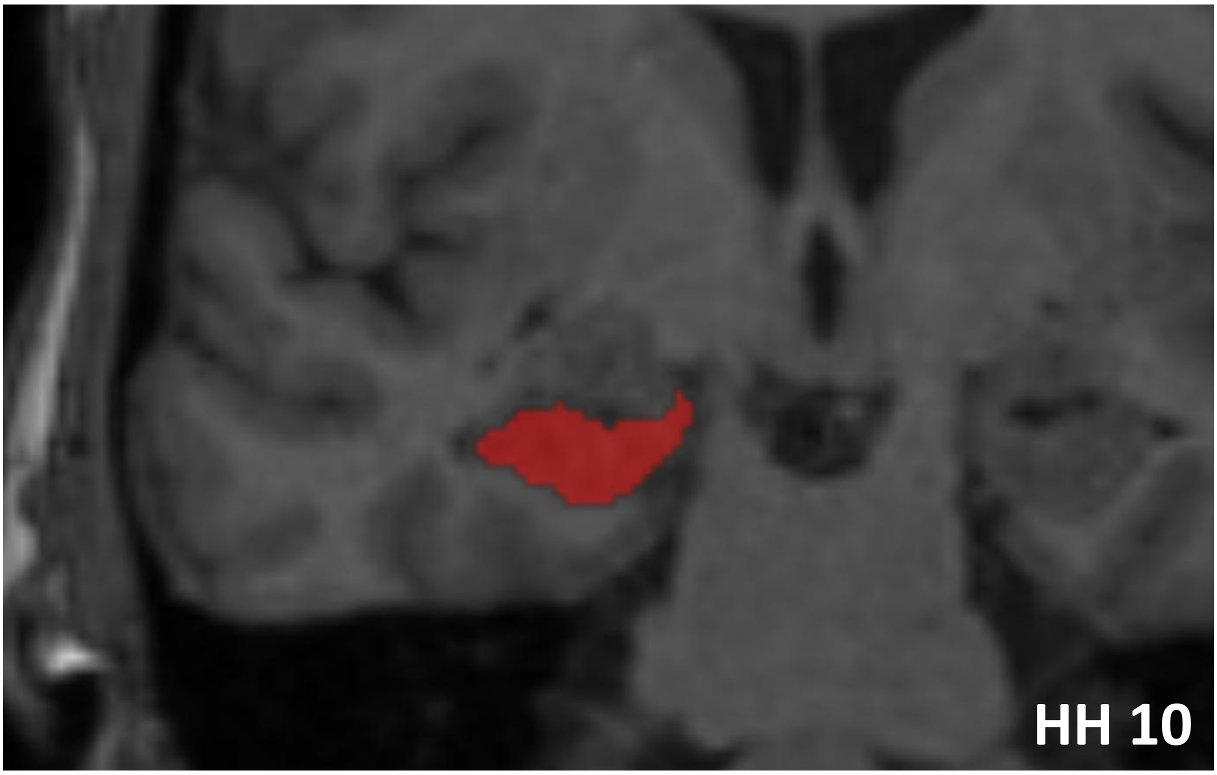
HH 10

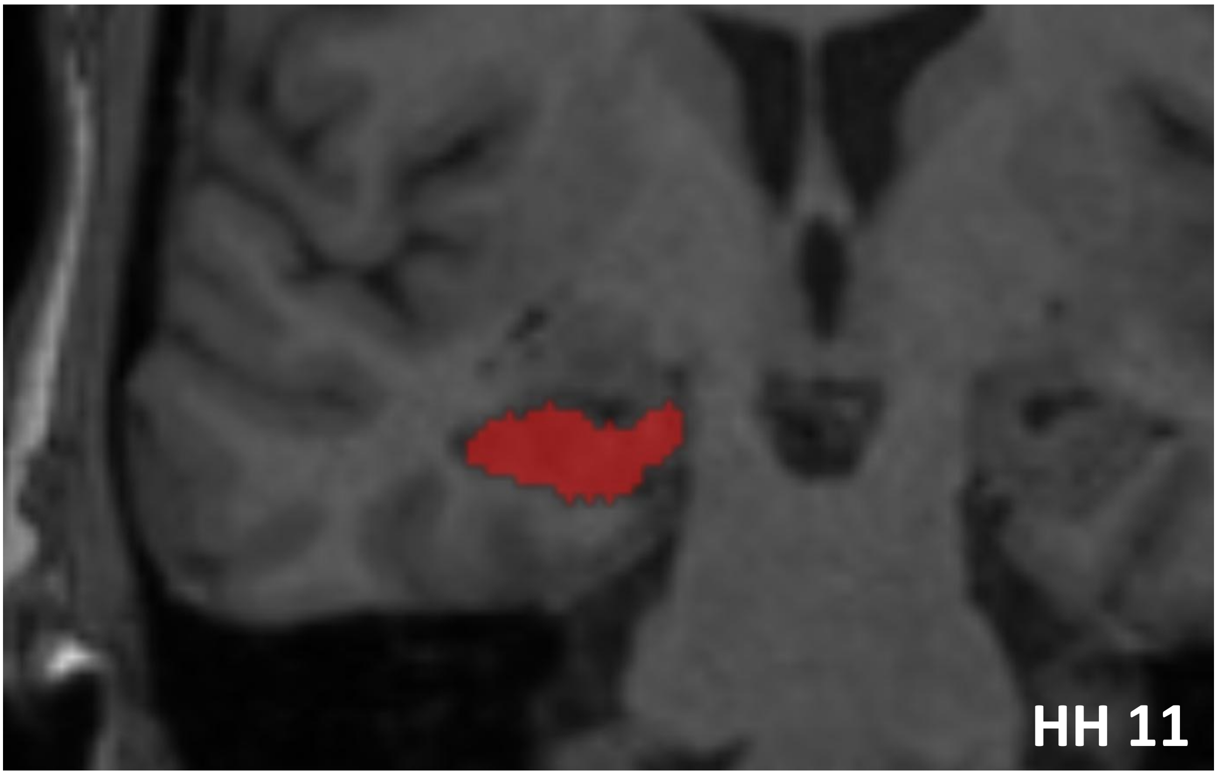
HH 11

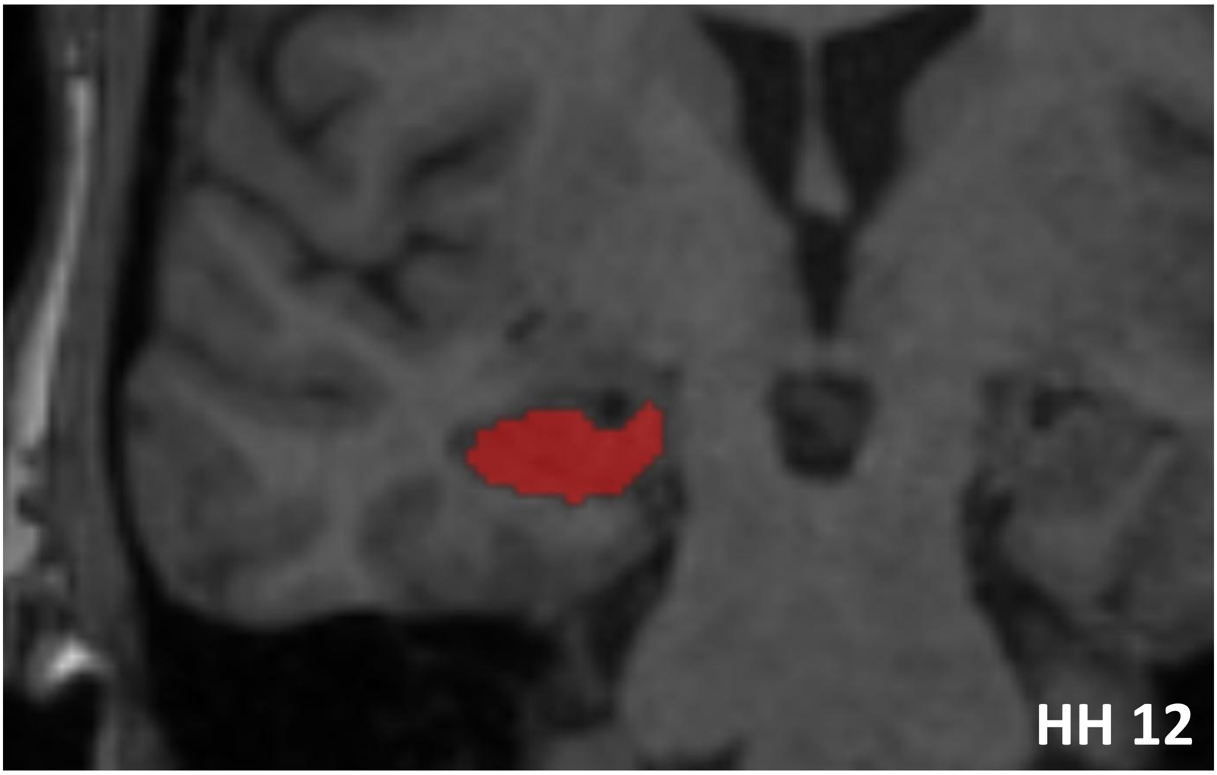
HH 12

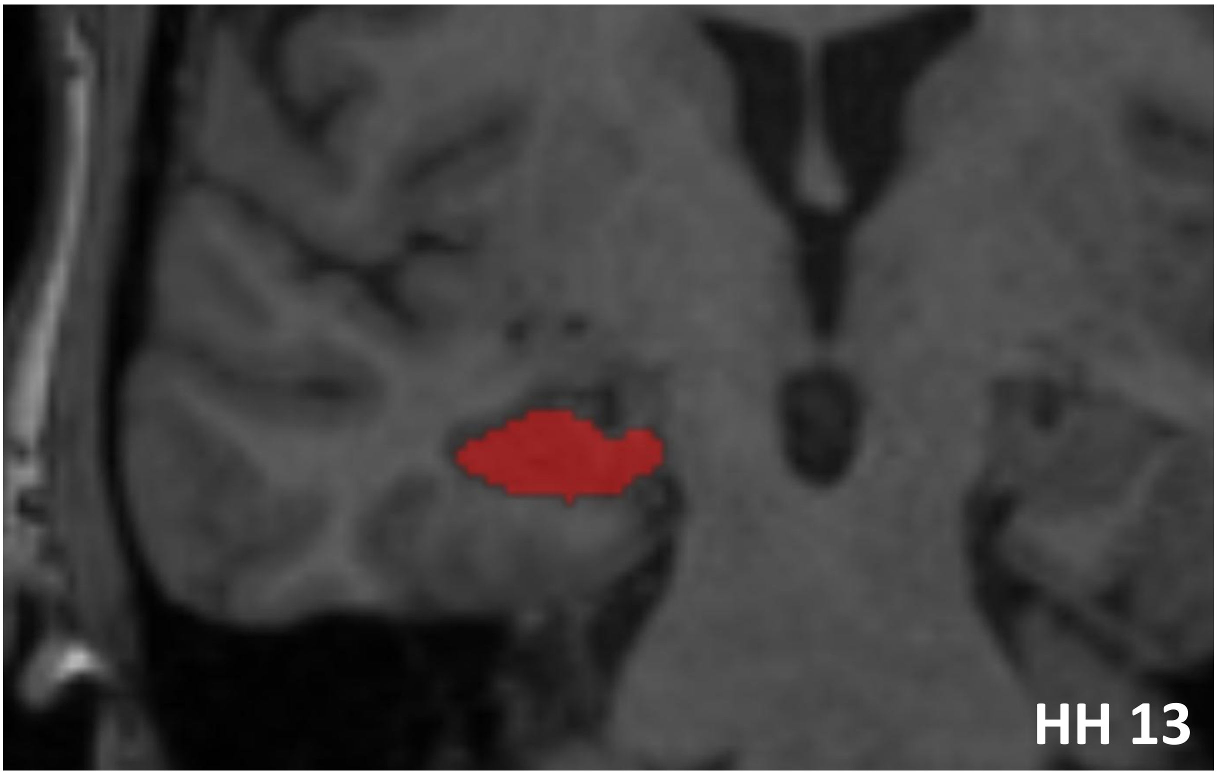
HH 13

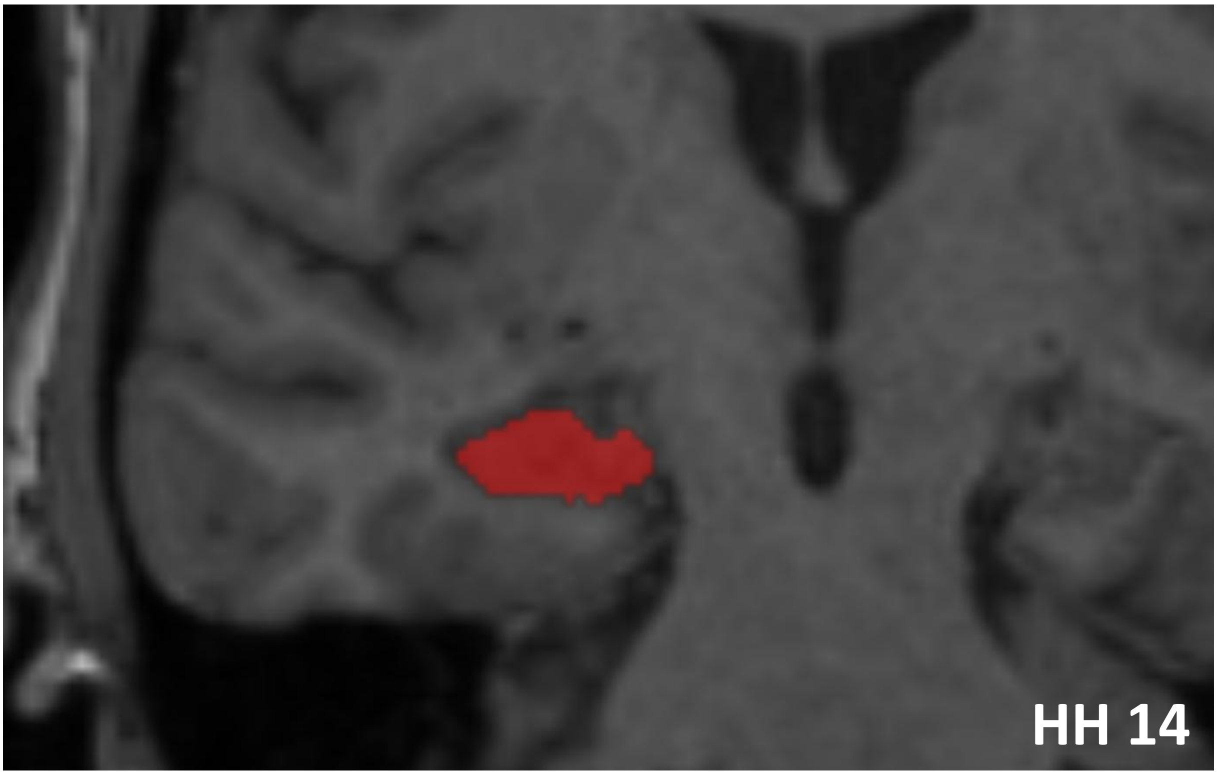
HH 14

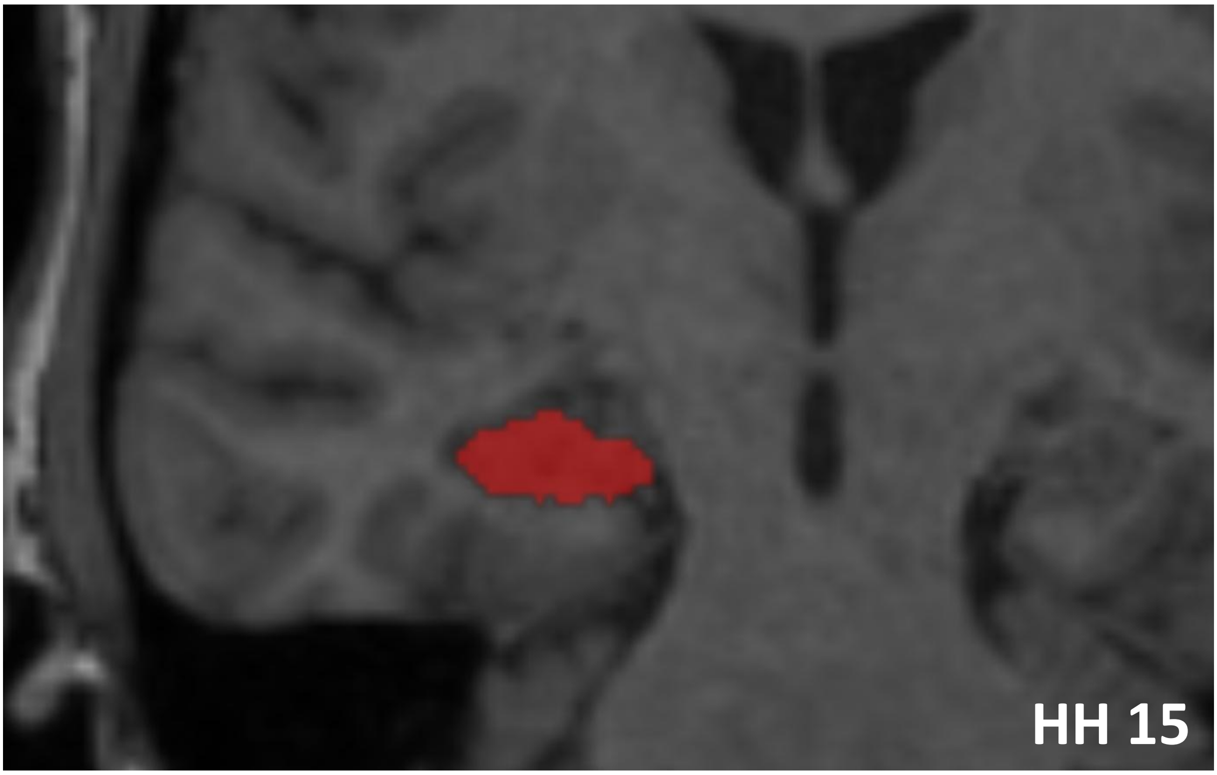
HH 15

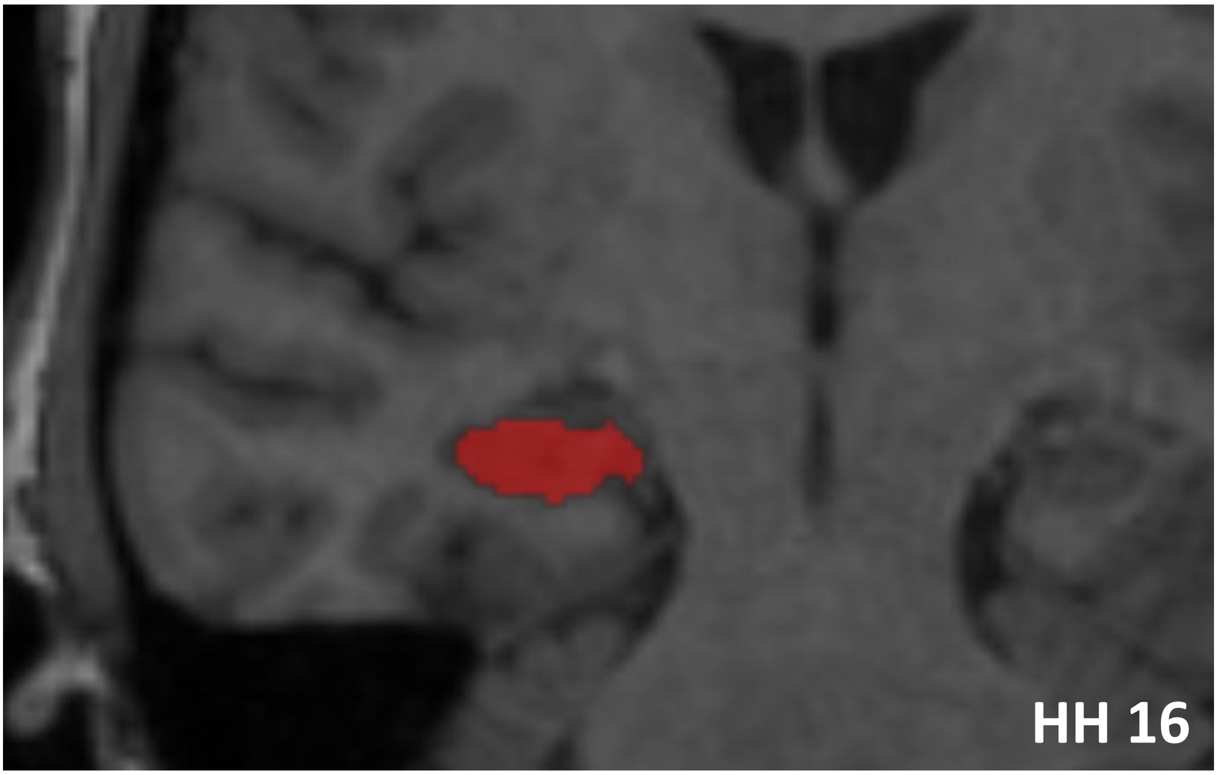
HH 16

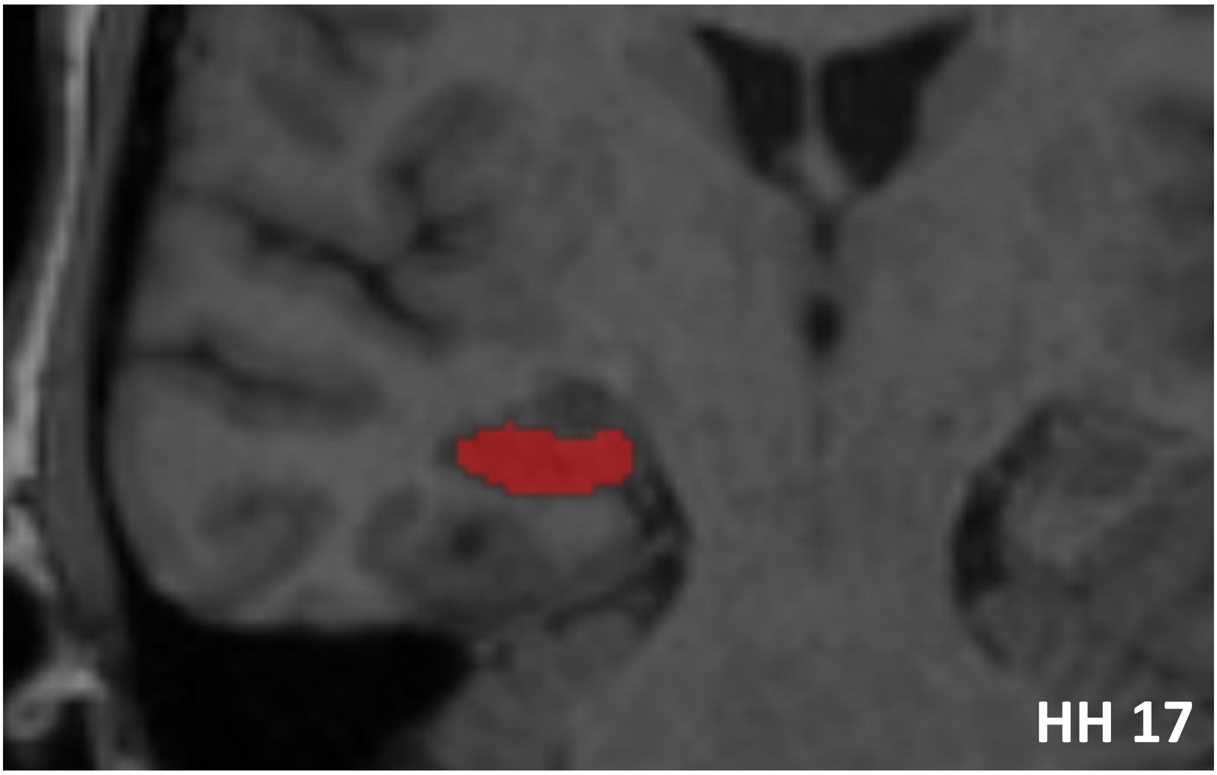
HH 17

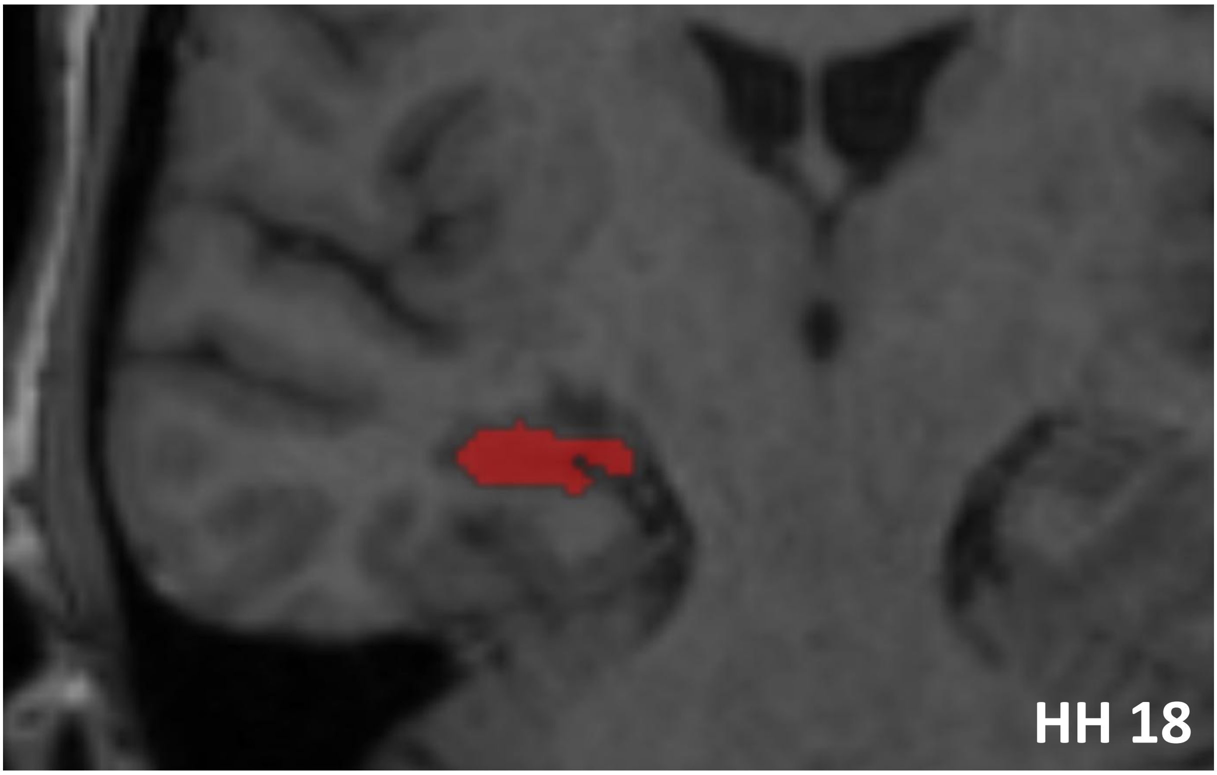
HH 18

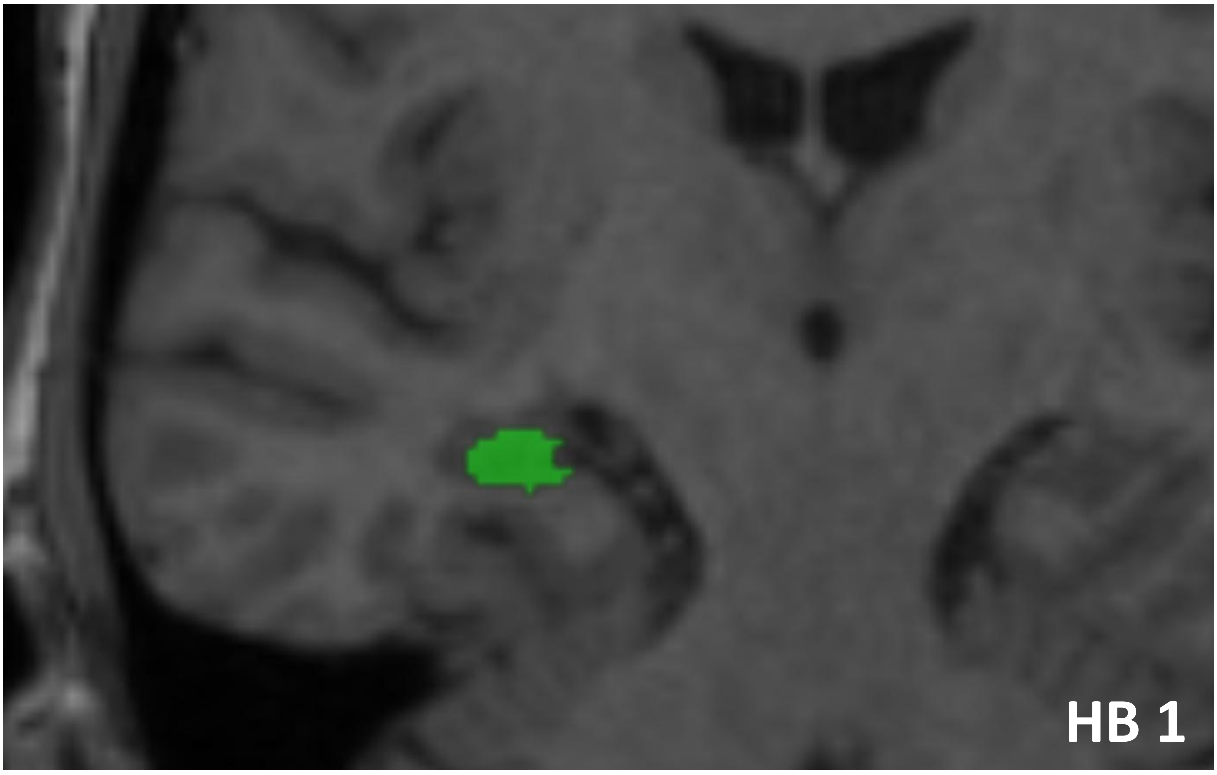
HB 1

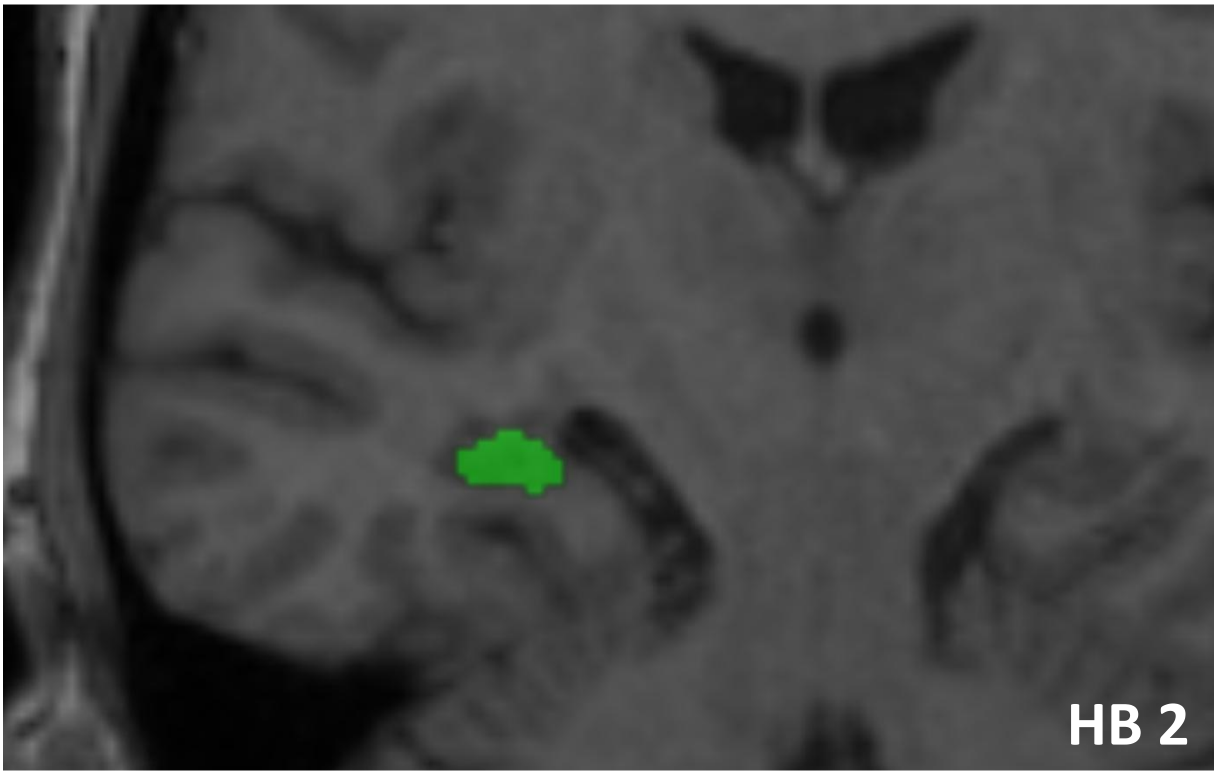
HB 2

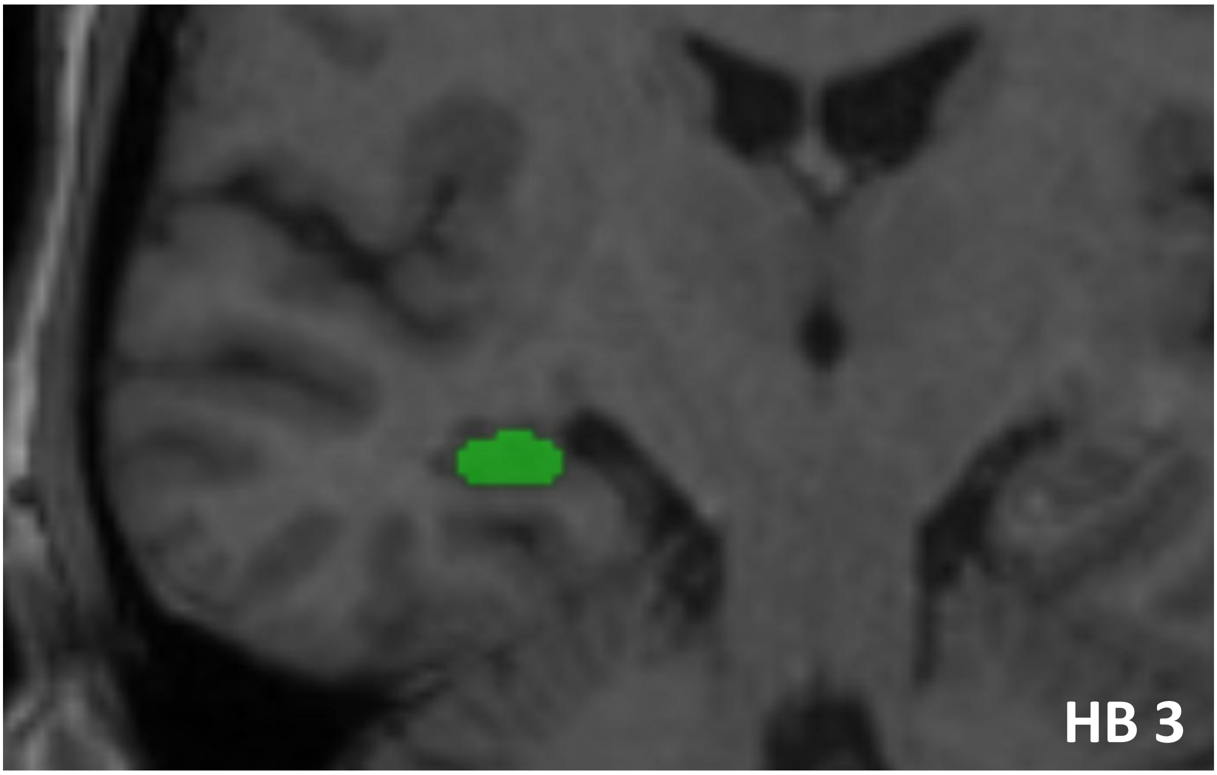
HB 3

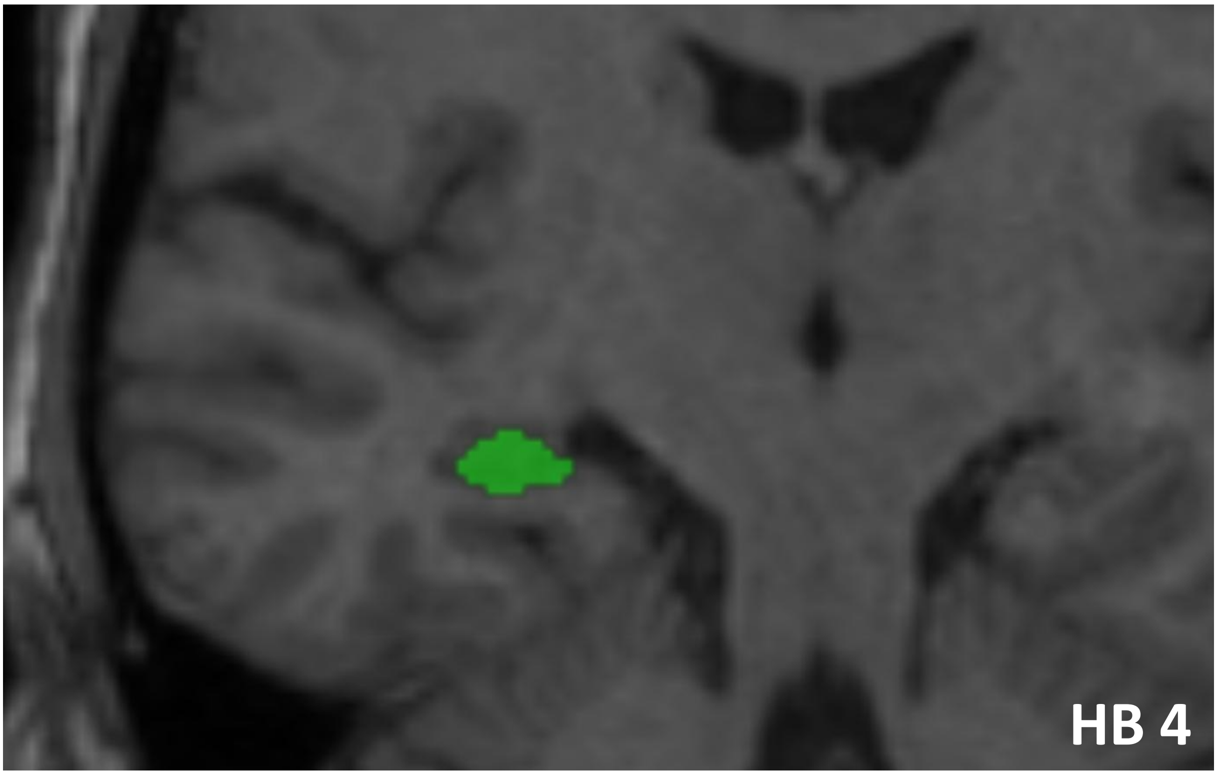
HB 4

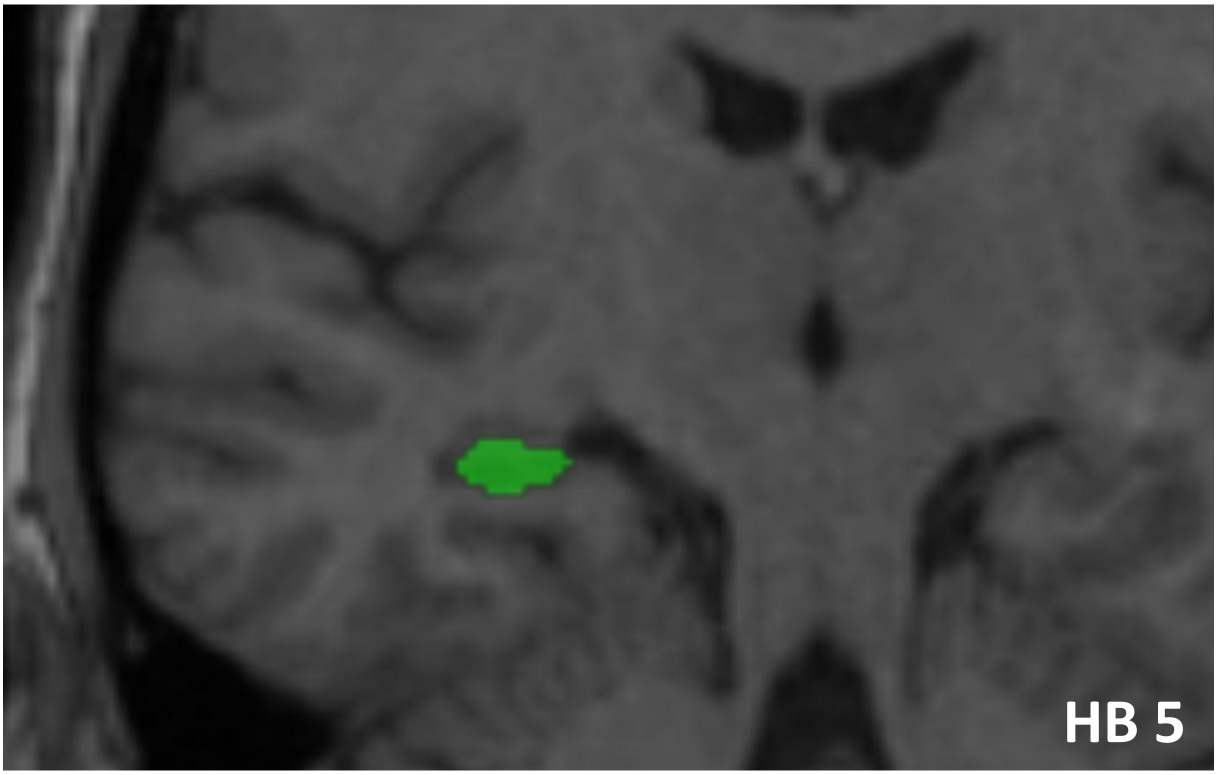
HB 5

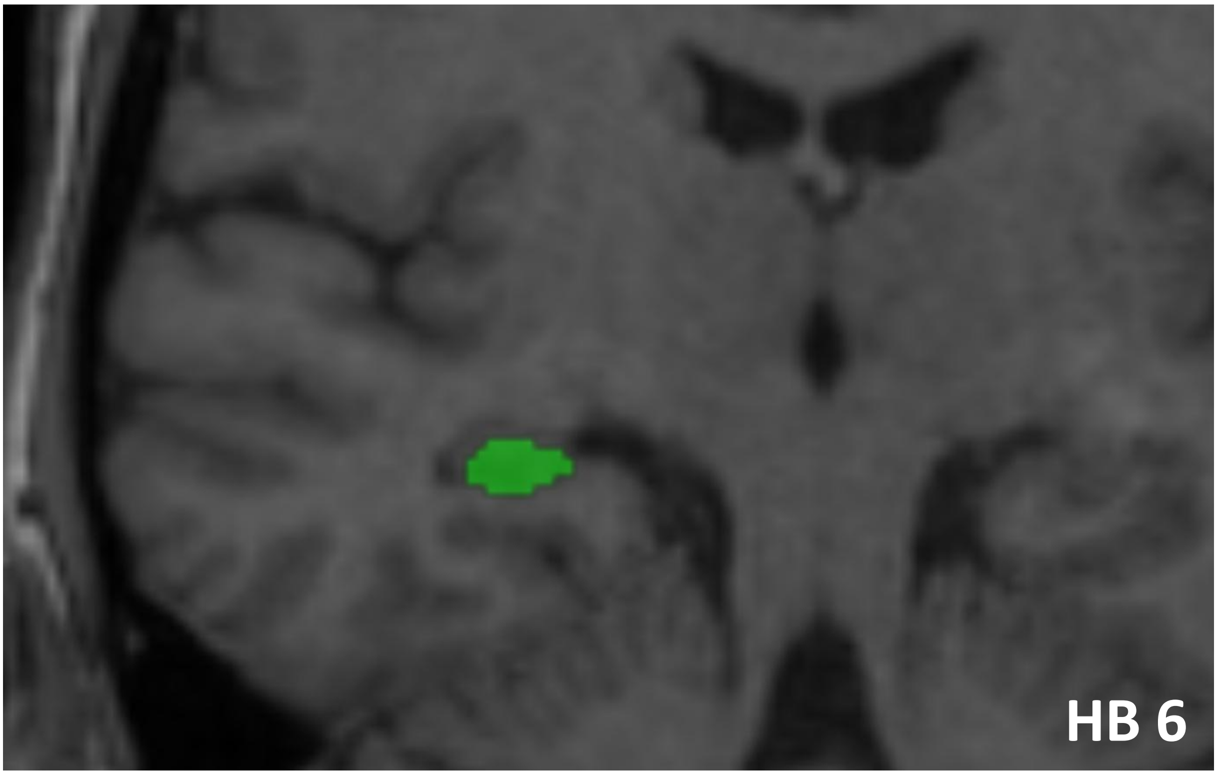
HB 6

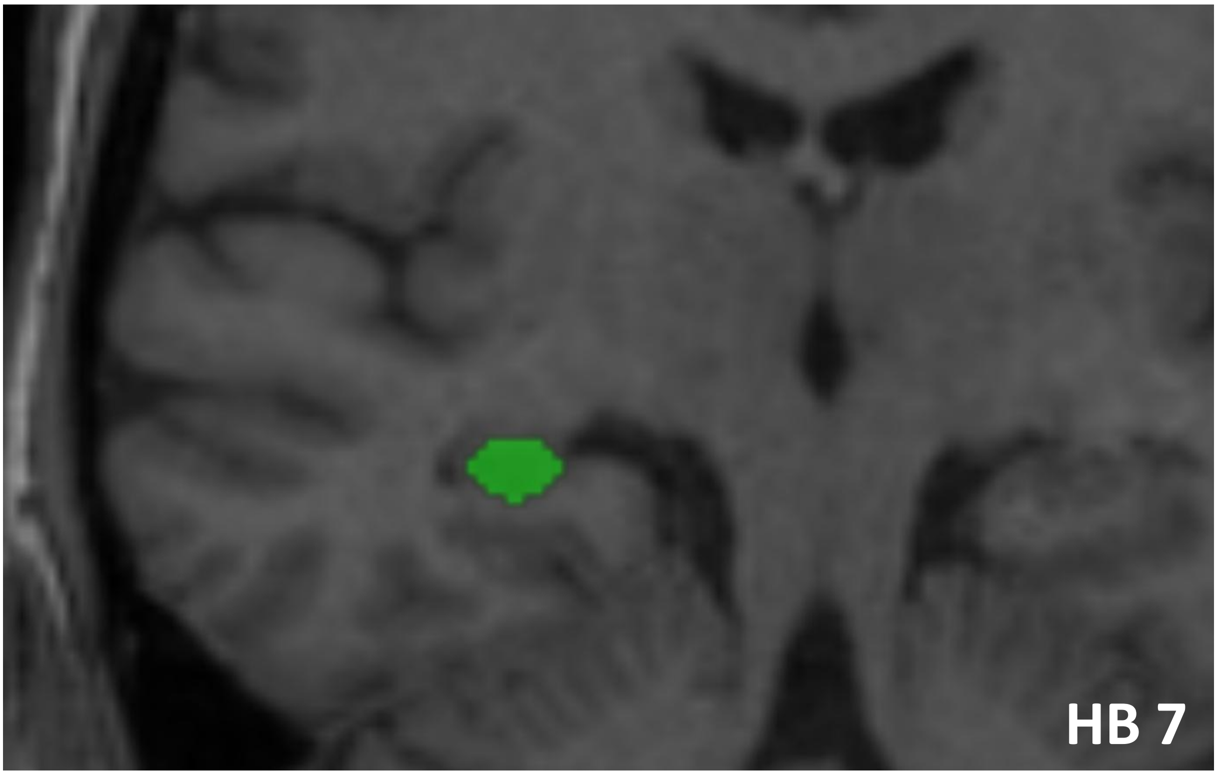
HB 7

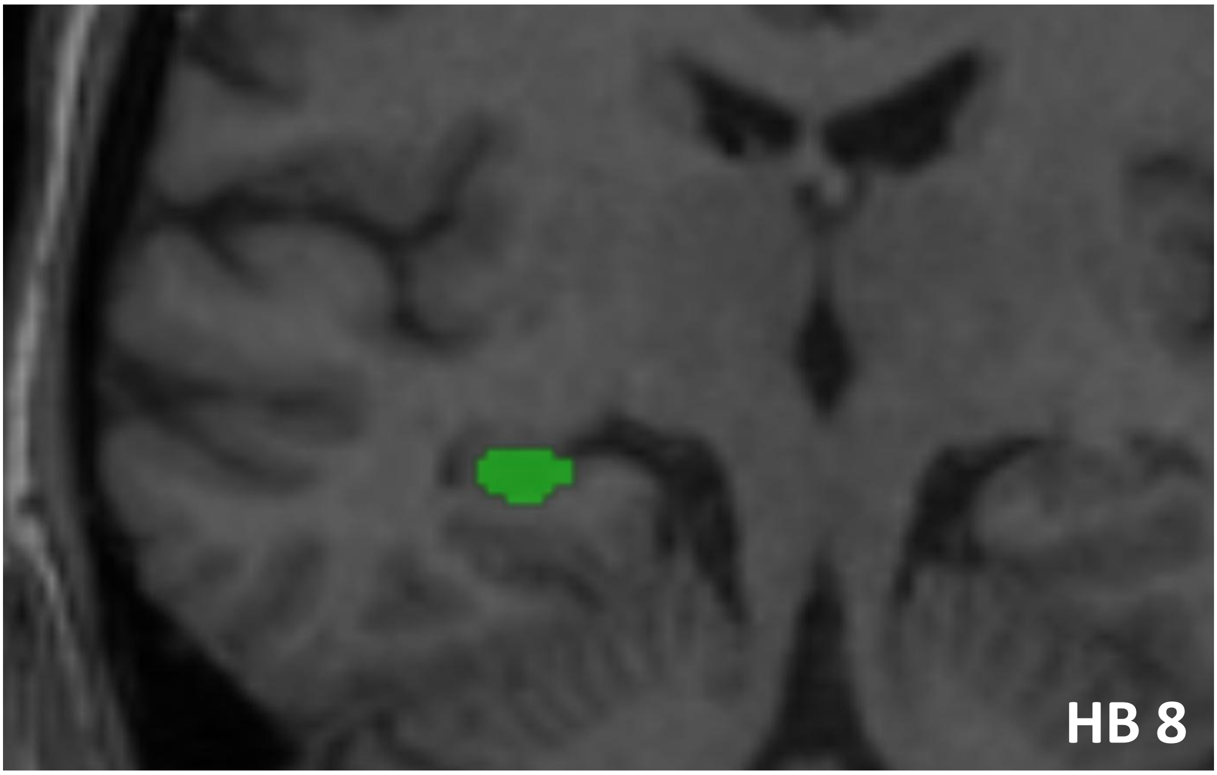
HB 8

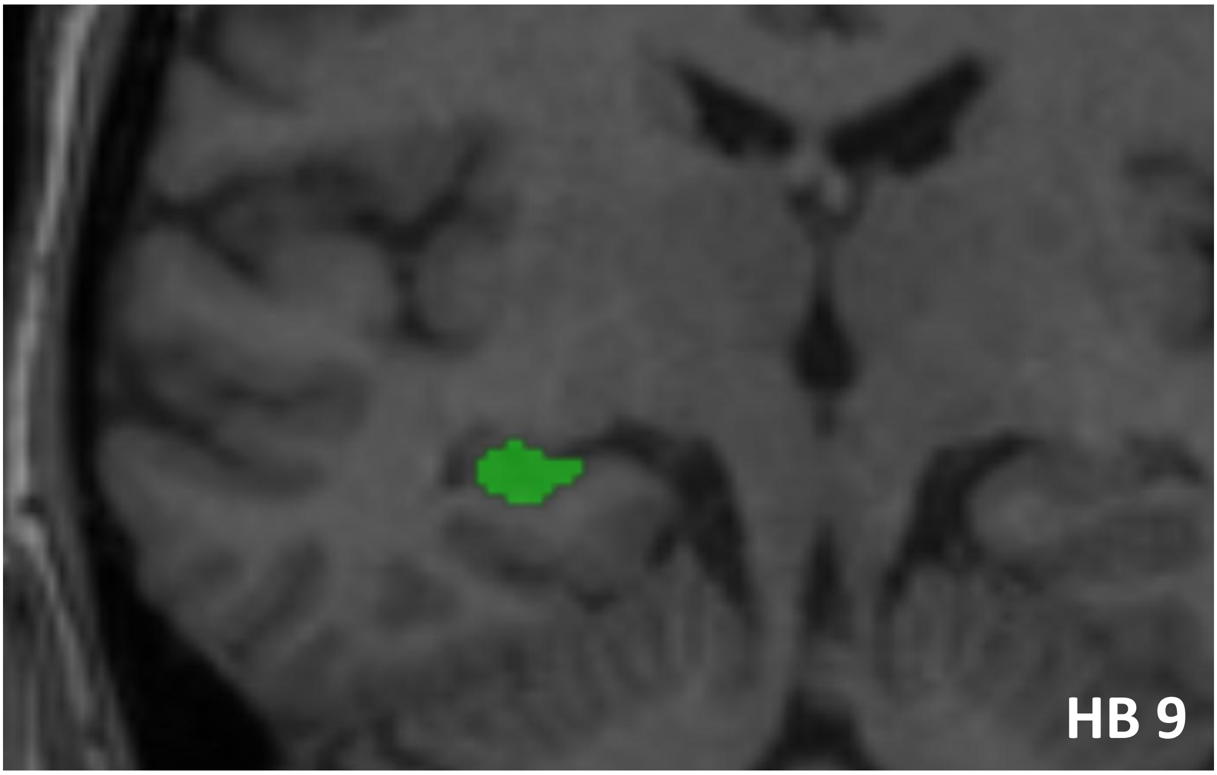
HB 9

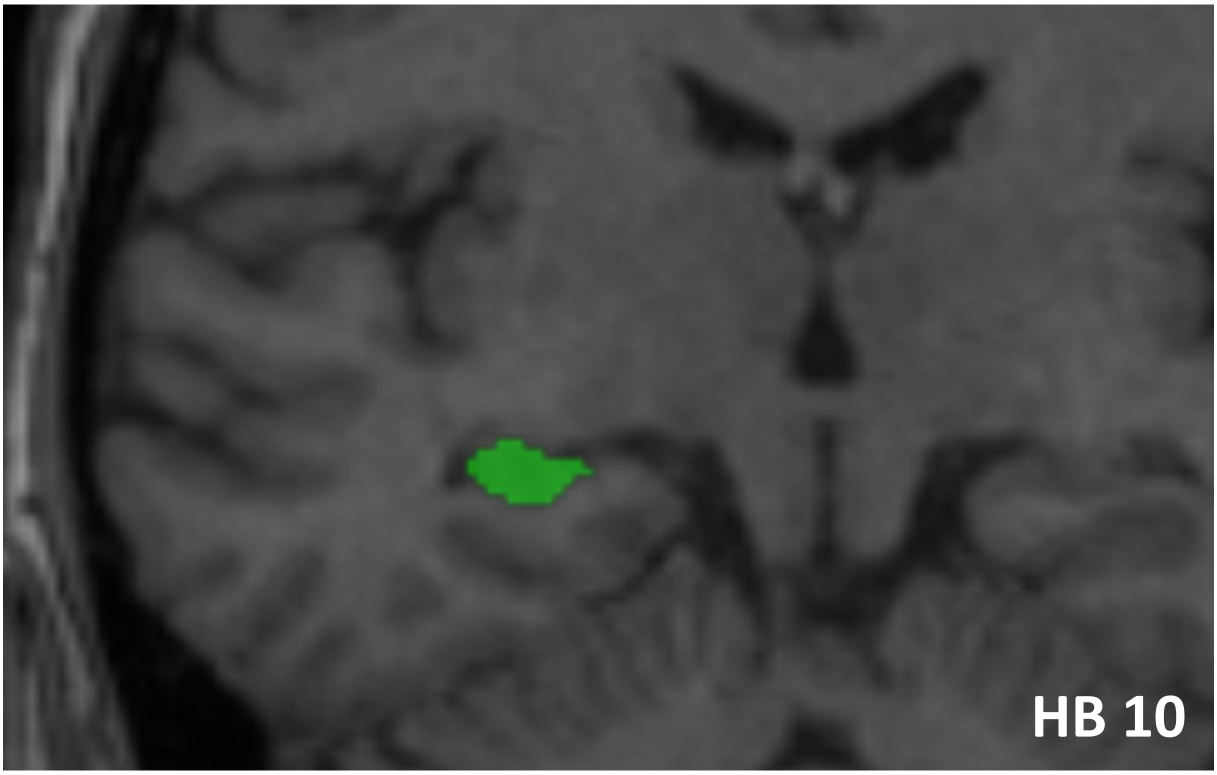
HB 10

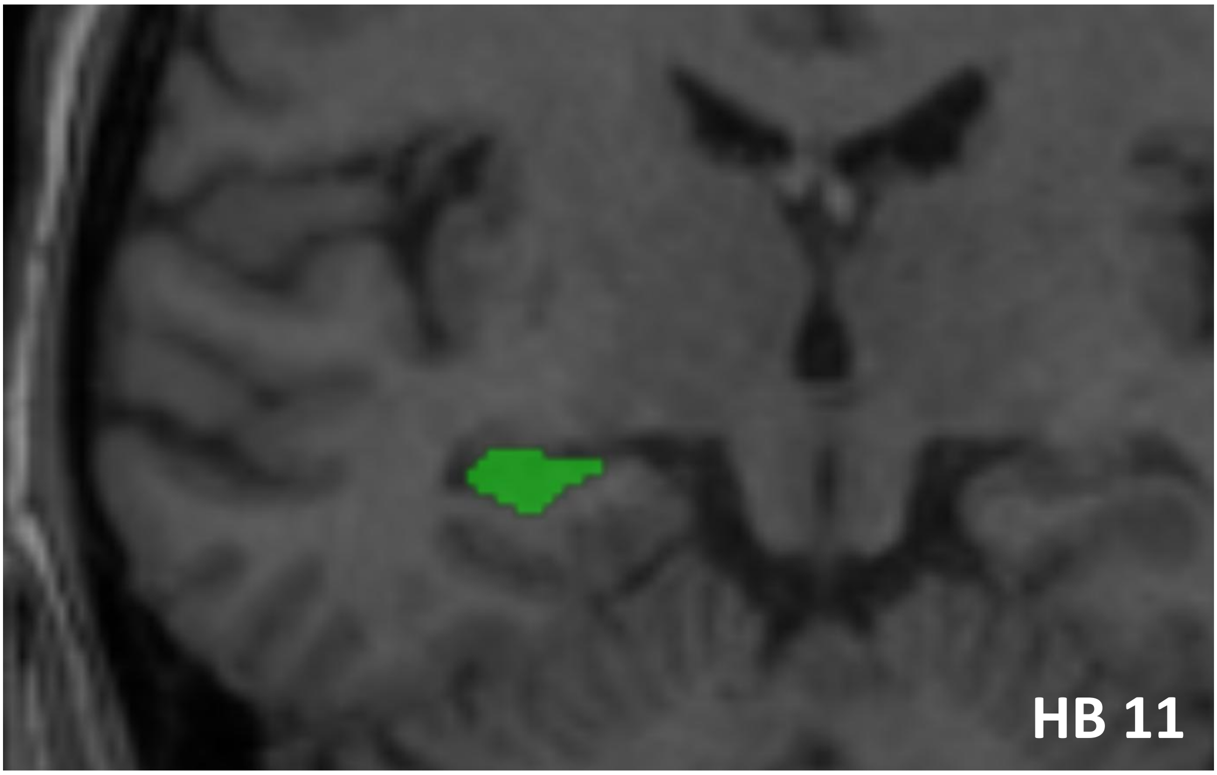
HB 11

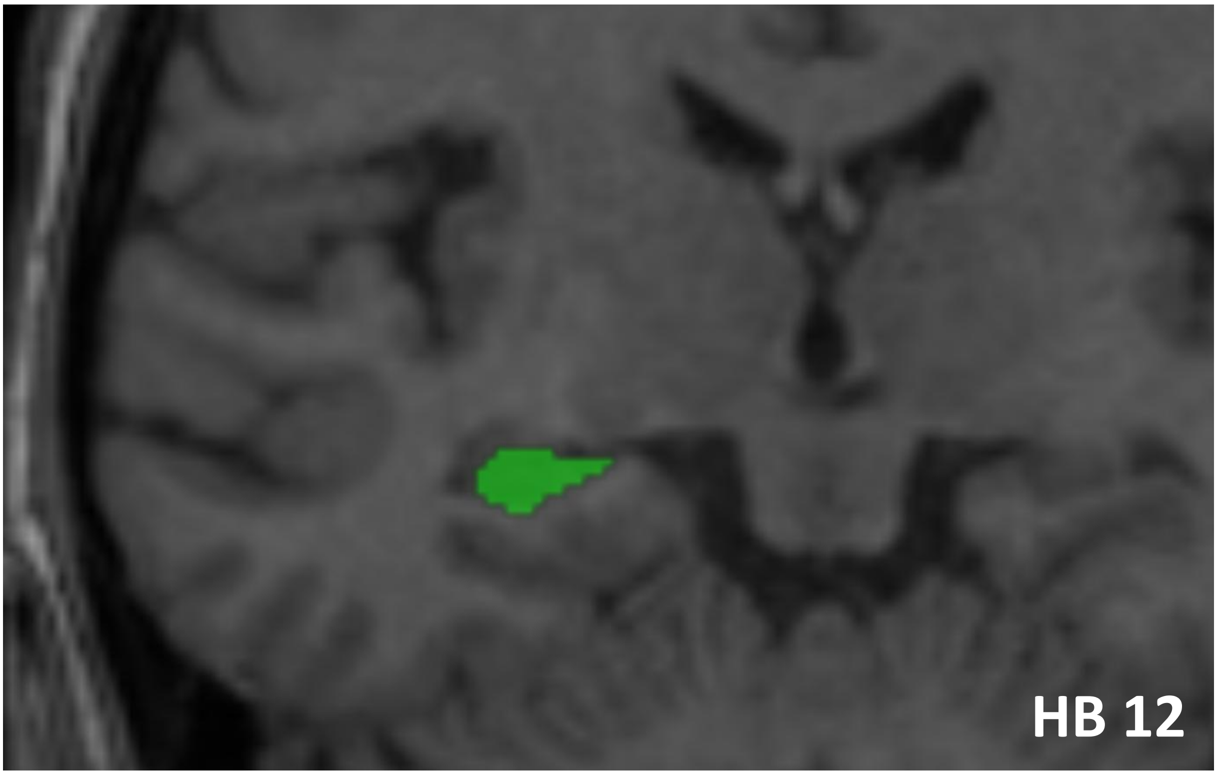
HB 12

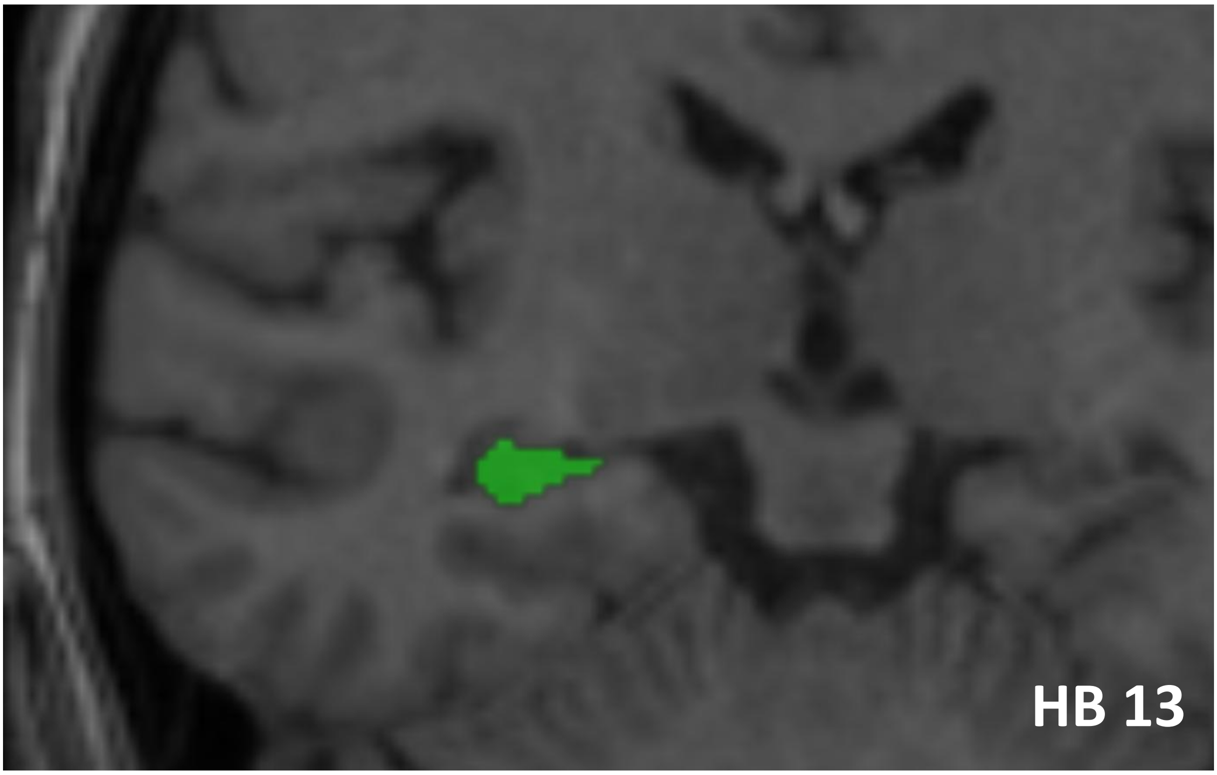
HB 13

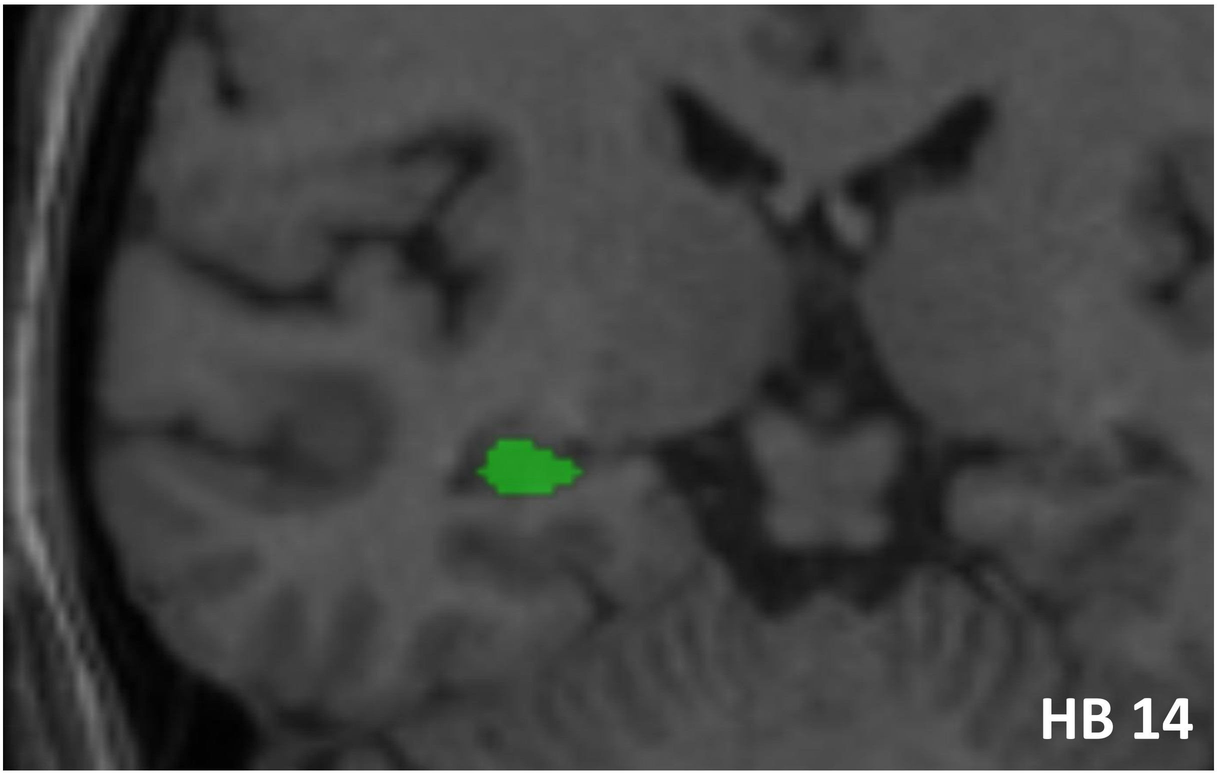
HB 14

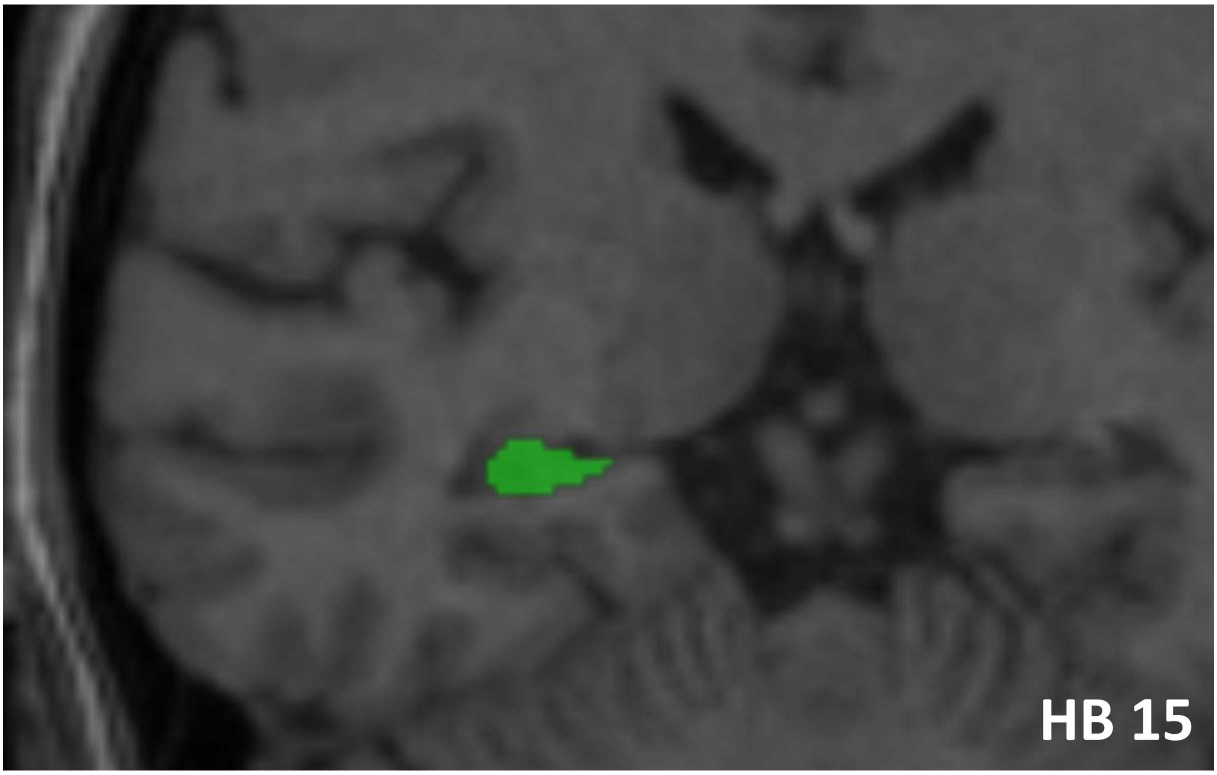
HB 15

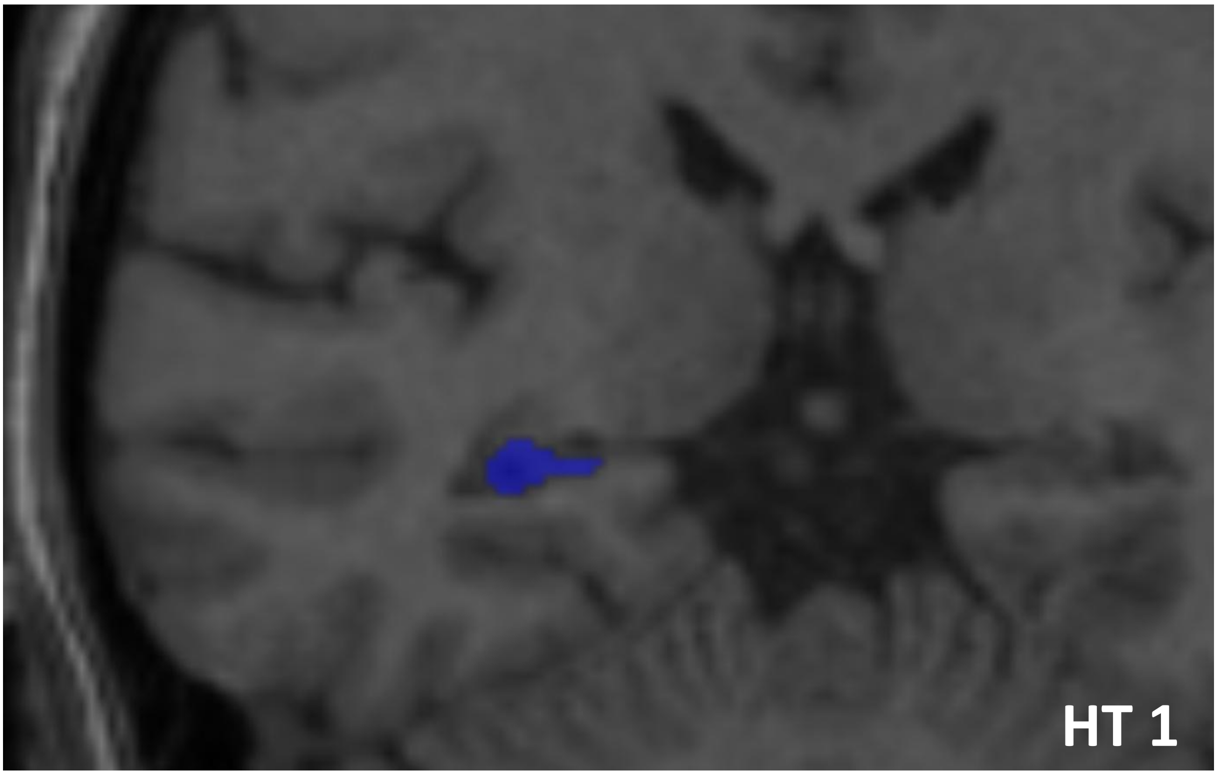
HT 1

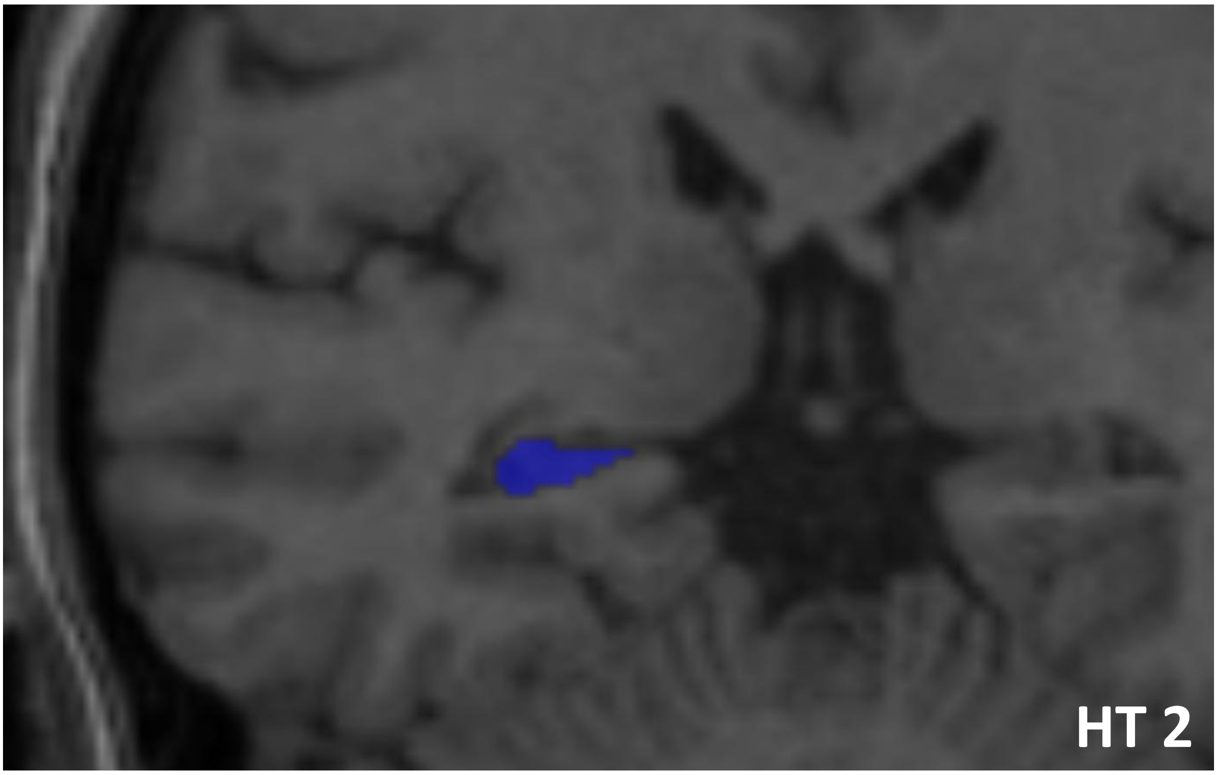
HT 2

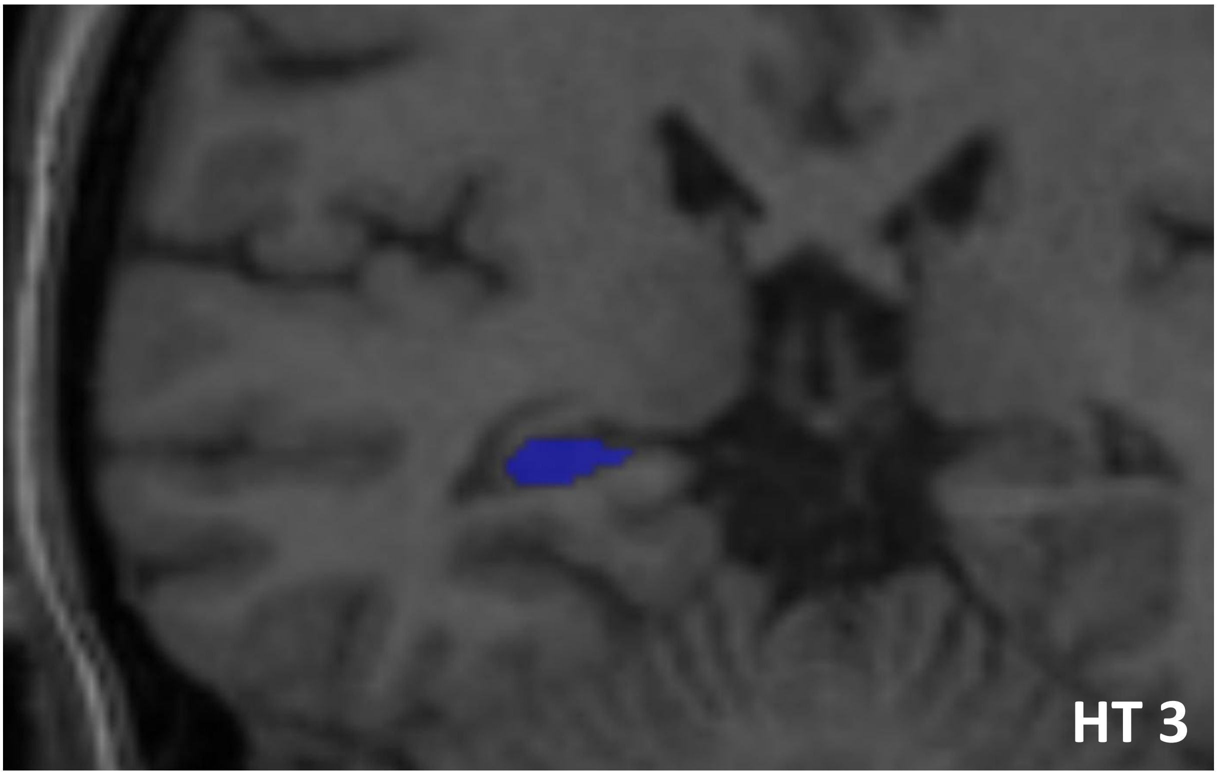
HT 3

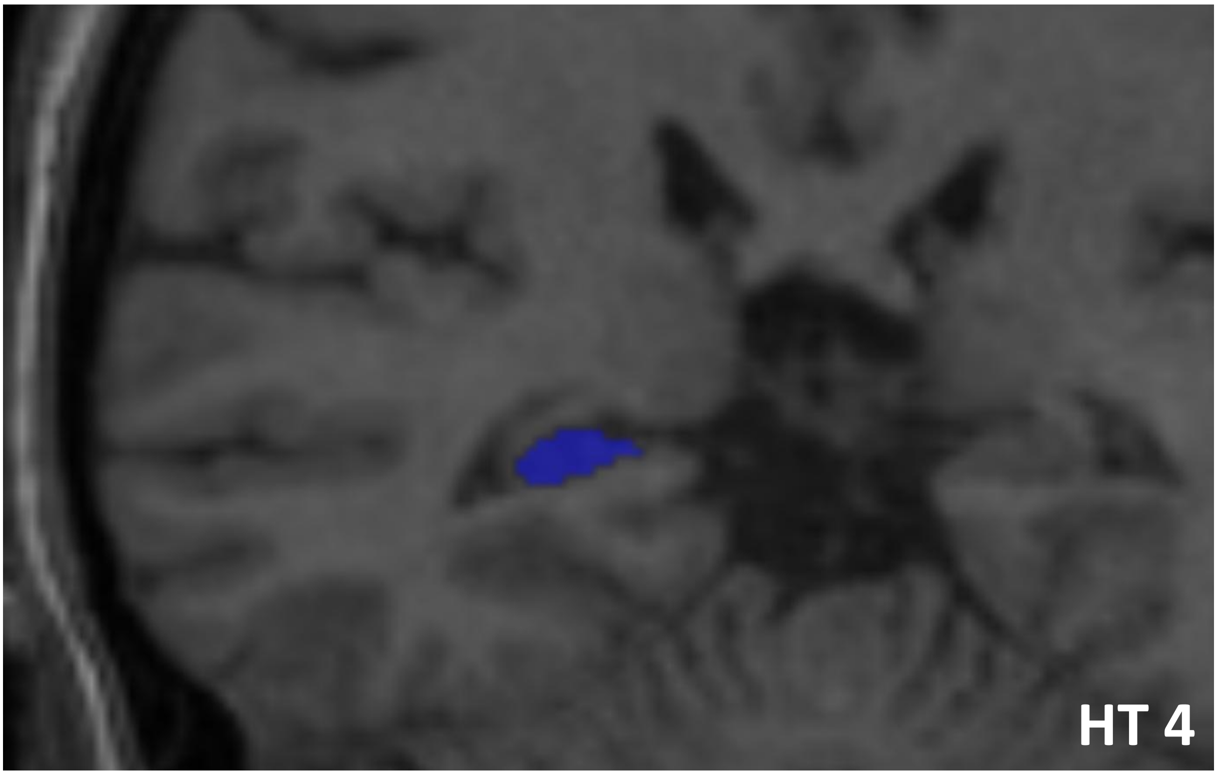
HT 4

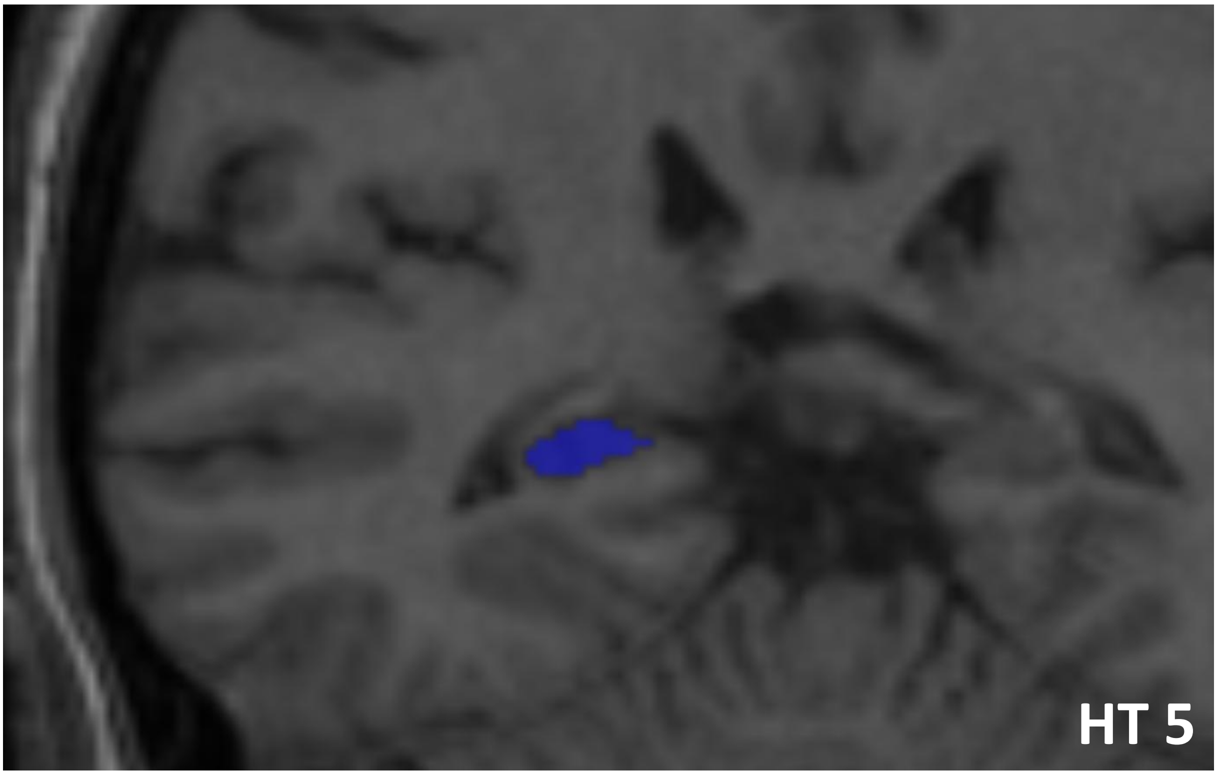
HT 5

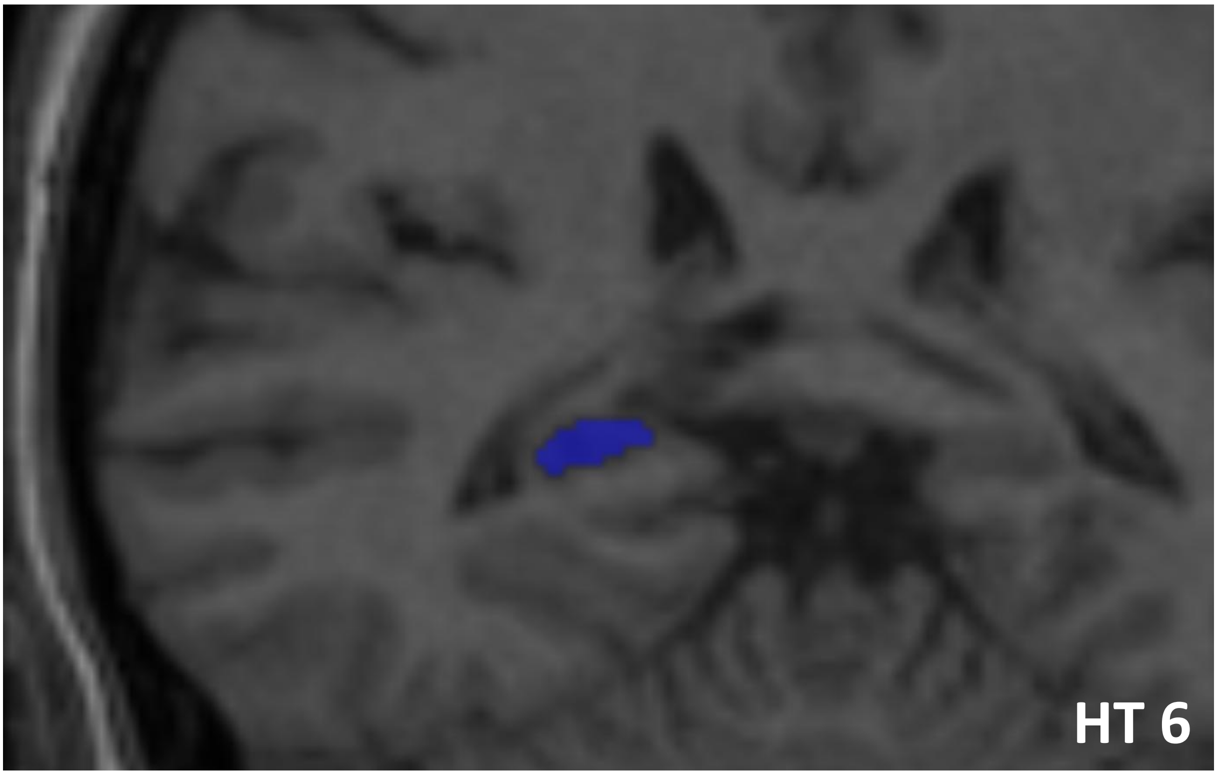
HT 6

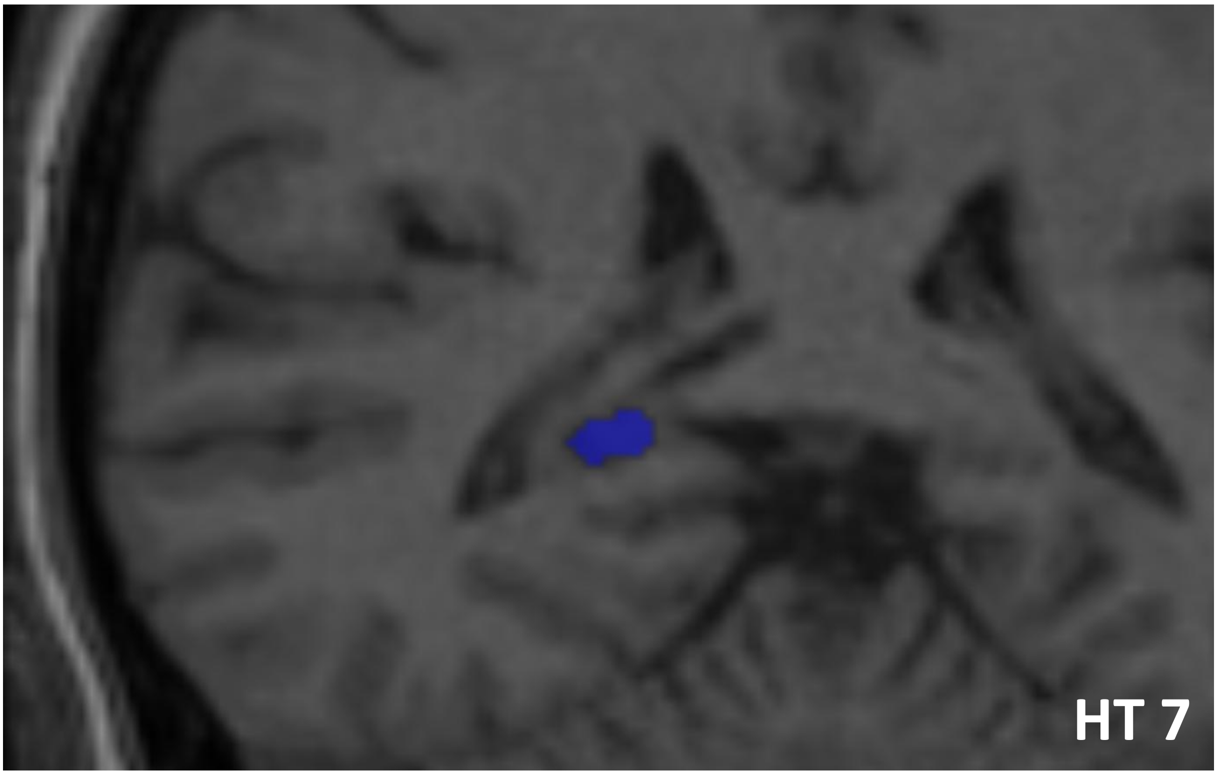
HT 7

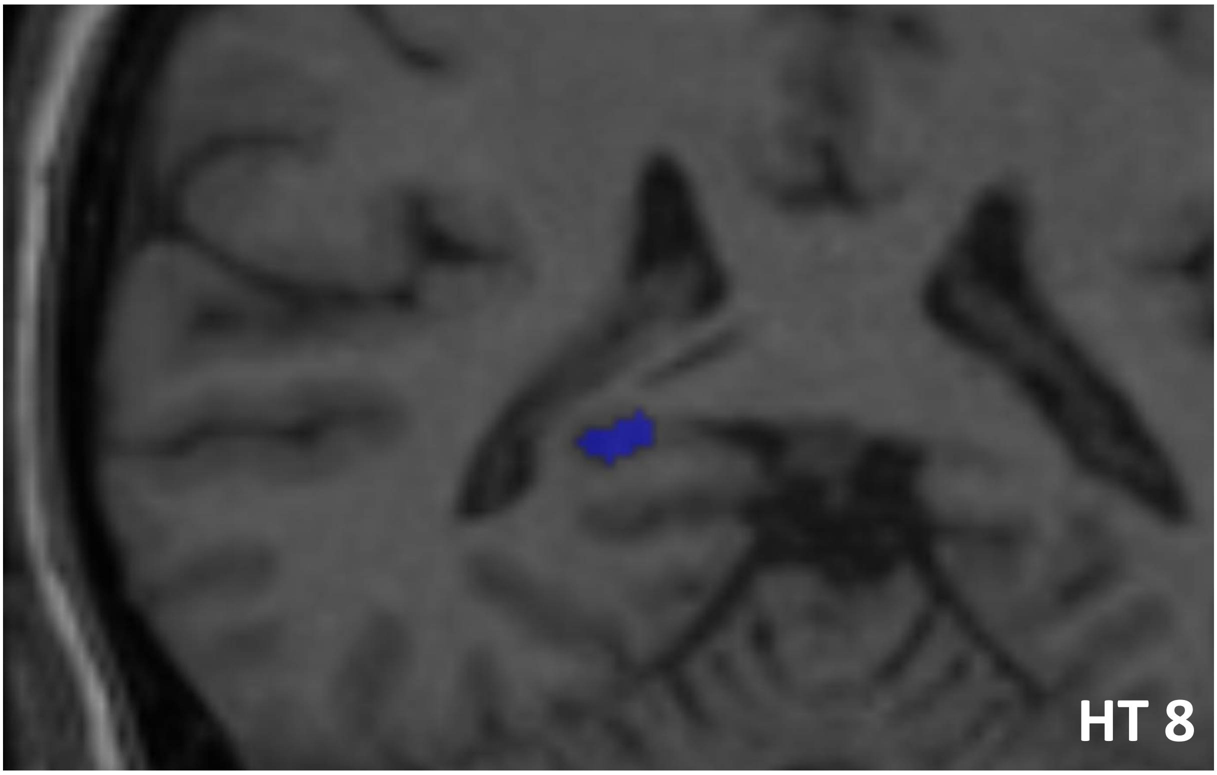
HT 8
