# Supplementary material for: Spatial Pattern Separation Testing Differentiates Alzheimer’s Disease Biomarker-Positive and Biomarker-Negative Older Adults With Amnestic Mild Cognitive Impairment
Source: Front Aging Neurosci. 2021 Nov 26;13:774600. doi: 10.3389/fnagi.2021.774600 (PMC8662816; doi:10.3389/fnagi.2021.774600)
Supplement: Supplementary file 2 [file Image_2.pdf]

Supplementary Figure 2: Slice-by-slice segmentation of the entorhinal cortex

Anterolateral entorhinal cortex

Posteromedial entorhinal cortex

Hippocampal head

Hippocampal body

EC - Entorhinal cortex

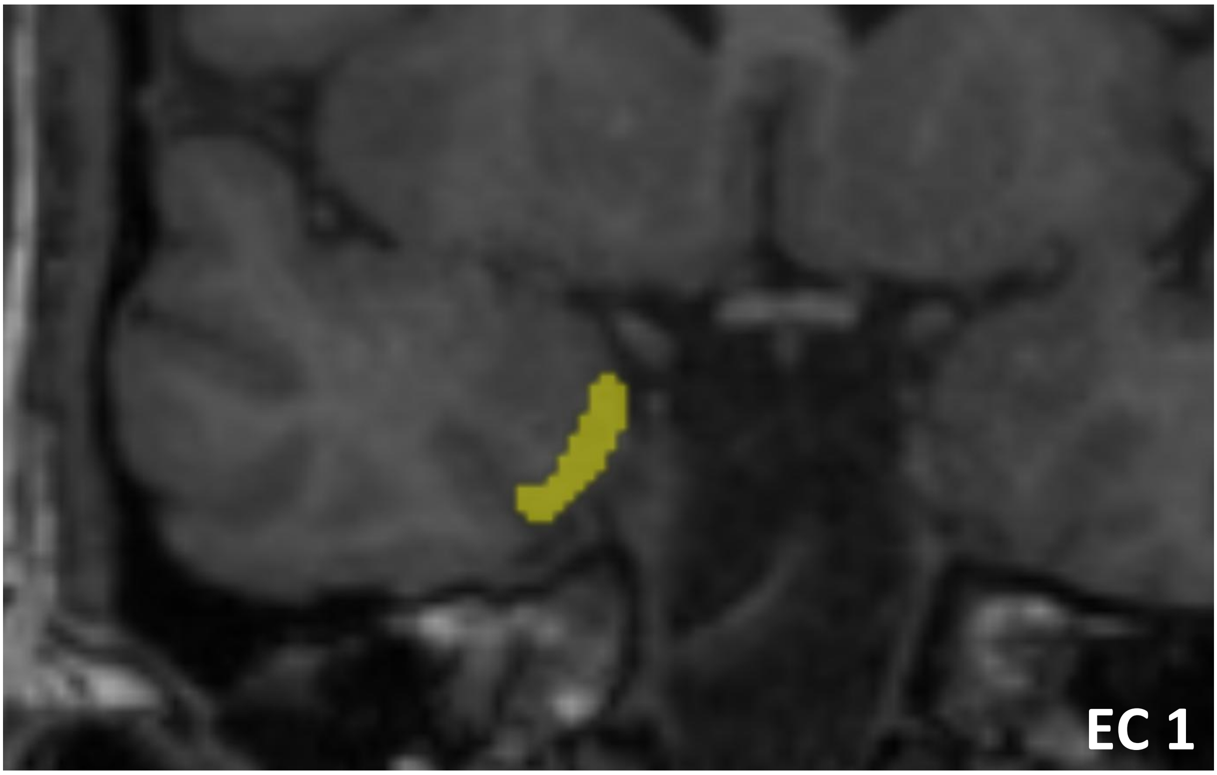

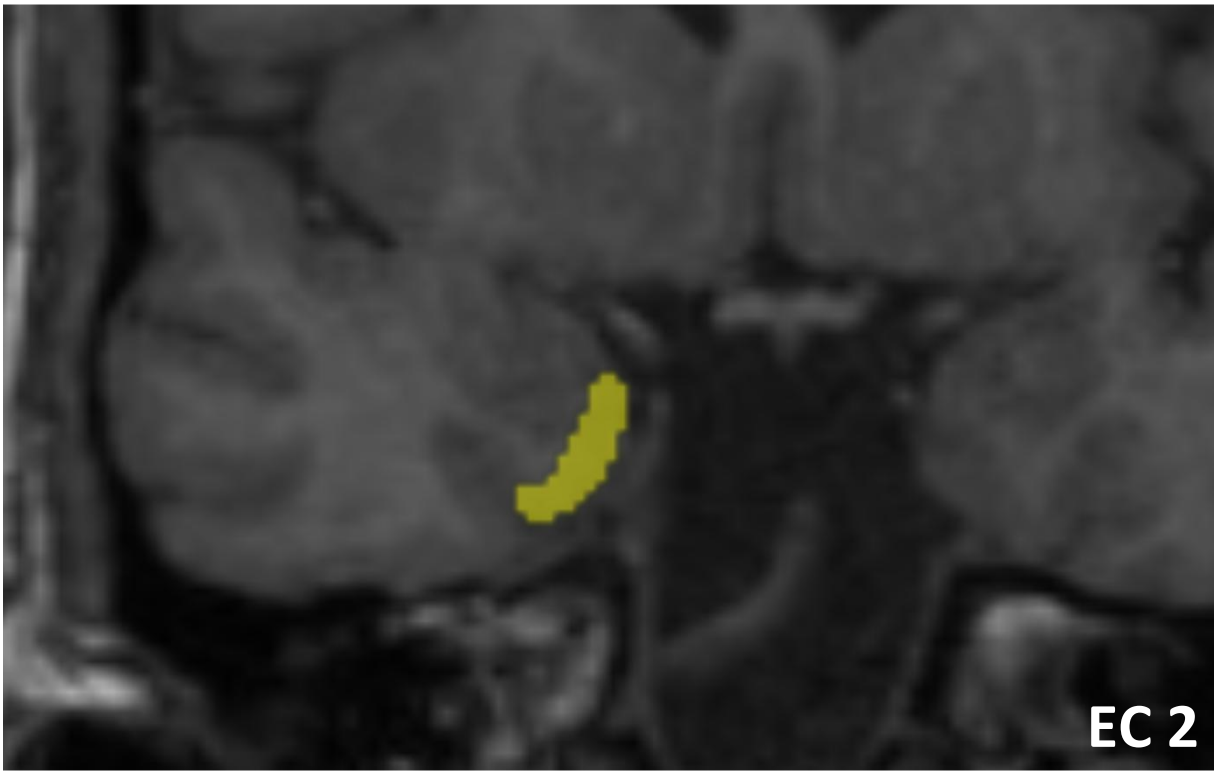
EC 2

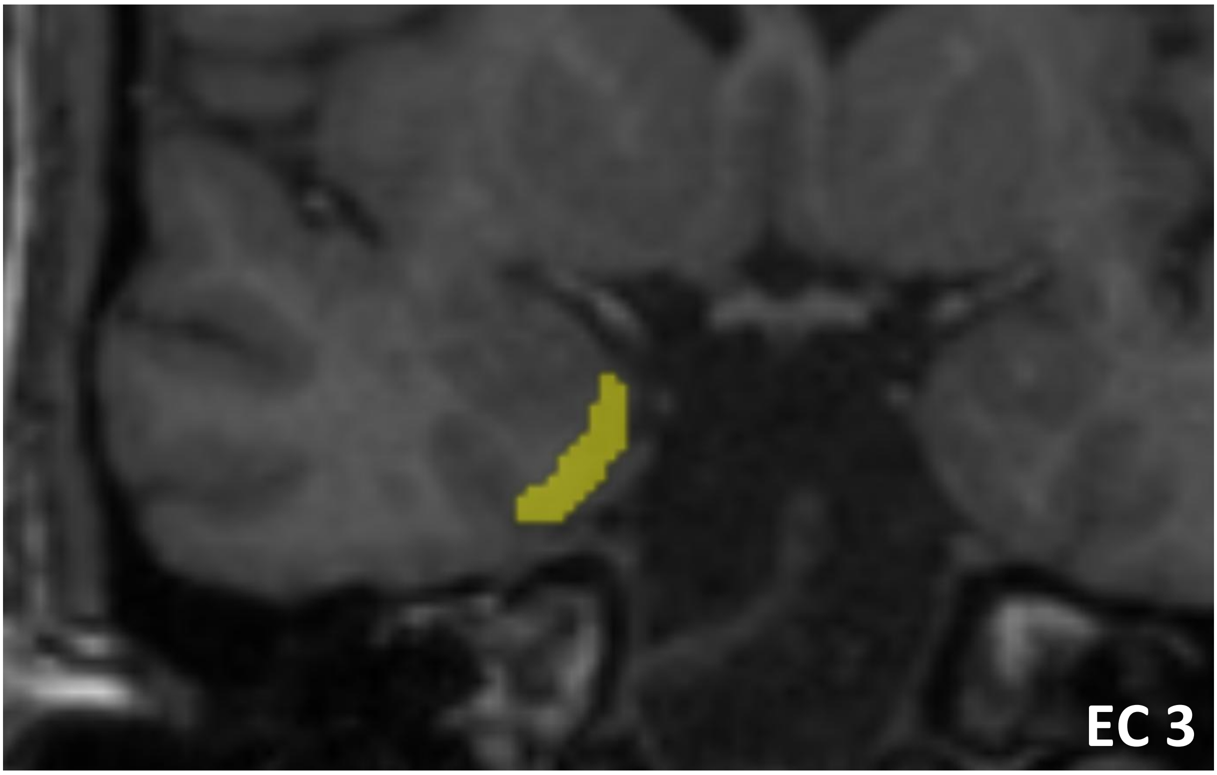
EC 3

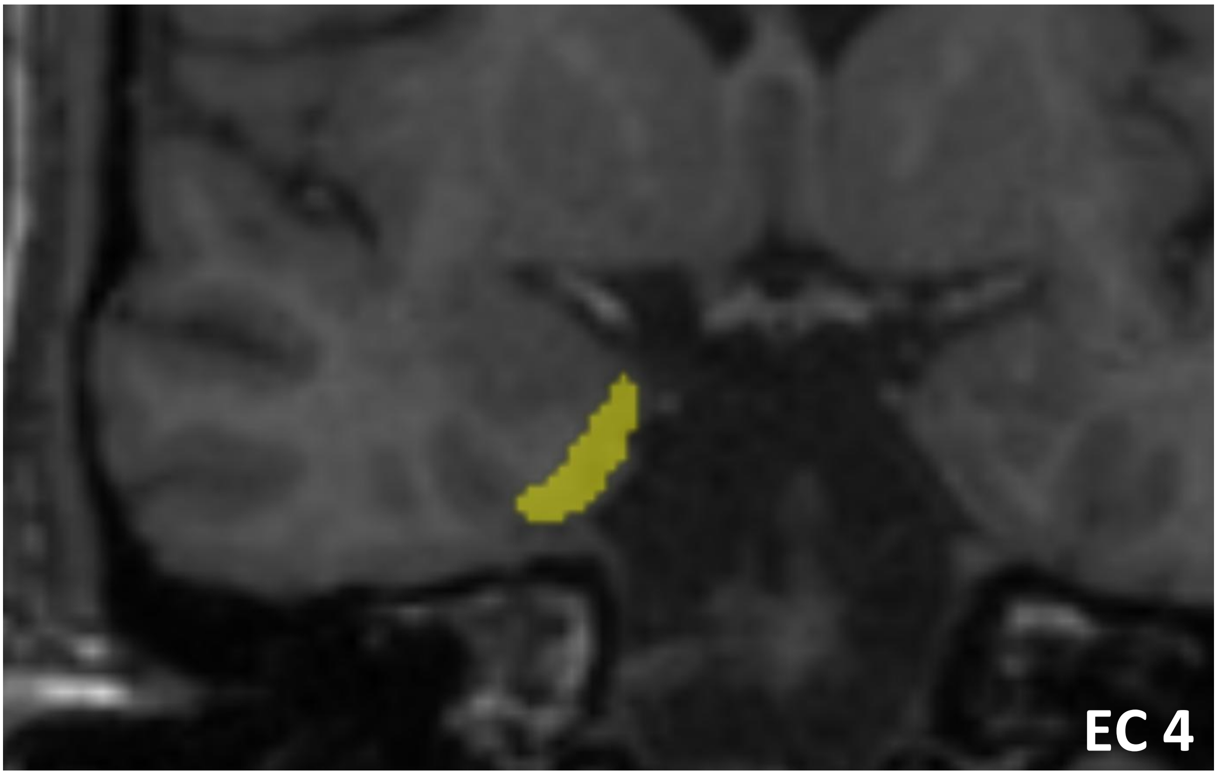
EC 4

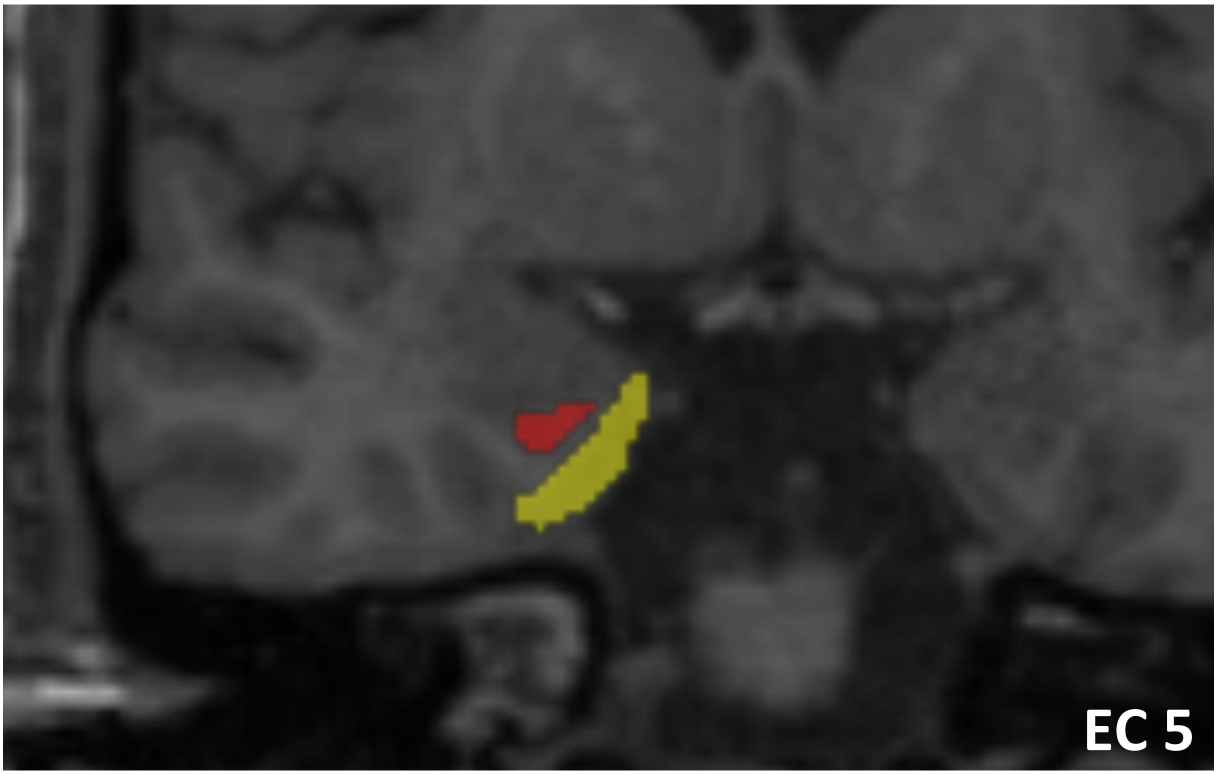
EC 5

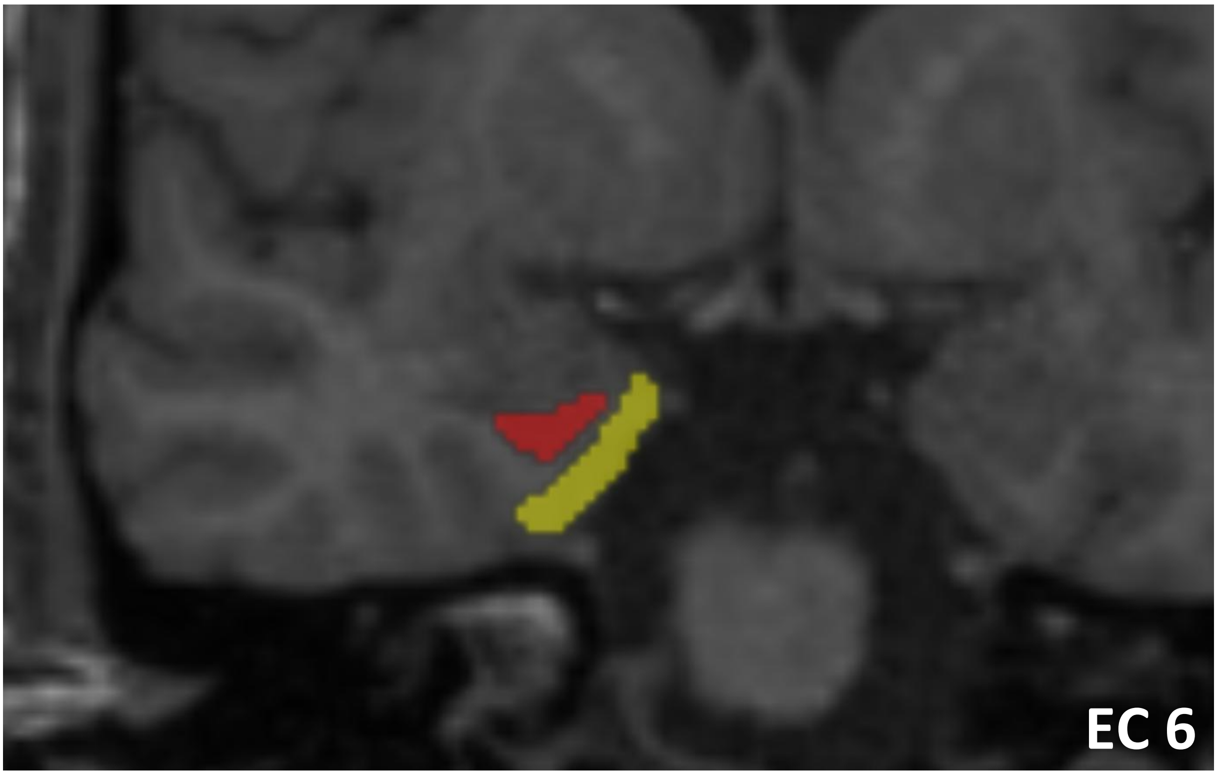
EC 6

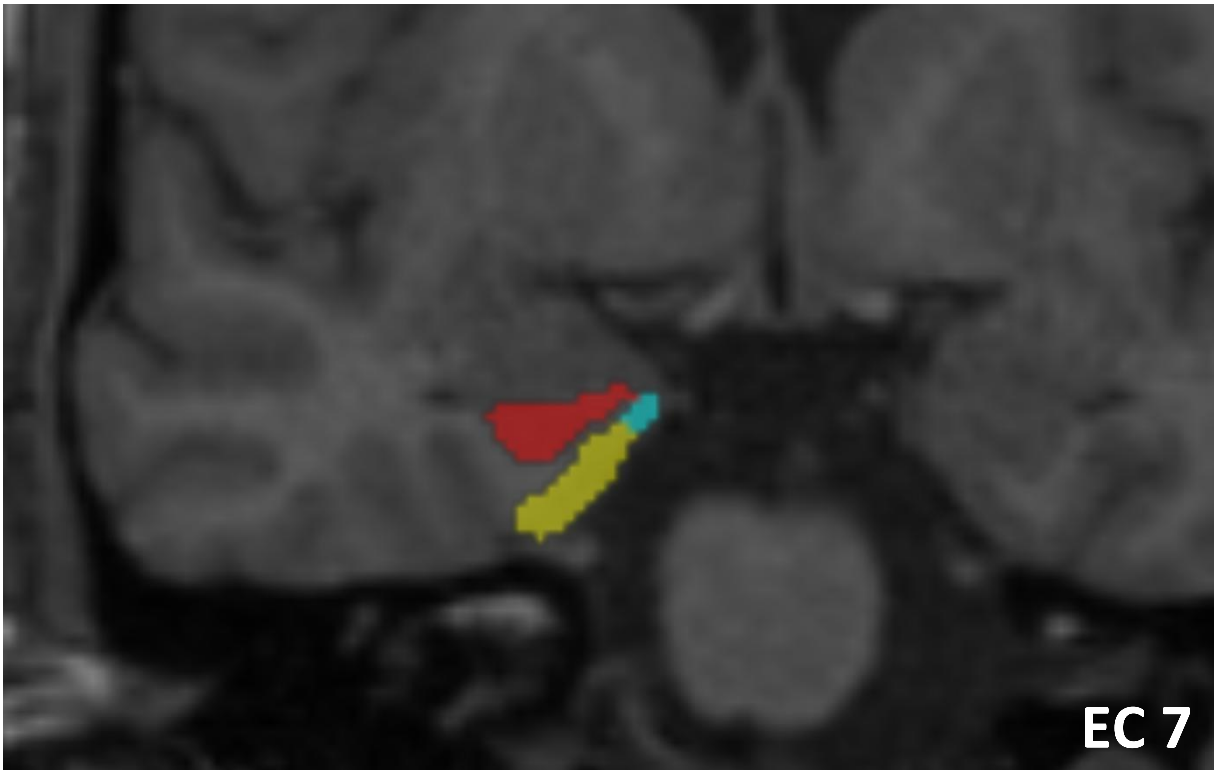
EC 7

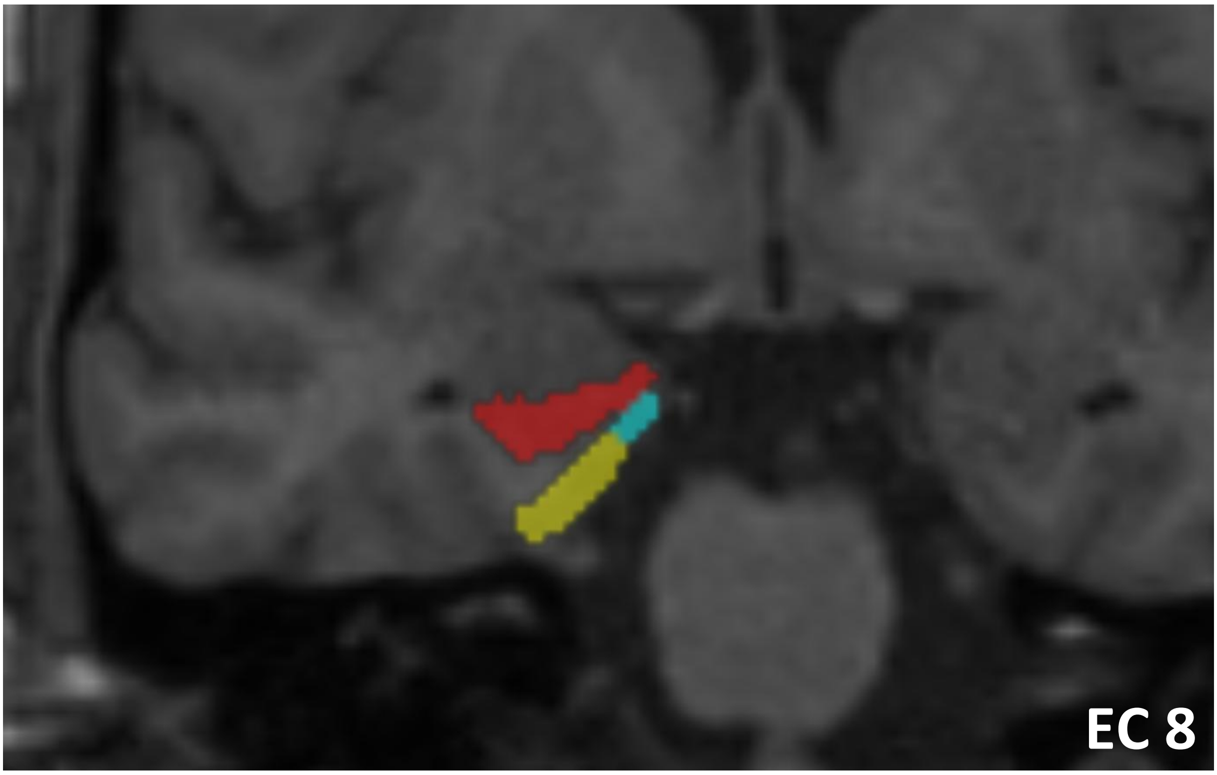
EC 8

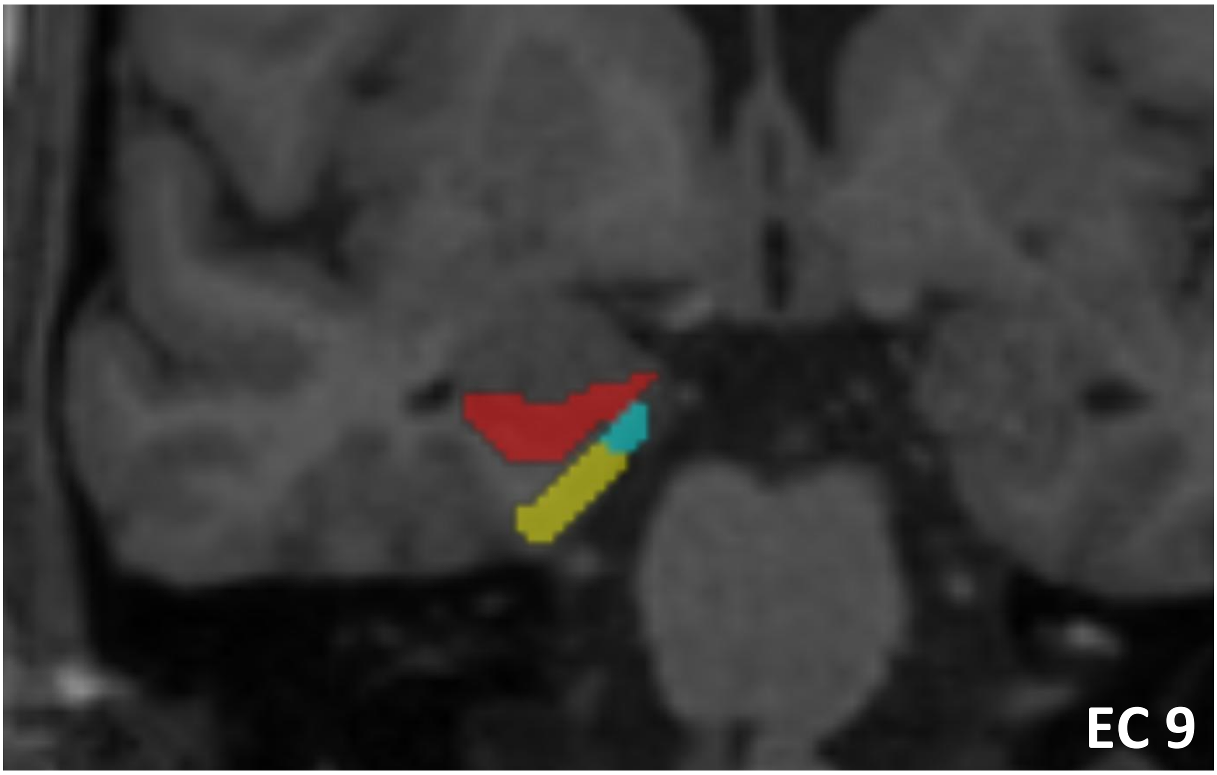
EC 9

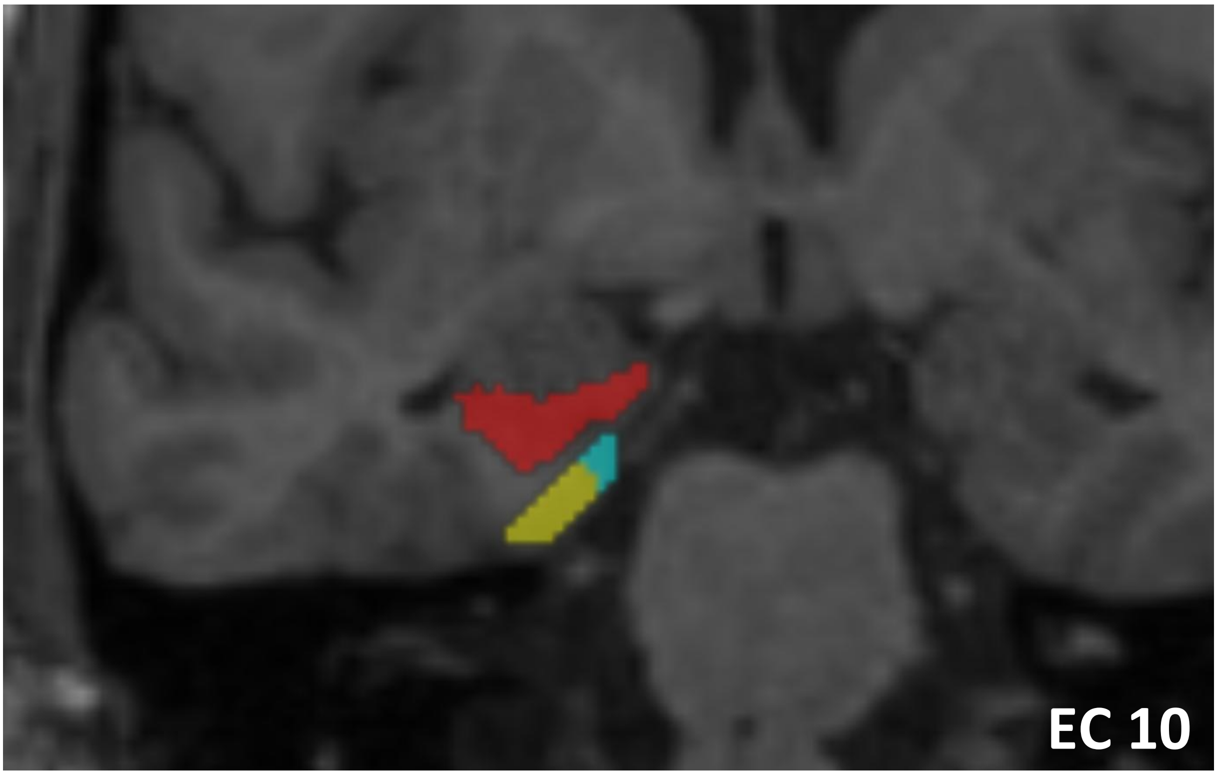
EC 10

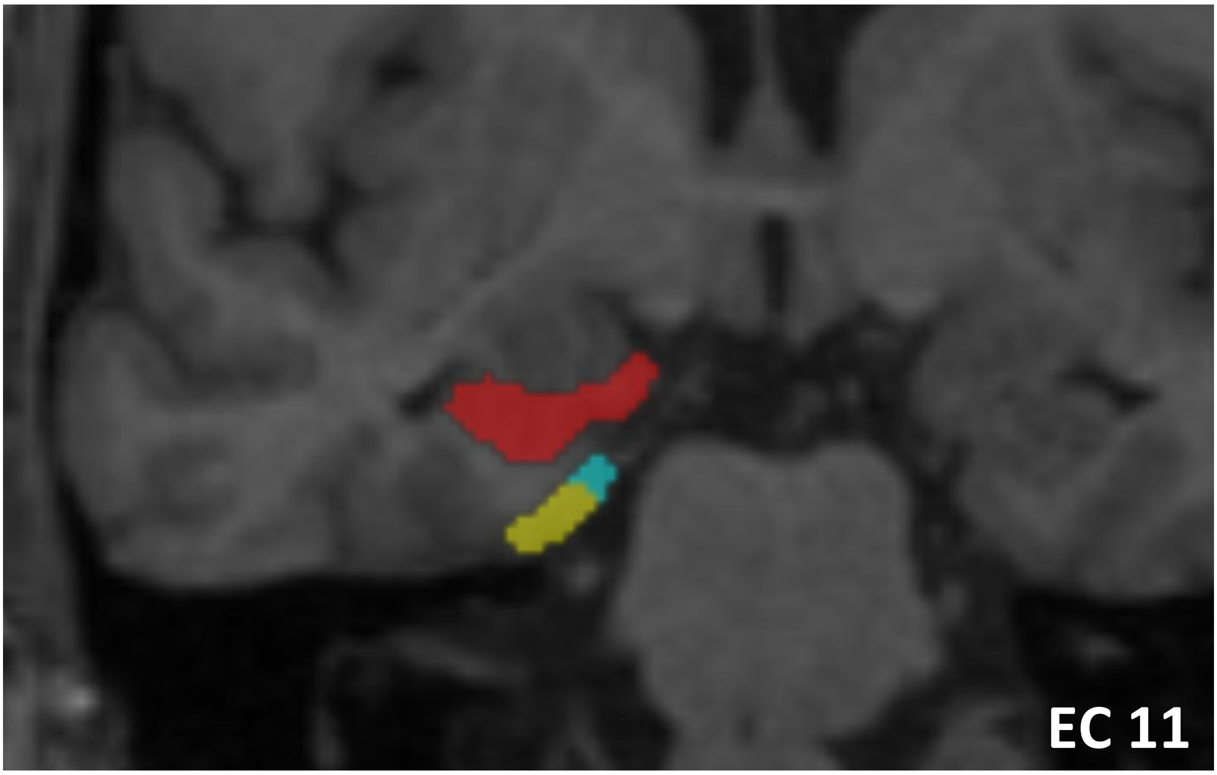
EC 11

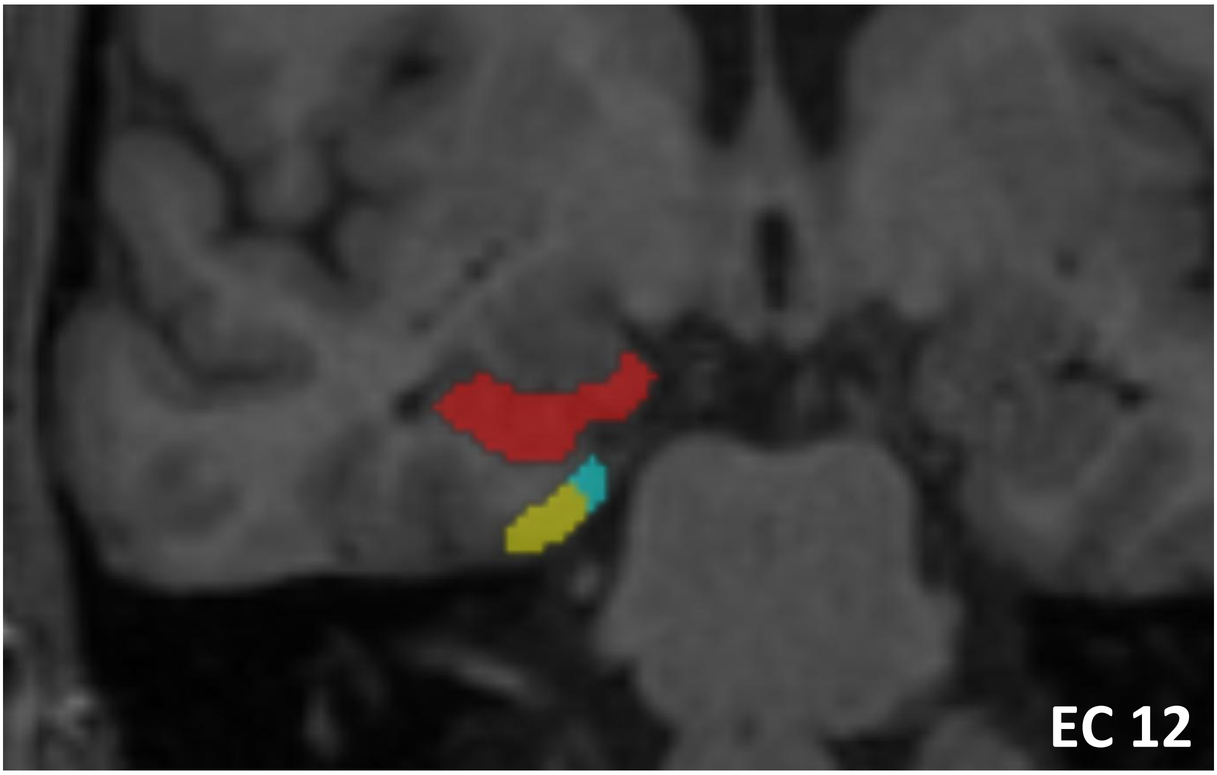
EC 12

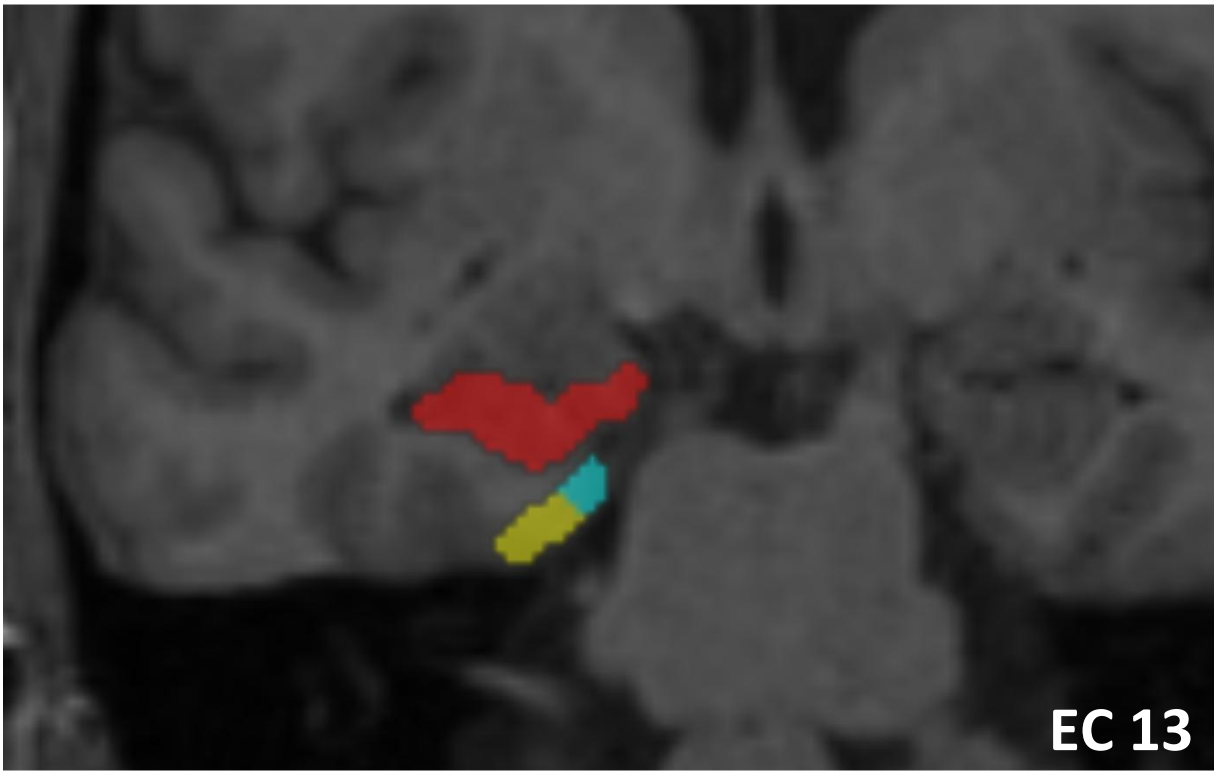
EC 13

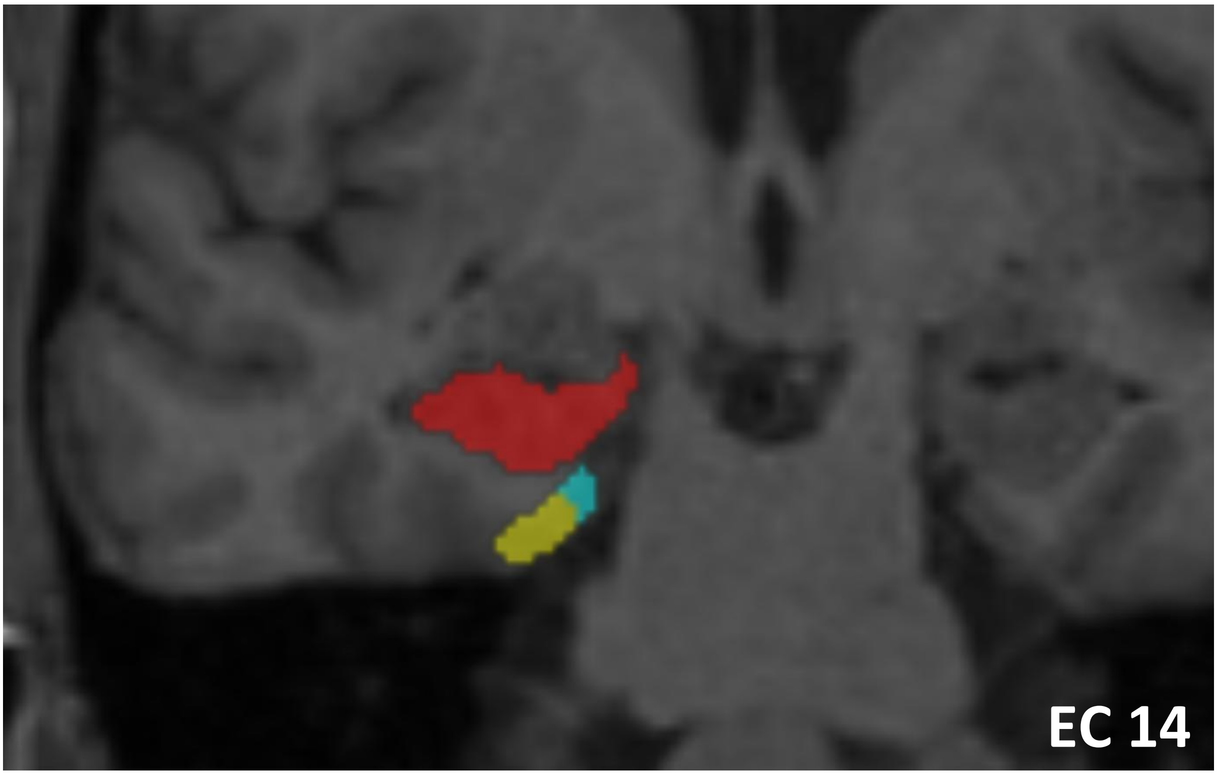
EC 14

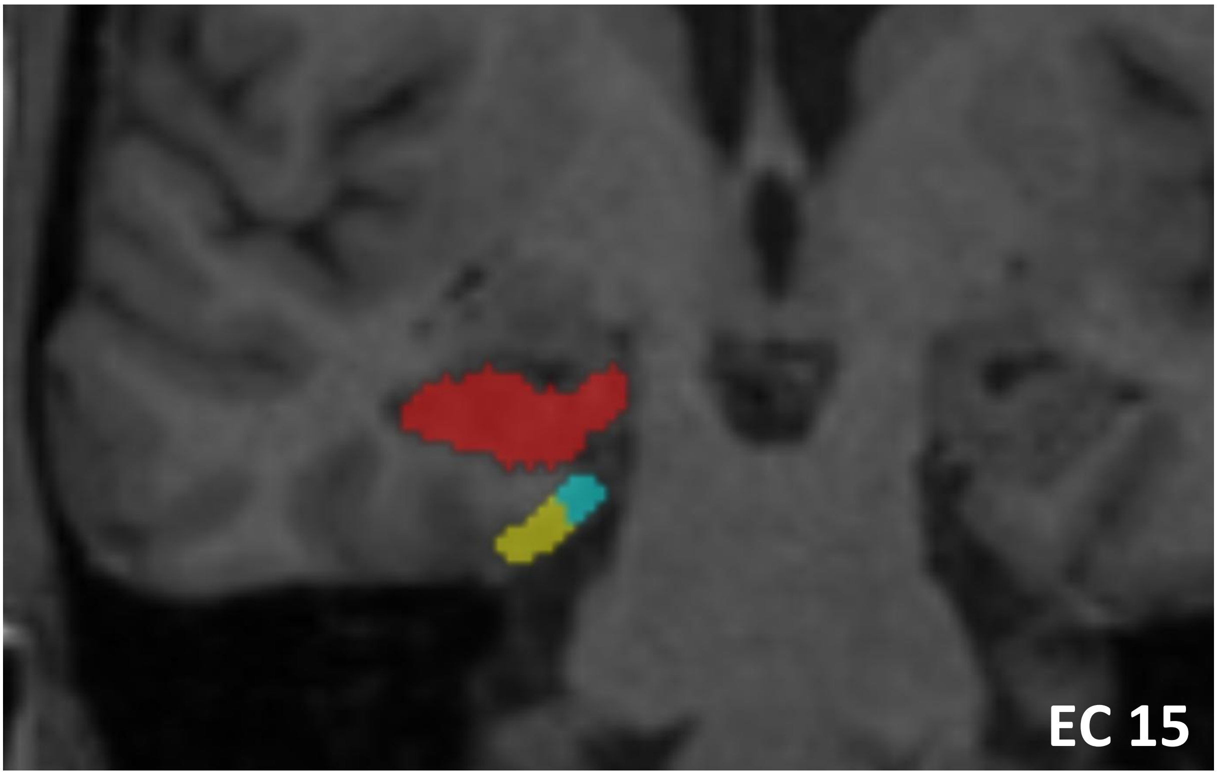
EC 15

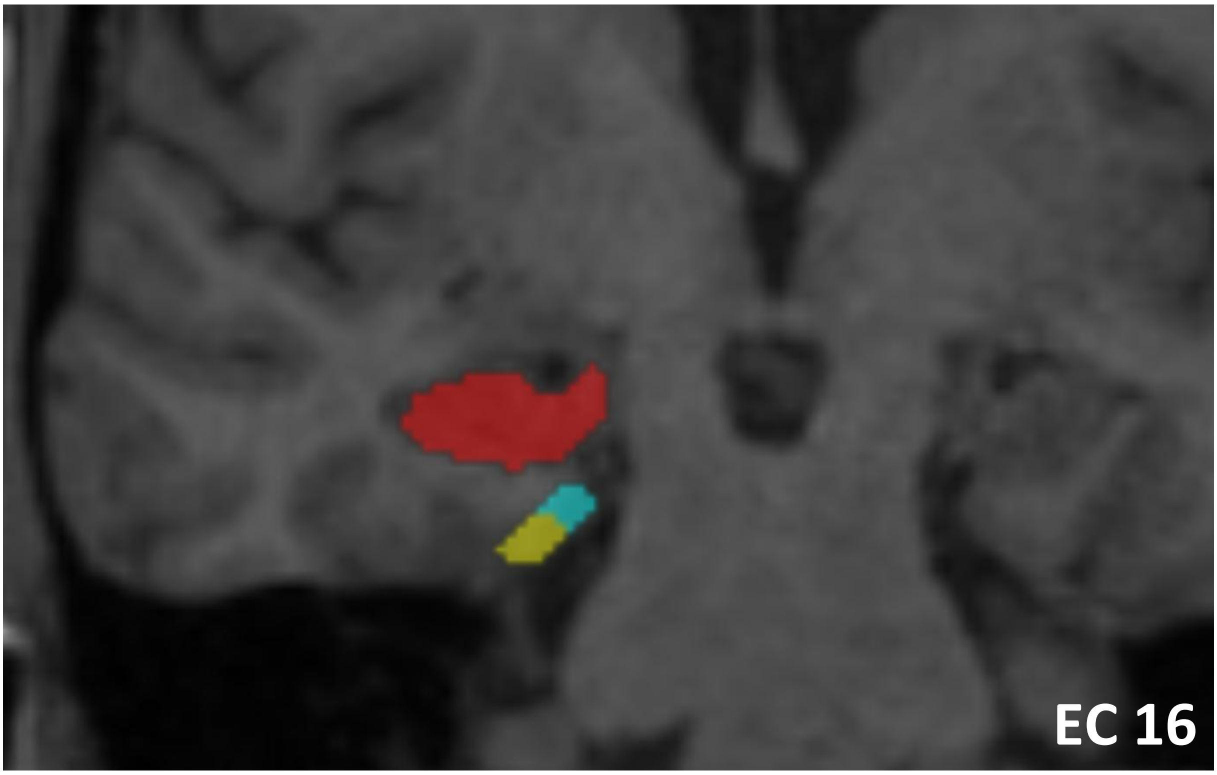
EC 16

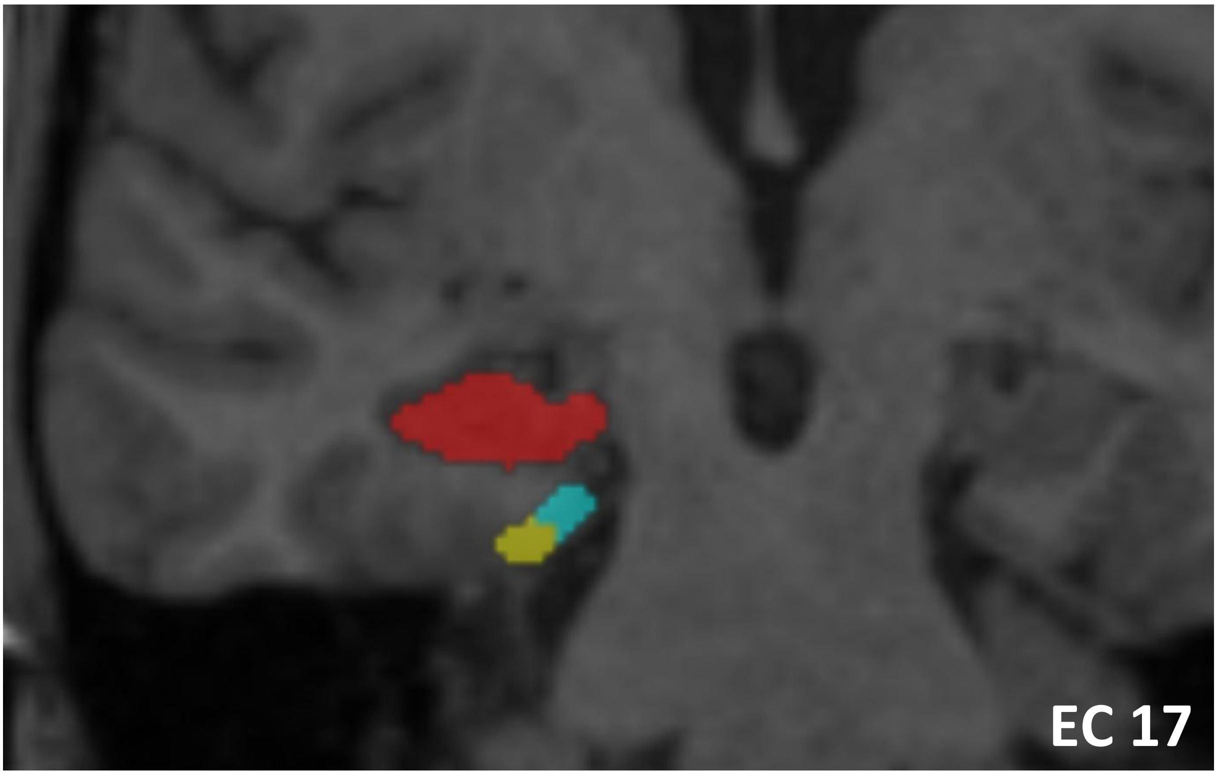
EC 17

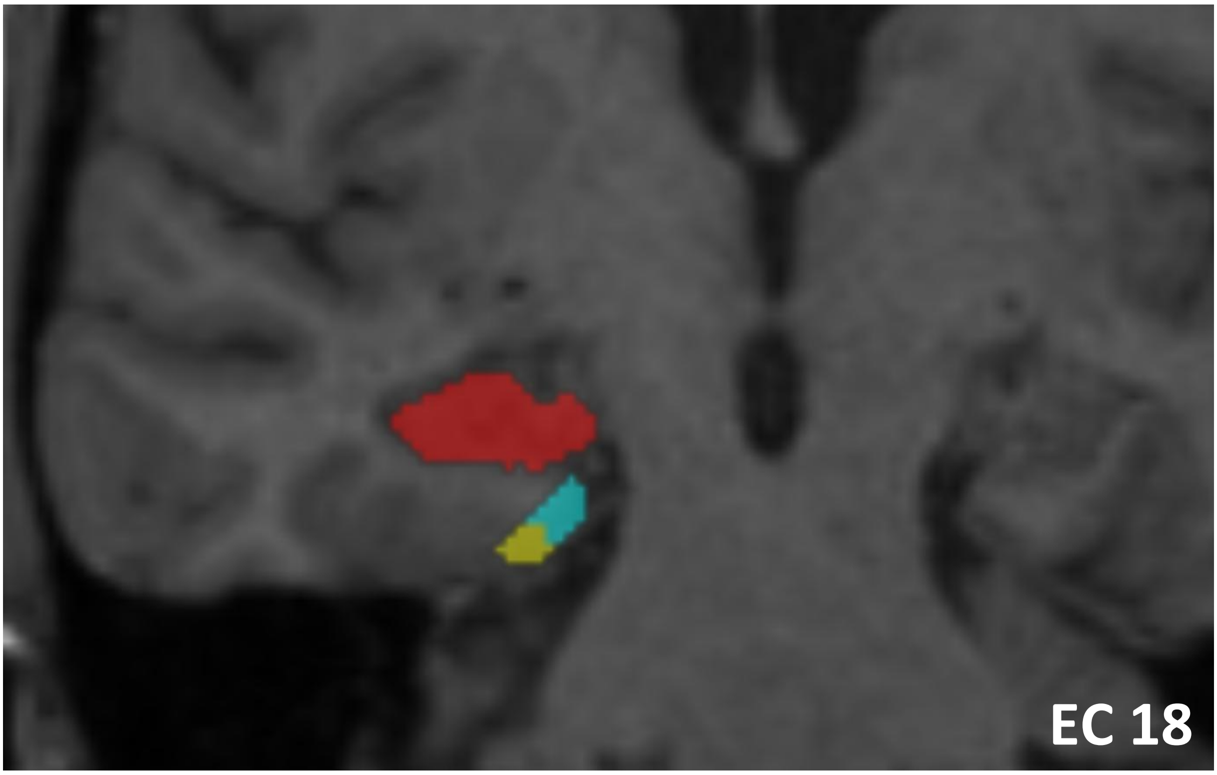
EC 18

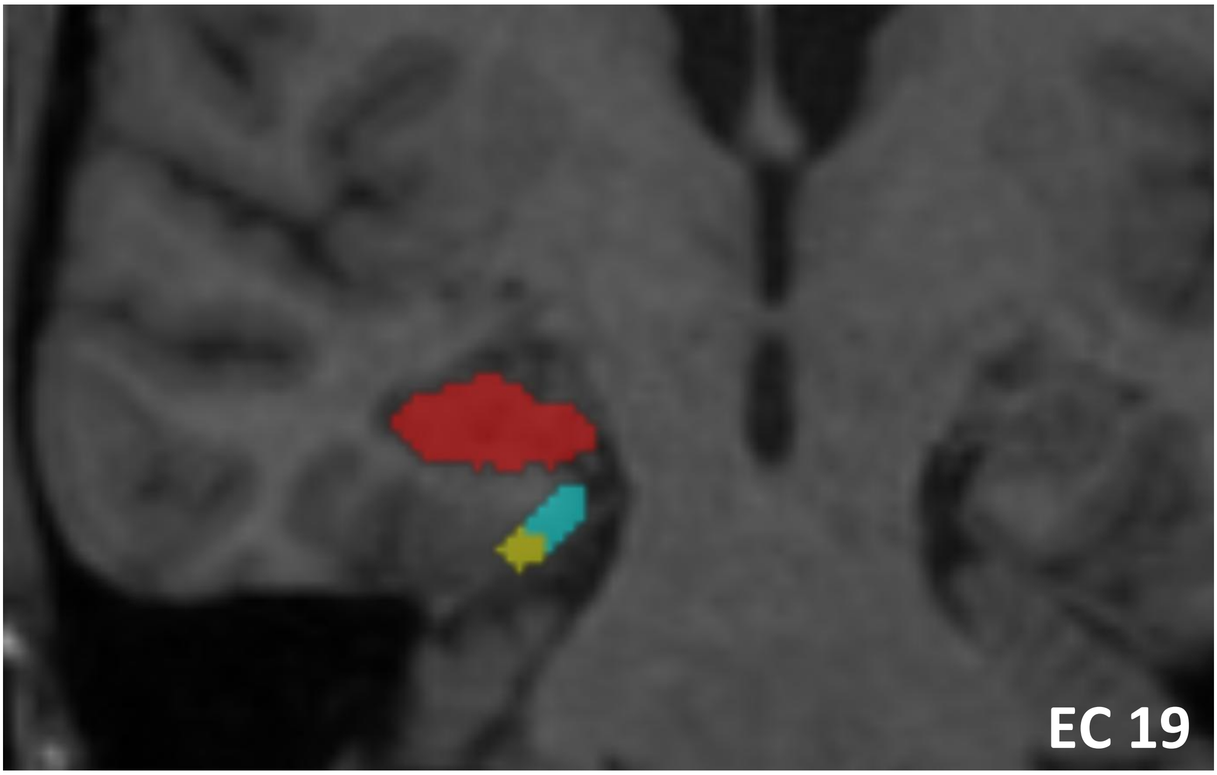
EC 19

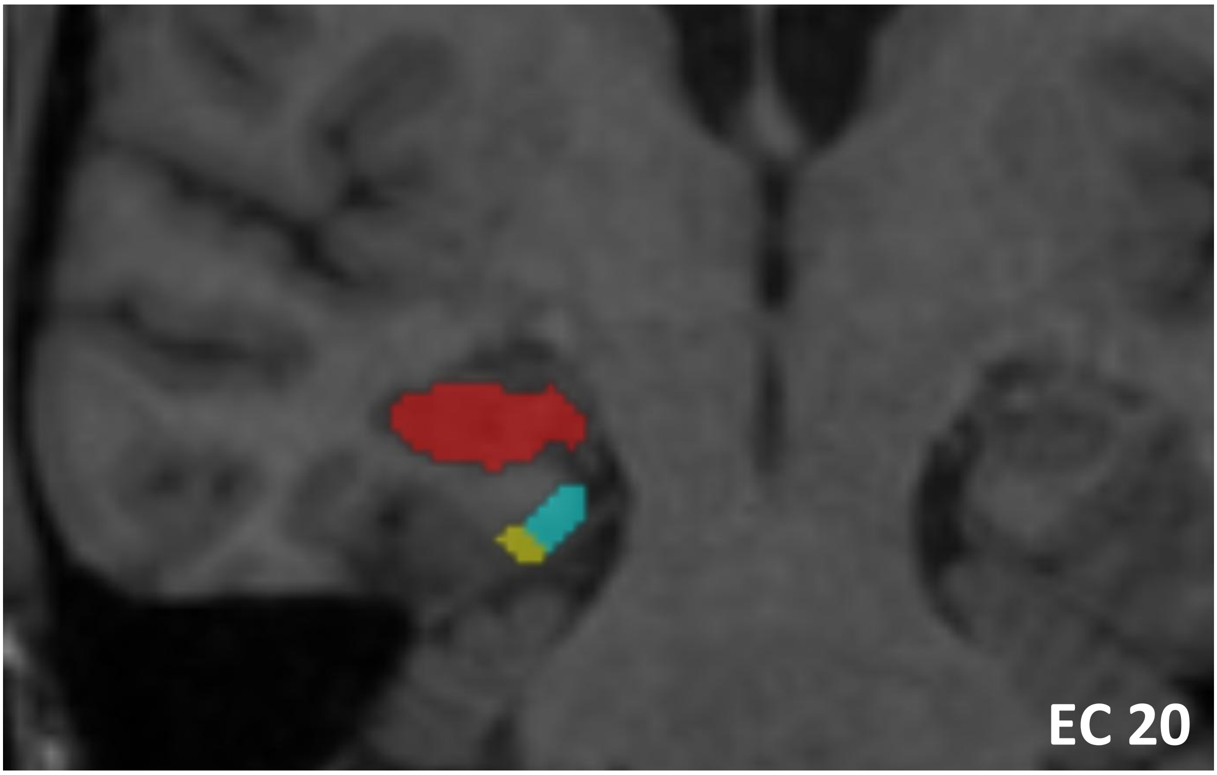
EC 20

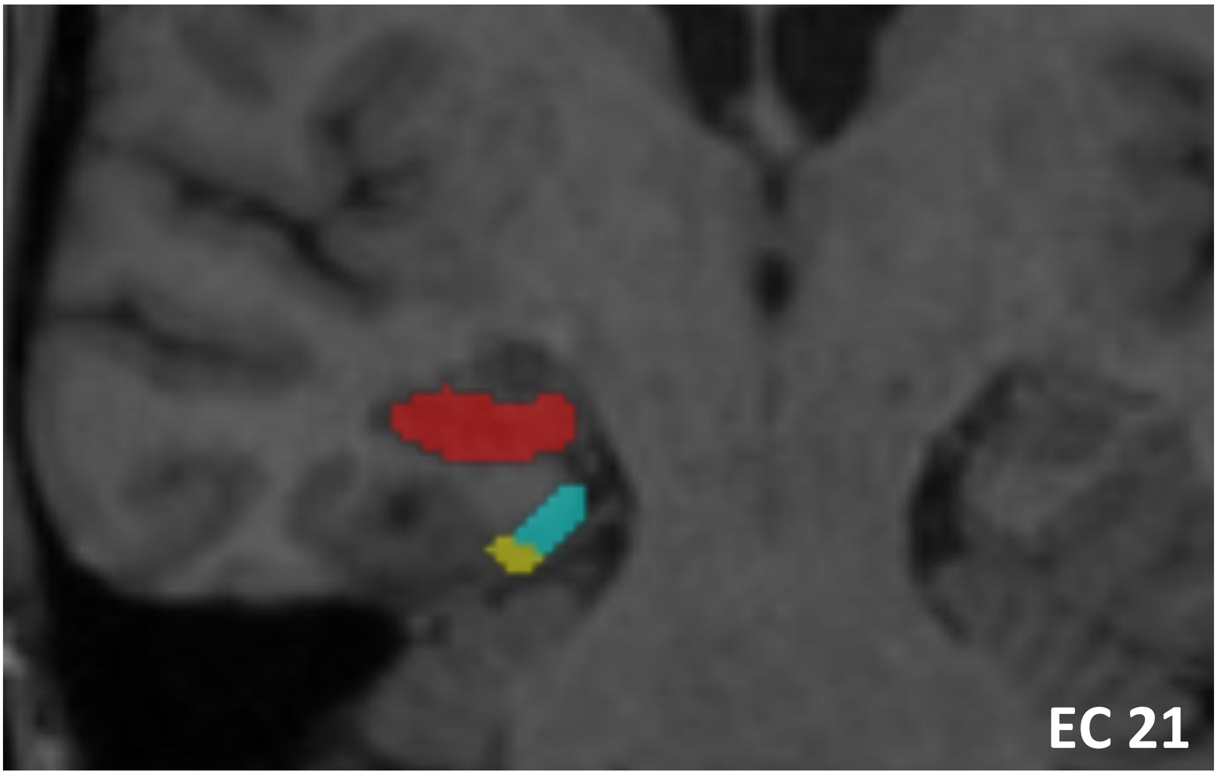
EC 21

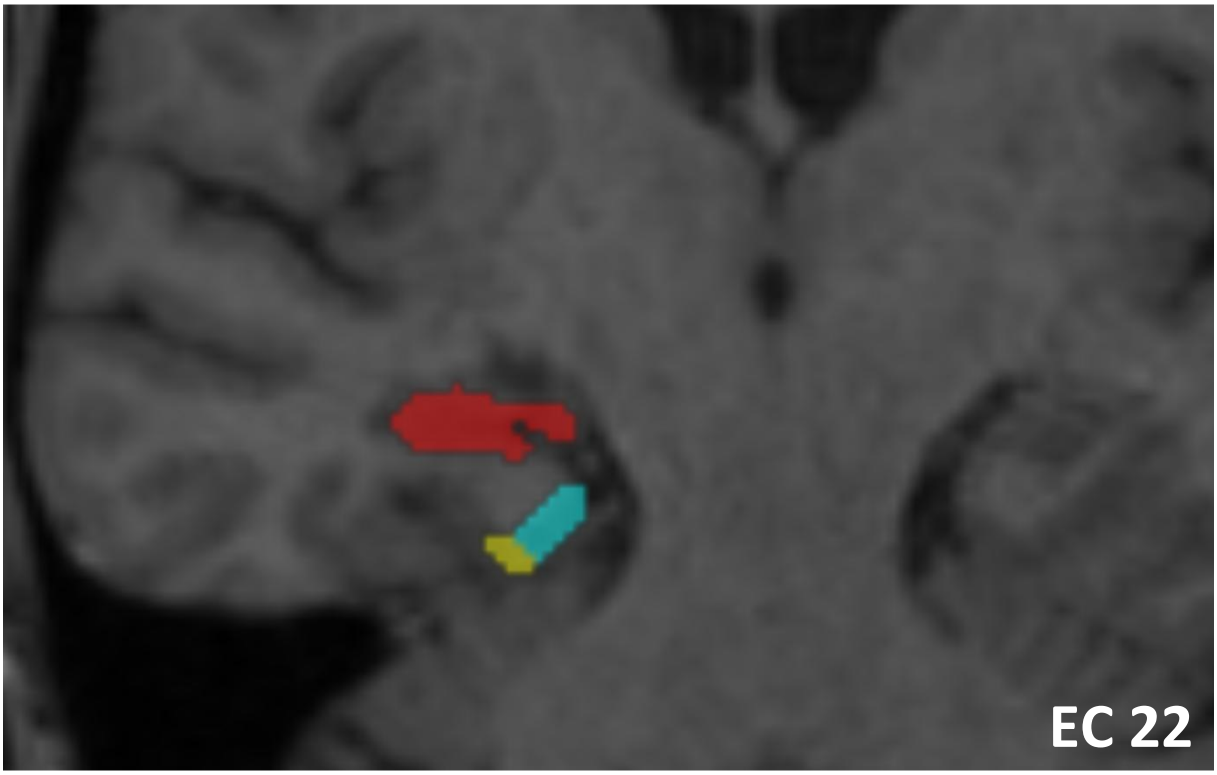
EC 22

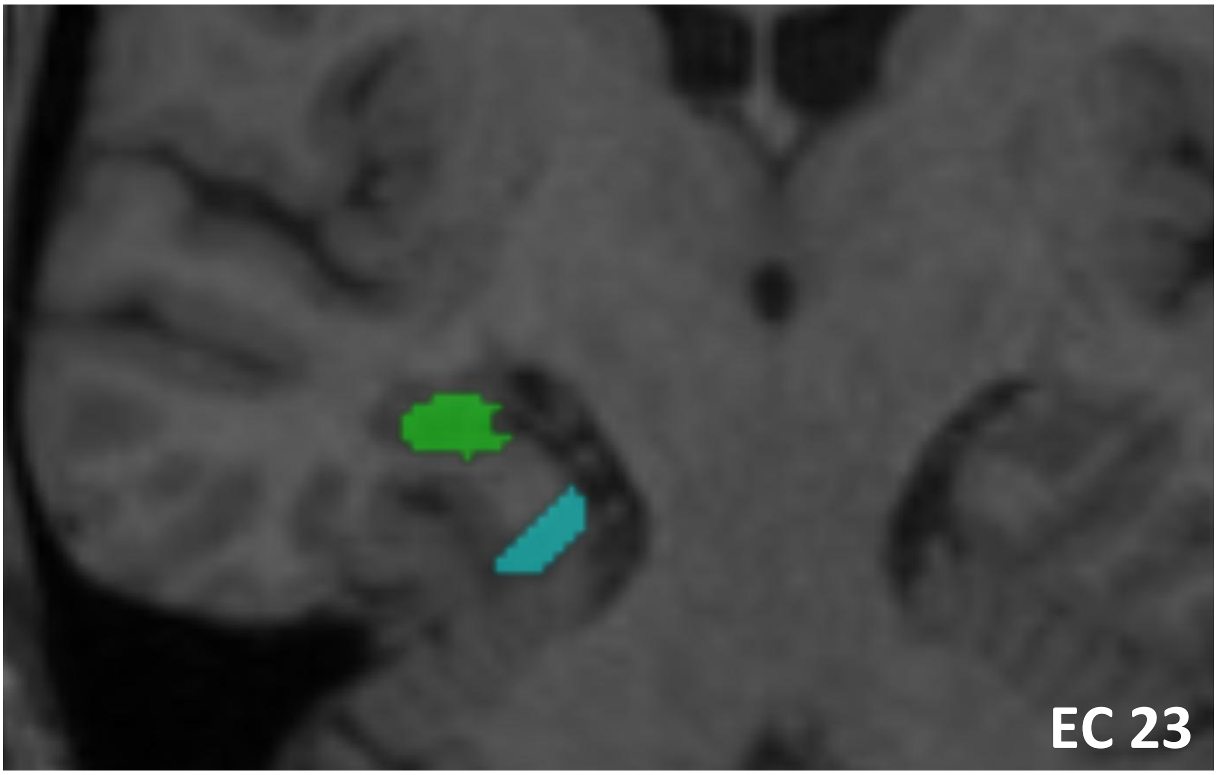
EC 23

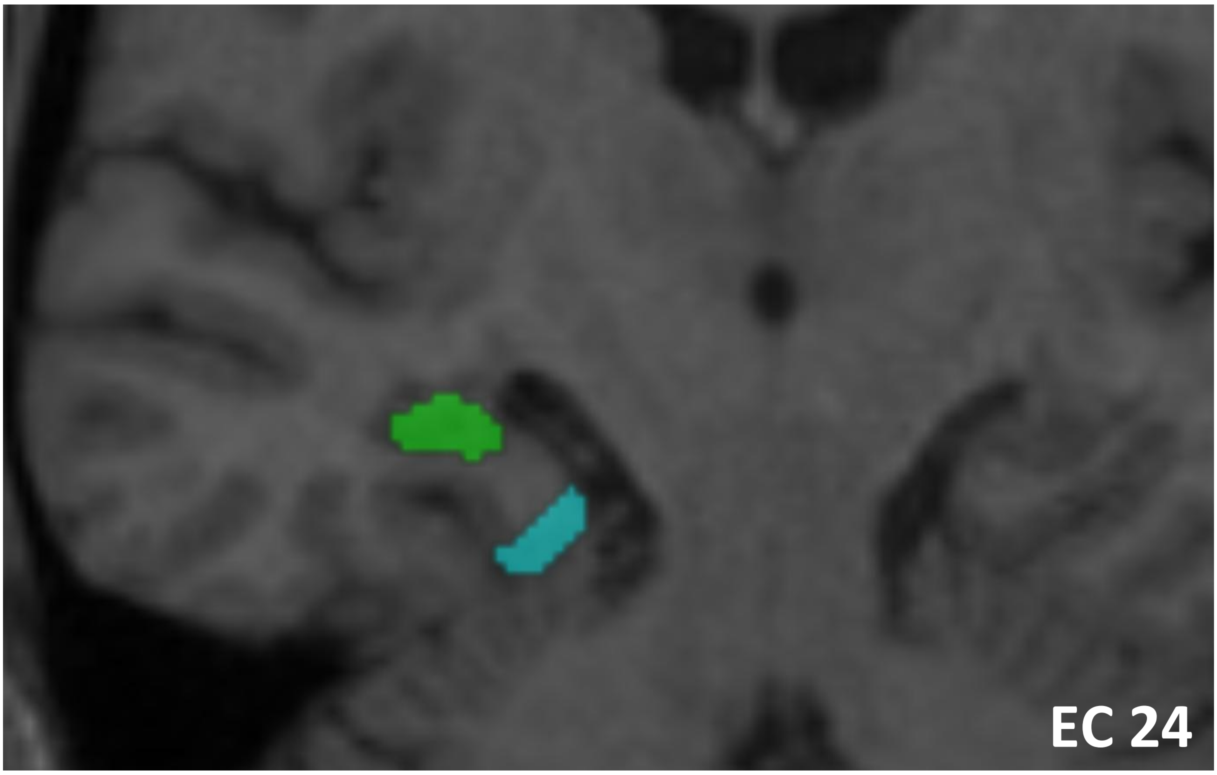
EC 24
